# Single-molecule imaging quantifies oncogenic KRAS dynamics for enhanced accuracy of therapeutic efficacy assessment

**DOI:** 10.1016/j.isci.2025.113374

**Published:** 2025-08-14

**Authors:** Ryoma Yokoi, Toshiki Mori, Koichiro M. Hirosawa, Ryojiro Kondo, Tomohiko Taguchi, Nobuhisa Matsuhashi, Kenichi G.N. Suzuki

**Affiliations:** 1 Department of Gastroenterological Surgery and Pediatric Surgery, Graduate School of Medicine, Gifu University, Gifu 501-1193, Japan; 2 Institute for Glyco-Core Research (iGCORE), Gifu University, Gifu 501-1193, Japan; 3 United Graduate School of Agricultural Science, Gifu University, Gifu 501-1193, Japan; 4 Division of Advanced Bioimaging, National Cancer Center Research Institute, Tokyo 104-0045, Japan; 5 Graduate School of Natural Science and Technology, Gifu University, Gifu 501-1193, Japan; 6 Department of Integrative Life Sciences, Graduate School of Life Sciences, Tohoku University, Sendai 980-8578, Japan

**Keywords:** cell biology, structural biology, cancer

## Abstract

Oncogenic KRAS mutations are frequent in colorectal cancer, presenting substantial challenges due to constitutive activation and resistance to molecular-targeted therapies driven by mutation-specific biochemical properties. In this study, using single-molecule imaging, we quantitatively analyzed diffusional behaviors of oncogenic KRAS mutants and associated signaling molecules to elucidate their signaling mechanisms and therapeutic implications. Upon EGF stimulation, KRAS molecules exhibited transient trapping and reduced diffusion due to interactions with SOS1 and BRAF, leading to the temporary formation of signaling complexes. Our results demonstrate that analysis of the temporal fraction and frequency of transient trapping events offers a more sensitive and precise evaluation of KRAS activation levels than western blotting. Furthermore, the study of dynamics of individual oncogenic KRAS molecules provides a more accurate assessment of the therapeutic efficacy of various molecular-targeted drugs. Consequently, we propose a highly sensitive strategy to enhance the therapeutic targeting of oncogenic KRAS in living colorectal cancer cells.

## Highlights

• Single-molecule imaging reveals KRAS mutant dynamics in cancer
• Transient trapping patterns reflect KRAS interactions with SOS1 and BRAF proteins
• KRAS trapping analysis outperforms traditional western blot methods
• Molecular dynamics inform therapeutic strategies for KRAS-mutant colorectal cancer

## Graphical abstract

**Figure:**
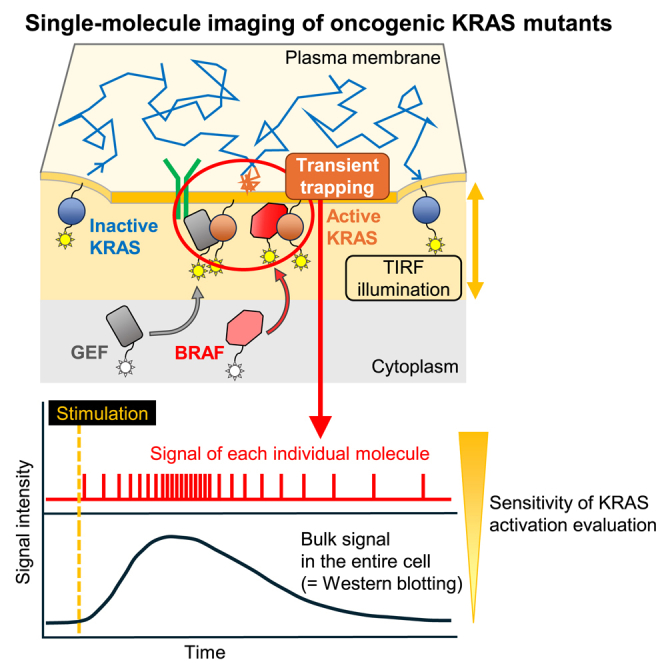

## Introduction

Colorectal cancer (CRC) is the third most prevalent malignancy and the second leading cause of cancer-related mortality globally.^1^^,^^2^^,^^3^ KRAS, one of the three principal RAS isoforms, is a critical driver of CRC initiation. A modified C-terminal lipid moiety anchors this protein to the inner leaflet of the plasma membrane (PM). It alternates between an inactive GDP-bound state and an active GTP-bound state, transmitting signals from cell surface receptors like epidermal growth factor receptor (EGFR) to intracellular pathways that regulate cell proliferation, migration, survival, and metabolism.^4^ Oncogenic mutations in KRAS commonly occur at codons 12, 13, or 61 across various cancers, leading to constitutive activation independent of EGFR signaling and promoting oncogenic signaling through pathways such as the mitogen-activated protein kinase (MAPK) cascade.^5^^,^^6^^,^^7^ While EGFR inhibition is effective in CRC patients with wild-type (WT) KRAS, approximately 40% of metastatic CRC cases harbor oncogenic KRAS mutations, conferring resistance to EGFR inhibitors.^8^ Despite considerable progress in KRAS-targeted therapies, including diverse approaches and combination regimens,^9^^,^^10^^,^^11^^,^^12^^,^^13^^,^^14^^,^^15^^,^^16^^,^^17^ formidable challenges persist due to the genetic and non-genetic complexities of KRAS, interconnected signaling networks, and compensatory feedback mechanisms.^17^^,^^18^^,^^19^ Importantly, KRAS mutations are neither biologically nor prognostically equivalent.^20^^,^^21^ Several clinical studies have reported that specific KRAS alleles, such as G12C, are associated with worse outcomes and differential responses to therapy in CRC.^22^^,^^23^ These challenges underscore the necessity for KRAS-mutation-specific analysis of signaling behavior, which remains insufficiently understood despite its clinical importance.

The activation of KRAS via GDP-GTP exchange is facilitated by guanine nucleotide exchange factors (GEFs), such as Son of Sevenless 1 (SOS1), upon stimulation.^24^^,^^25^ Conversely, KRAS deactivation occurs through GTP hydrolysis, catalyzed by GTPase-activating proteins (GAPs).^5^^,^^25^ Critical amino acid substitutions associated with oncogenic KRAS mutations are primarily located within the active site,^5^^,^^6^^,^^7^^,^^26^ and the activation levels of these mutants are determined by the extent of impaired GTP hydrolysis and enhanced nucleotide exchange, which differ among mutants. Oncogenic KRAS mutations are classified into two categories^6^: those with reduced GTP hydrolysis (class 1, G12) and those with increased nucleotide exchange (class 2, G13). Moreover, the behaviors of oncogenic KRAS mutants reportedly vary even among alleles of the same codon, such as G12D, G12C, and G12V.^7^ Real-time in-cell nuclear magnetic resonance (NMR) studies^27^ have further demonstrated that GTP-bound levels of KRAS in living cells are lower than those observed *in vitro* for both WT and oncogenic mutants. Given the spatiotemporal complexity of signal transduction in living cells, direct quantitative analysis of oncogenic KRAS molecular dynamics within a cellular context is indispensable for elucidating the mechanisms underlying signaling transduction.

Single-molecule imaging is a highly potent technique for investigating molecular dynamics and stochastic events in the PM of living cells.^28^^,^^29^ Studies using single-molecule imaging have revealed that inactive KRAS WT molecules exhibit continuous, rapid lateral Brownian diffusion on the PM,^30^^,^^31^^,^^32^ whereas activated KRAS WT molecules demonstrate slower diffusion and transient trapping lasting less than 1 s, a phenomenon termed transient trapping.^30^ Similarly, oncogenic KRAS mutants, such as G12V^30^^,^^33^ and G12D,^34^ alternate between mobile and immobile phases, with the immobile fraction comprising approximately 10%–20% of the total population. It has been hypothesized that transient trapping involves the formation of temporary KRAS signaling complexes that function as scaffolds for signal transduction.^30^^,^^33^^,^^34^ Notably, the activation of RAF kinase after KRAS binding necessitates homo- or hetero-dimerization of its kinase domain with other RAF family members or kinase suppressor of Ras (KSR).^35^^,^^36^^,^^37^^,^^38^ However, the distinct behaviors of these KRAS mutations remain inadequately characterized, and the mechanisms underlying these molecular dynamics are not fully elucidated. Consequently, we quantitatively analyzed the diffusional behaviors of individual KRAS mutants, as well as RAF and SOS1 molecules, with a focus on transient trapping events before and after stimulation, aiming to uncover insights into the regulation of KRAS signaling mechanisms. Additionally, by employing single-molecule imaging, we evaluated the efficacy of molecular-targeted therapies on KRAS activation with greater precision than conventional biochemical methods such as western blotting. This study offers critical insights for the development of effective therapeutic strategies targeting oncogenic KRAS.

## Results

### Temporal trapping of KRAS upon activation, as revealed by single-molecule imaging

**Figure 1:**
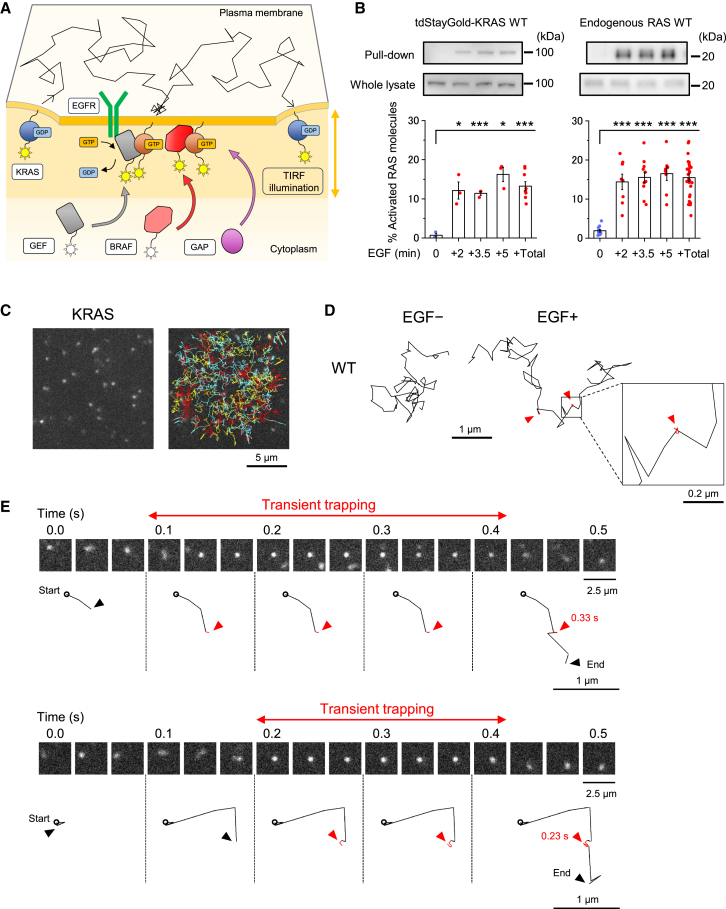
Transient trapping of KRAS upon activation, as revealed by single-molecule imaging (A) Schematic diagram of the experimental setup for single-molecule imaging. (B) Western blot analysis of activated tdStayGold-KRAS (left) and endogenous KRAS (right) obtained by an RAS-GTP pull-down assay, as well as total RAS proteins (including both active and inactive forms) in whole SW48 cell lysates, before and after stimulation with 10 nM EGF. The proportion of activated RAS molecules was quantified based on the western blot data. Independent experimental results are presented, with bars representing the mean ± SEM. Data 2, 3, and 5 min after EGF stimulation were statistically compared with those before EGF stimulation using Welch’s t test. ∗*p* < 0.05 and ∗∗∗*p* < 0.001 (+Total includes all data 2, 3.5, and 5 min after stimulation). (C) Representative still image of single-molecule observation of tdStayGold-KRAS WT in SW48 cells (left), and trajectories of individual molecules acquired from 10-s movies recorded at video rate (33-ms resolution) 3.5 min after EGF stimulation (right, yellow lines: 1–100 frames, red lines: 101–200 frames, blue lines: 201–300 frames; see also Video S1). (D) Representative 3-s trajectories of KRAS WT before (left) and 3.5 min after (right) EGF stimulation, showing transient trapping highlighted by red segments and arrowheads. (E) Image sequences (top) and trajectories (bottom) of individual KRAS molecules, exhibiting transient trapping, highlighted by red segments and arrowheads, alongside their respective trapping durations.

To investigate the dynamic behaviors of KRAS WT, we performed single-molecule observations of transiently expressed tdStayGold-fused molecules localized to the inner leaflet of the PM in living SW48 colon cancer cells. Using total internal reflection fluorescence microscopy (TIRFM) at a video rate (33-ms resolution), we captured molecular dynamics both before and after EGF stimulation (Figure 1A). Before imaging, a KRAS-GTP pull-down assay was performed to verify whether the time course of tdStayGold-KRAS WT activity after 10 nM EGF stimulation aligned closely with that of endogenous RAS WT (Figure 1B). Under serum starvation for 1 h, KRAS WT was nearly inactive. However, the percentage of activated KRAS WT molecules increased markedly in 2 min after stimulation and remained elevated for up to 5 min (Figure 1B, right), closely paralleling the activation profile of tdStayGold-KRAS WT (Figure 1B, left). These results confirm that the tdStayGold fusion has negligible adverse effects on KRAS WT activation.

Next, we performed single-molecule imaging of tdStayGold-KRAS WT, which was sparsely expressed in SW48 colon cancer cells (Figure 1C). Notably, the expression level of tdStayGold-KRAS used for western blotting was comparable to that of endogenous KRAS (10,000–50,000 molecules per cell; Figure 1B). However, for single-molecule imaging, the expression level was kept low (∼3,000 molecules per cell; Figure 1C), rendering tdStayGold-KRAS undetectable by western blotting while enabling continuous tracking of individual fluorescent spots without overlap. Single-molecule imaging revealed that tdStayGold-KRAS WT alternates between phases of diffusion and transient trapping (displayed by the red trajectory and arrowheads in Figure 1D; see also Video S1), with a significant increase in trapping after stimulation. Additional details of this behavior, including image sequences and short (0.5 s) trajectories, are shown in Figure 1E. These results indicate that KRAS WT molecules undergo temporary confinement in small membrane domains upon stimulation, consistent with previous reports.^30^

Video S1. Single-molecule observation of KRAS WT molecules, related to Figure 1

**Figure 2:**
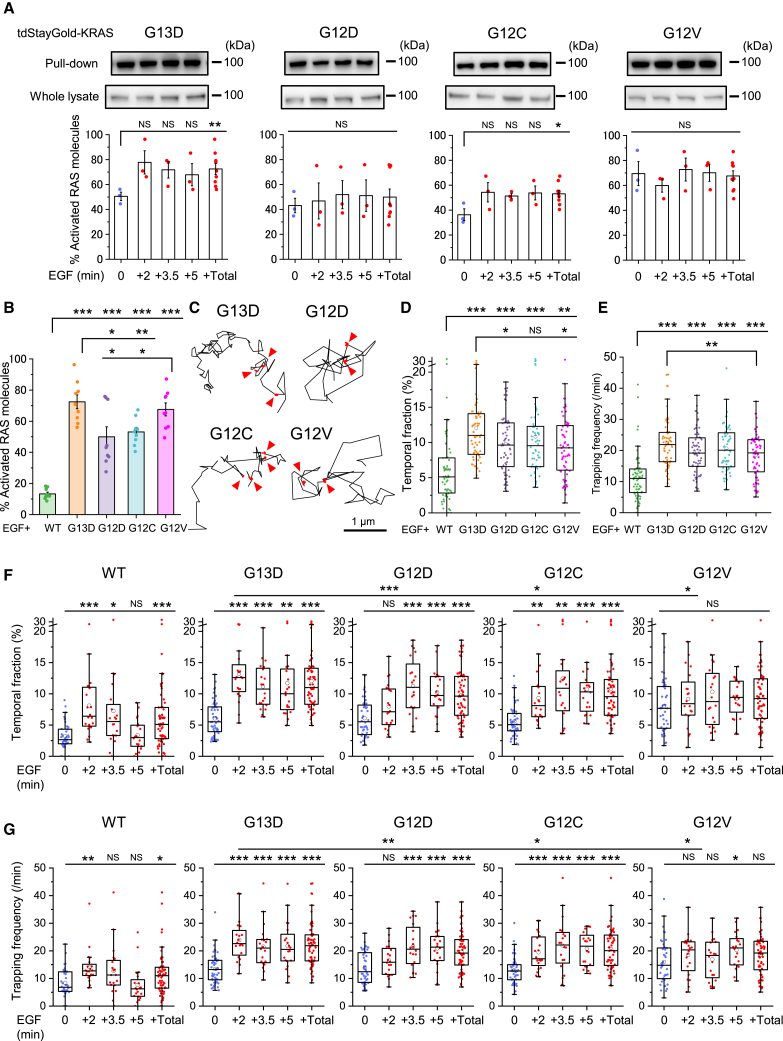
Quantitative analysis of transient trapping of single KRAS WT and mutant molecules reveals activation with greater sensitivity than biochemical assays (A and B) Western blot analysis of activated tdStayGold-KRAS mutant proteins obtained via the RAS-GTP pull-down assay and total tdStayGold-KRAS mutant proteins (including both active and inactive forms) in whole SW48 cell lysates before and after EGF stimulation (top panel in A). The proportion of activated KRAS mutant proteins was quantified based on the western blot data. Results from independent experiments are displayed, with bars representing the mean ± SEM. Data at 2, 3.5, and 5 min after EGF stimulation were statistically compared with those before EGF stimulation (bottom panel in A; +Total includes all data 2, 3.5, and 5 min after stimulation) using Welch’s t test. ∗*p* < 0.05, ∗∗*p* < 0.01, and ∗∗∗*p* < 0.001 throughout this study. Data of KRAS WT and mutants 2–5 min after stimulation (+Total in A) were compared as well (B). (C) Representative 3-s trajectories of oncogenic KRAS mutants after EGF stimulation, showing alternating diffusion and transient trapping (highlighted by red segments and arrowheads, see also Video S2). (D and E) Temporal fractions (D) and frequencies (E) of transient trapping of KRAS WT and mutant proteins observed 2−5 min after EGF stimulation in SW48 cells. (F and G) Time course of temporal fractions (F) and frequencies (G) of KRAS trapping 2, 3.5, and 5 min after EGF stimulation in SW48 cells. The temporal fraction and frequency of trapped molecules are presented as box-and-whisker plots, showing the minimum, maximum, sample median, sample mean (circle), first and third quartiles, and whiskers extending to a maximum of 1.5× interquartile range beyond the box. Data after EGF stimulation were compared either among mutants (D and E) or with those before EGF stimulation (F and G) using Welch’s t test.

Next, we analyzed the temporal activation profiles of oncogenic KRAS mutants using western blotting. The tdStayGold-fused oncogenic KRAS mutants exhibited baseline activation before EGF stimulation. Upon stimulation, the activation levels of G13D and G12C KRAS mutants showed a modest increase (comparison between 0 min and +Total, including +2, +3.5, and +5 min after stimulation, as shown in Figure 2A), whereas the activation levels of G12D and G12V mutants remained unchanged (Figure 2A). The temporal activation profiles of KRAS mutants (Figure 2A) and the average activation levels measured 2–5 min after EGF stimulation (Figure 2B) were highly dependent on the specific mutation, likely reflecting differences in GTP hydrolysis efficiency and/or GDP-GTP exchange activities.

### Quantitative analysis of transient trapping and diffusion coefficients of individual KRAS molecules detects the activation more sensitively

Subsequently, we performed single-molecule imaging of oncogenic KRAS mutants between 2 and 5 min after EGF stimulation in SW48 colon cancer cells that had undergone 1 h of serum starvation. This approach aimed to investigate whether the mutants exhibit temporal trapping in the PM, similar to that observed with KRAS WT. Consistent with KRAS WT, the typical trajectories of all individual oncogenic KRAS mutant molecules exhibited alternating periods of diffusion and transient trapping (shown by the red trajectory and arrowheads in Figure 2C; see also Video S2). These results indicate that single molecules of all examined oncogenic KRAS mutants undergo transient trapping in the inner leaflet of the PM.

Video S2. Single-molecule observation of KRAS G13D molecules, related to Figure 2

To investigate whether the transient trapping of KRAS WT and its mutants correlates with the activation levels, we employed a previously established method,^39^ incorporating our prior modifications,^28^^,^^40^^,^^41^ to statistically detect transient trapping events. We quantitatively assessed the fraction, frequency, duration, and spatial zone size of these events. The analysis included trajectories ≥10 frames, with a 50 nm detection radius and 166 ms (5 frames) threshold residency time. Figures 2D and 2E show the temporal fraction and frequency of transient trapping of KRAS WT and its mutants between 2 and 5 min after EGF stimulation, respectively. Notably, these parameters are dependent on the mutants. Both the temporal fractions and frequencies of transient trapping of all KRAS mutants were approximately 2-fold higher than those observed for KRAS WT (Figures 2D and 2E; *p* < 0.0001, Welch’s t test). The time course of these parameters after EGF stimulation further highlights the distinctions between KRAS WT and the mutant behaviors (Figures 2F and 2G). Specifically, the temporal fraction of KRAS WT trapping peaked at 8.1% 2 min after stimulation and declined to its pre-activation level of 3.4% by 5 min (Figure 2F). On the other hand, the temporal fractions of KRAS mutant trapping before EGF stimulation ranged from approximately 5%–8%, and the values after EGF stimulation peaked at 10%–13%. Therefore, the temporal fractions of all KRAS mutants were significantly higher than those of KRAS WT before stimulation, whereas after EGF stimulation those of G12C and G12V mutants were comparable to those of KRAS WT (Table S1). Upon EGF stimulation, the temporal fraction of KRAS G13D trapping increased sharply with a slope of 3.55 (temporal fraction/min) until 2 min, followed by a decline with a slope of −0.50 (temporal fraction/min; Figure 2F). In contrast, the temporal fractions of KRAS G12D and G12C increased more gradually, with slopes of 1.44 and 1.95 (temporal fraction/min), respectively, until 3.5 min. The temporal fraction of KRAS G12V trapping was already elevated before EGF stimulation (8.0%) and showed no significant increase after stimulation. G13D exhibited the highest trapping temporal fraction and frequency 2 min post-stimulation (Figures 2F and 2G). Temporal changes in KRAS trapping patterns—including the baseline level before stimulation, the rate of increase after EGF stimulation, and the subsequent decline—correlate with mutant-specific variations in nucleotide exchange rates, GTP hydrolysis activity, and activation levels.^6^^,^^7^ Notably, as previously observed, the activation levels of KRAS mutants measured by western blotting showed only modest increases or no significant changes upon EGF stimulation (Figure 2A). These results underscore that analyzing the temporal fractions of transient trapping for individual KRAS WT and mutant molecules provides a more sensitive and precise measure of activation levels than conventional biochemical methods.

The frequency of KRAS trapping showed patterns analogous to those observed in the temporal fraction of trapping events (Figure 2G). Among the mutants, KRAS G13D exhibited the highest frequency of transient trapping at 2 min after EGF stimulation (Figure 2G). Upon stimulation, the frequency of transient trapping increased significantly for G13D, G12D, and G12C, whereas G12V showed either no change or only a marginal increase (Figure 2G). On the other hand, western blotting revealed that the activation levels of KRAS WT and its mutants exhibited only slight increases or no significant changes upon EGF stimulation (comparison between 0 min and +Total; Figure 2A). These results strongly indicate that the frequency of transient trapping for individual KRAS WT and mutant molecules serves as a more sensitive indicator of activation levels than conventional biochemical methods.

**Figure 3:**
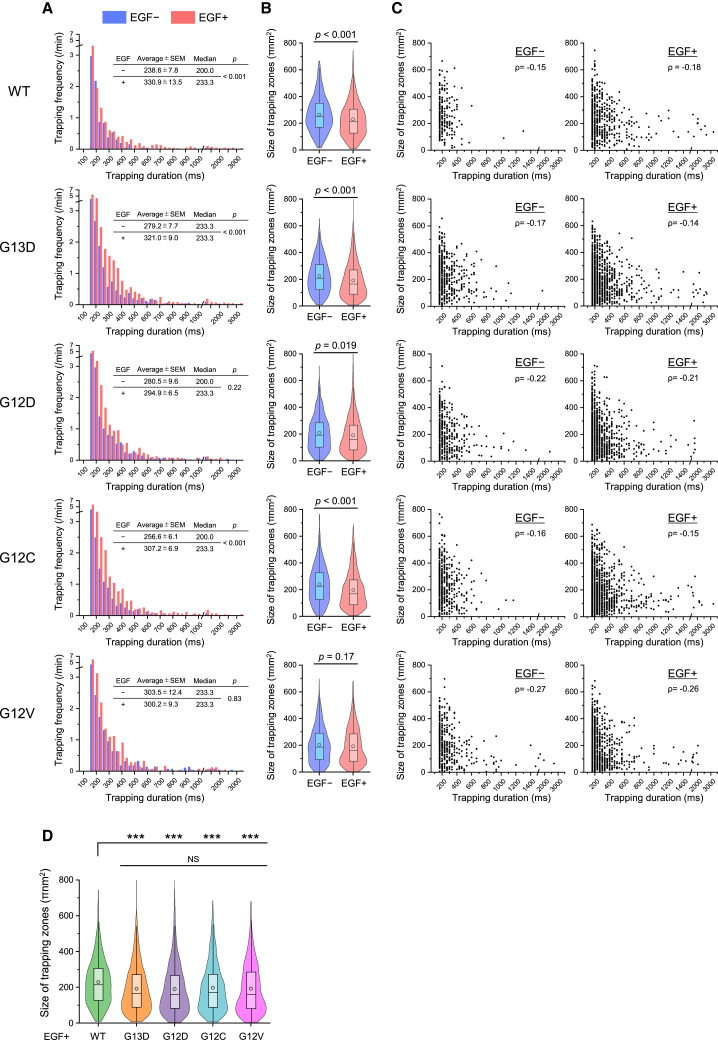
Quantitative analysis of the duration and zone size of KRAS trappings revealed that prolonged KRAS trappings in smaller membrane zones increase upon EGF stimulation (A–C) Distribution of trapping durations (A) and trapping zone sizes (B) of KRAS WT and oncogenic mutants before and after EGF stimulation in SW48 cells. The zone sizes of individual KRAS trapping events are plotted against trapping duration in (C), along with Spearman’s rank correlation coefficient (ρ). (D) Comparison of the trapping zone sizes among KRAS WT and oncogenic mutants after EGF stimulation. The size distribution of individual trapping zones is presented using both violin plots and box-and-whisker plots, showing the sample median, sample mean (circle), first and third quartiles, and whiskers extending to a maximum of 1.5× interquartile range beyond the box. Statistical analysis was performed using Welch’s t test. ∗∗∗*p* < 0.001.

Next, we assessed the duration and zone size of individual KRAS trapping events. Before stimulation, the majority of KRAS WT trapping events lasted less than 500 ms, with an average duration of 239 ± 8 ms (Figure 3A). However, at 2 and 3.5 min after EGF stimulation, trapping durations frequently exceeded 500 ms, with averages of 338 ± 18 ms and 359 ± 30 ms, respectively (Figure S1). The average trapping duration during the 2−5 min window after stimulation was 331 ± 14 ms (Figure 3A). Notably, prolonged trapping events were rarely observed 5 min after stimulation (Figure S1). In contrast, the average trapping durations for all analyzed KRAS oncogenic mutants exceeded 250 ms even before stimulation. Upon EGF stimulation, the average trapping durations of the G13D and G12C mutants increased moderately, whereas those of G12D and G12V mutants showed no significant change (Figures 3A and S1). These results suggest that trapping duration may correlate with KRAS activation levels detected by biochemical methods (Figures 1B and 2A).

Upon EGF stimulation, the average sizes of the trapping zones of KRAS WT and the G13D, G12D, and G12C mutants were significantly reduced, whereas the trapping zone size of G12V remained unchanged (Figure 3B). The sizes of individual trapping zones for KRAS WT and mutants decreased as the duration of individual trapping events increased (Figure 3C). Notably, the average trapping zone sizes of all examined KRAS oncogenic mutants were significantly smaller than that of KRAS WT after stimulation (Figure 3D). As previously mentioned, the change in the activation level of KRAS G12D after stimulation was undetectable by biochemical assays (Figure 2A). These results suggest that the zone size of KRAS trapping may serve as a more sensitive and precise indicator of activation than traditional biochemical methods.

Furthermore, the diffusion coefficients and immobile fractions of KRAS exhibited a strong correlation with the temporal fraction and frequency of KRAS trapping (Figure S2). For example, upon EGF stimulation, the median diffusion coefficient of KRAS WT in the time window of 200 ms (D_200 ms_) significantly decreased (*p* < 0.001, two-sided Mann-Whitney U test) from 0.53 μm^2^/s to 0.45 μm^2^/s, accompanied by an increase in the immobile fraction from 1.5% to 3.2% (Figure S2). Immobile fractions of individual molecules were estimated based on the D_200 ms_ of tdStayGold recombinant molecules immobilized on glass, using the 95th percentile of the observed D_200 ms_ values as a threshold criterion. Notably, the D_200 ms_ values of all analyzed KRAS mutants (G13D, G12D, G12C, and G12V) were significantly lower than that of KRAS WT (*p* < 0.0001). Upon EGF stimulation, the D_200 ms_ values for G13D, G12D, and G12C mutants decreased substantially (*p* < 0.001, *p* = 0.001, and *p* = 0.019, respectively), accompanied by increases in their immobile fractions (Figure S2). In contrast, the D_200 ms_ of G12V did not exhibit a statistically significant change (*p* = 0.11) upon stimulation. These alterations in D_200 ms_ in response to EGF stimulation align with changes in the temporal fraction and frequency of KRAS trapping (Figures 2F and 2G), indicating that D_200 ms_ serves as a reliable and robust indicator of KRAS activation levels.

We also performed single-molecule observations of KRAS in Caco-2 cells to evaluate the generalizability of KRAS dynamic behaviors across different cell lines. Caco-2 cells are characterized by lower EGFR expression levels, resulting in attenuated signaling activity,^42^ whereas SW48 cells harbor a hyperactivating point mutation (G719S) in EGFR^43^ and exhibit overexpression, leading to elevated activation.^44^ Single-molecule observations in Caco-2 cells revealed that, upon EGF stimulation, the D_200 ms_ of KRAS WT and G13D significantly decreased (Figure S3A), accompanied by significant increases in the trapping fraction and frequency for both KRAS WT and G13D, whereas no notable changes were observed for G12V (Figures S3A–S3C). These results are consistent with those obtained in SW48 cells (Figures 2D–2G). Furthermore, upon EGF stimulation, KRAS G13D exhibited significant alterations in the duration and zone size of transient trapping events, whereas KRAS G12V did not (Figures S4A–S4C), aligning with observations in SW48 cells (Figure 3). Collectively, these results suggest that the dynamic behaviors of KRAS molecules are conserved across these cell lines, despite variations in EGFR density.

Taken together, these results demonstrate that single-molecule observations and analyses of the temporal fraction, frequency, and spatial zone size of KRAS trapping, in conjunction with D_200 ms_ measurements, provide a more sensitive and quantitative approach for detecting activation than western blotting (Figures 1 and 2). Additionally, although the average trapping durations show a strong correlation with activation levels as determined by western blotting, they exhibit slightly lower sensitivity in detecting activation (Figure 3).

### PS depletion reduces the transient trapping of KRAS WT and oncogenic mutant molecules

**Figure 4:**
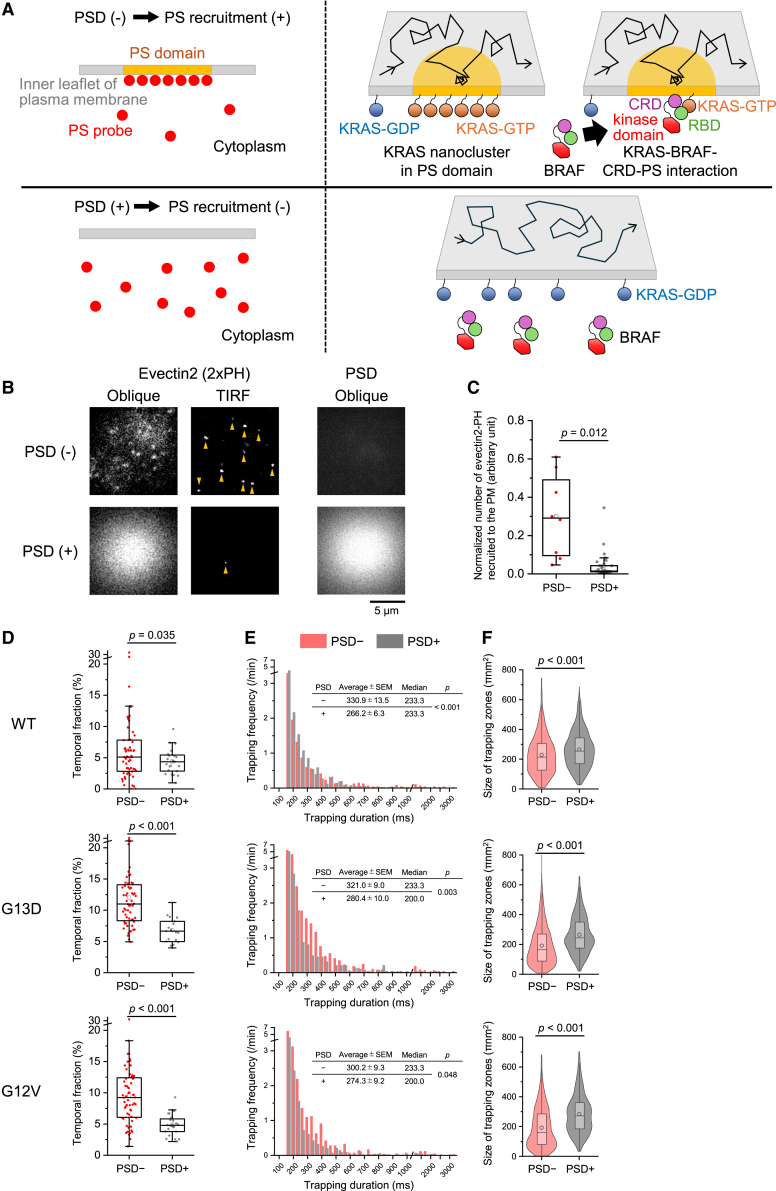
PS depletion attenuates the transient trapping of KRAS WT and oncogenic mutant molecules (A; left) Schematic representation of single-molecule imaging of a PS probe (evectin2 [2xPH]) in SW48 cells, with (bottom) or without (top) PS depletion via PSD expression. (Right) Schematic representation of single-molecule imaging of KRAS in SW48 cells with (bottom) or without (top) PS depletion. Activated KRAS (KRAS-GTP) forms nanoclusters facilitated by PS and/or CRD of BRAF, which associates with PS in the membrane. (B) Fluorescence images of SF650B-Halo7-evectin2 (2xPH) and mCherry-PSD in the presence (bottom) or absence (top) of PSD expression. Images were acquired using oblique angle illumination and TIRFM. Single fluorescent spots of evectin2 (2xPH) recruited to the PM are indicated by yellow arrowheads. (C) Quantification of evectin2 (2xPH) fluorescent spots recruited to the PM with or without PSD expression. Values were normalized to both total probe expression (measured via whole-cell fluorescence under oblique-angle illumination) and the observation area. (D–F) Temporal fractions of transient trapping (D), distributions of trapping durations (E), and trapping zone sizes (F) for KRAS WT, G13D, and G12V, with or without PSD expression, measured 2−5 min after EGF stimulation. The normalized number of recruited PS probe spots and the temporal fraction of trapped molecules are presented as box-and-whisker plots, displaying the minimum, maximum, sample median, sample mean (circle), first and third quartiles, and whiskers extending to a maximum of 1.5× interquartile range beyond the box. The size distribution of individual trapping zones is presented using both violin plots and box-and-whisker plots, illustrating the sample median, sample mean (circle), first and third quartiles, and whiskers extending to a maximum of 1.5× interquartile range beyond the box. Statistical analysis was performed using Welch’s t test.

To elucidate the mechanism underlying KRAS transient trapping, we examined KRAS trapping behavior in SW48 cells after phosphatidylserine (PS) depletion via PS decarboxylase (PSD) expression (Figure 4A, left). PM lipids are known to regulate the spatiotemporal organization of KRAS, with PS playing a pivotal role in the formation of KRAS nanoclusters, which are the critical platforms for KRAS signaling.^45^^,^^46^^,^^47^ Previous studies have shown that depletion of endogenous PS disrupts KRAS nanoclustering.^45^^,^^46^ To validate efficient PS depletion, we co-expressed evectin2 (2xPH), a PS-specific probe,^48^ and assessed its localization using single-molecule imaging. In PSD-expressing cells, the number of fluorescent spots corresponding to evectin2 (2xPH) recruited to the inner leaflet of the PM was markedly diminished relative to control cells, indicating effective PS depletion (Figures 4A–4C). We next assessed the trapping behavior of KRAS WT and oncogenic mutants in PSD-expressing cells. PS depletion significantly reduced the temporal fraction of transient trapping for both KRAS WT and oncogenic mutants (Figure 4D). Furthermore, trapping durations were shortened, and the average size of trapping zones was increased for both KRAS WT and oncogenic mutants (Figures 4E and 4F). These findings suggest that PS depletion attenuates KRAS trapping by disrupting nanocluster formation (Figure 4A, right). Given that KRAS nanoclusters function as essential platforms for effector recruitment, and that the cysteine-rich domain (CRD) of RAF directly binds to PS to facilitate membrane association, it is also conceivable that the reduced trapping of KRAS upon PS depletion is partly attributable to diminished RAF-PM interactions mediated by PS (Figure 4A, right).^35^^,^^37^^,^^38^^,^^49^

### Interaction with SOS1 and BRAF induces transient trapping of KRAS

**Figure 5:**
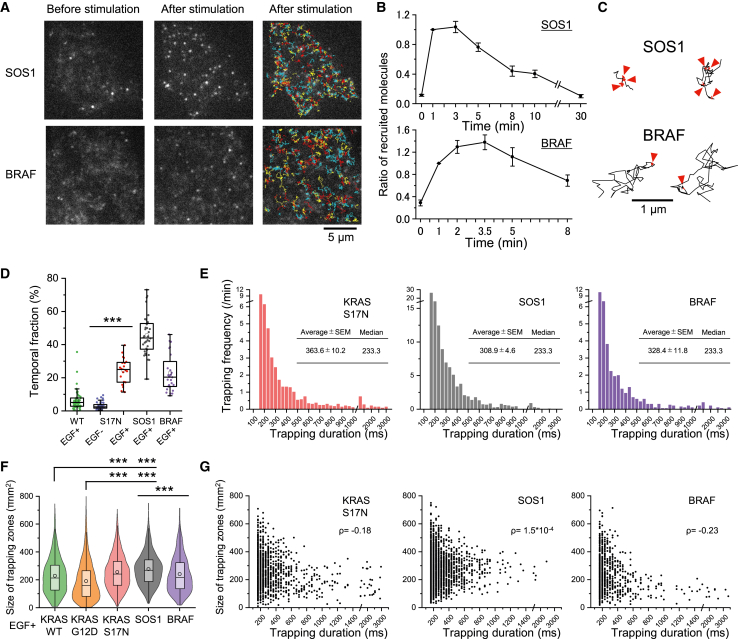
Quantitative analysis of transient trapping of individual SOS1 and BRAF molecules reveals that the association of KRAS with SOS1 and BRAF induces transient trapping (A) Representative images of single-molecule observations of tdStayGold-SOS1 or tdStayGold-BRAF recruited to the PM before and 3 min (SOS1) or 3.5 min (BRAF) after EGF stimulation in SW48 cells (left and middle panels) and trajectories of individual molecules acquired from 10-s movies recorded at video rate (33-ms resolution) after EGF stimulation (right panels, yellow lines: 1–100 frames; red lines: 101–200 frames; blue lines: 201–300 frames; see also Videos S3 and S4). (B) Time course of the recruitment ratio of SOS1 or BRAF molecules to the PM. Data were normalized to values observed 1 min after EGF stimulation and are represented as mean ± SEM. (C) Representative 3-s trajectories of individual SOS1 and BRAF molecules 3 min (SOS1) or 3.5 min (BRAF) after EGF stimulation, exhibiting transient trapping highlighted by red segments and arrowheads. (D) Temporal fraction of transient trapping for single KRAS S17N, SOS1, and BRAF molecules in SW48 cells 1–3 min (SOS1) or 3.5 min (KRAS S17N and BRAF) after EGF stimulation. Data are presented as box-and-whisker plots, displaying the minimum, maximum, sample median, sample mean (circle), first and third quartiles, and whiskers extending to a maximum of 1.5× interquartile range beyond the box. (E–G) Distribution of trapping durations (E) and zone sizes (F) for KRAS S17N, SOS1, and BRAF molecules after EGF stimulation in SW48 cells. The size distribution is presented using both violin plots and box-and-whisker plots, illustrating the sample median, sample mean (circle), first and third quartiles, and whiskers extending to a maximum of 1.5× interquartile range beyond the box. The zone sizes of individual trapping events are plotted against trapping duration in (G) with Spearman’s rank correlation coefficient (ρ). Statistical analyses were performed using Welch’s t test. ∗∗∗*p* < 0.001.

To further elucidate the molecular mechanism underlying KRAS transient trapping, single-molecule observations of SOS1 and BRAF, upstream and downstream effectors of KRAS, respectively, were performed in SW48 cells. After 1 h of serum starvation, the majority of SOS1 and BRAF molecules were localized in the cytoplasm (Figure 5A). Upon EGF stimulation, the number of fluorescent spots at the PM increased by 8.8-fold for SOS1 and 4.7-fold for BRAF (Figures 5A and 5B). These results indicate that, upon activation, SOS1 and BRAF are recruited to the Grb2-EGFR complex and KRAS-GTP in the inner leaflet of the PM, respectively (Figures 5A and 5B; Videos S3 and S4). Single SOS1 molecules were already extensively recruited to the PM at 1 min after EGF stimulation, peaking at 3 min and subsequently declining. The recruitment of single BRAF molecules exhibited a slightly delayed increase, peaking at 3.5 min after EGF stimulation (Figure 5B).

Video S3. Single-molecule observation of SOS1 molecules, related to Figure 5

Video S4. Single-molecule observation of BRAF molecules, related to Figure 5

Single-molecule tracking of SOS1 and BRAF revealed that these molecules alternate between slow diffusion and transient trapping, mirroring the behavior observed in activated KRAS molecules (Figure 5C). Quantitative trajectory analysis showed that the temporal fractions of transient trapping for SOS1 and BRAF were 46% and 23%, respectively, much larger than those observed in activated KRAS WT and oncogenic mutants (Figures 2D and 5D). Furthermore, upon EGF stimulation, the temporal trapping fraction of the dominant-negative KRAS S17N mutant was significantly larger than those of both KRAS WT and oncogenic KRAS mutants (Figures 2D and 5D). While the nucleotide and effector protein affinities of KRAS S17N are considerably lower than those of KRAS WT, its affinity for GEF is 5.4-fold higher (Figure S5A).^50^^,^^51^

We then performed simultaneous dual-color single-molecule imaging of SOS1 and KRAS S17N in SW48 cells to assess their spatial correlation at the PM (Figure S6A). Distances between all molecular pairs were calculated, and their normalized pairwise distance distribution per unit area was plotted, as previously described (Figure S6B).^52^^,^^53^ Upon EGF stimulation, KRAS S17N was locally enriched in close proximity to SOS1 recruited to the PM (Figures S6B and S6C). This quantitative correlation analysis revealed a strong colocalization between SOS1 and KRAS S17N, suggesting that the trapping of KRAS S17N upon stimulation is likely mediated by its direct interaction with activated SOS1.

The trapping duration of SOS1 was 308 ± 5 ms (mean ± SE), comparable to those of activated KRAS (331 ± 14 ms; *p* = 0.123, Welch’s t test) and BRAF (328 ± 12 ms; *p* = 0.122). Meanwhile, the trapping duration of the S17N mutant (363 ± 10 ms of mean ± SE) was significantly longer than those of SOS1 (*p* < 0.0001) and BRAF (*p* = 0.024) and slightly longer than that of activated KRAS WT (*p* = 0.053; Figure 5E). Upon EGF stimulation, the average zone size of the trapping for KRAS WT was similar to that of BRAF (*p* = 0.162) but significantly smaller than those of SOS1 and KRAS S17N (*p* < 0.0001, Figure 5F). As the duration of BRAF trapping increased, the trapping zone size showed a slight reduction (Spearman’s rank correlation coefficient ρ = −0.23), whereas the size of the SOS1 trapping zone remained independent of trapping duration (ρ = 1.5 × 10^−4^; Figure 5G). The median D_200 ms_ values of SOS1 and BRAF were 0.032 μm^2^/s and 0.093 μm^2^/s, respectively, which are 14- and 4.8-fold smaller than those of KRAS WT after stimulation (Figures S2B and S2C). Furthermore, the immobile fractions of KRAS S17N, SOS1, and BRAF were 7.8%, 26.8%, and 11.8%, respectively (Figures S2B and S2C), significantly higher than those of KRAS WT and oncogenic mutants after stimulation. These results suggest the following: (1) both SOS1 and BRAF exhibit slower diffusion and more frequent trapping than KRAS WT and oncogenic mutants; (2) SOS1 and KRAS S17N are frequently colocalized (Figure S6), and both are transiently trapped in larger membrane zones than KRAS WT, oncogenic mutants, and BRAF. These observations give rise to the hypothesis that KRAS trapping in larger zones is mediated by interactions with the upstream signaling molecule SOS1 (GEF), while trapping in smaller zones may result from interactions with the downstream effector BRAF.

To test this hypothesis, we performed simultaneous dual-color single-molecule imaging of BRAF and SOS1 (Figure S6A) and analyzed their spatial correlation (Figure S6B). BRAF was not enriched in PM regions proximal to SOS1 (Figure S6B). The degree of colocalization between SOS1 and BRAF was significantly lower than that between SOS1 and KRAS S17N (*p* = 0.026) and was comparable to that between SOS1 and TM^28^ (a transmembrane domain of the LDL receptor fused with mStayGold), which served as a negative control (*p* = 0.84; Figure S6C). These findings suggest that the transient trapping of SOS1 and BRAF represents distinct events occurring in separate PM regions, likely corresponding to their roles at different stages of the signaling cascade. The observed reduction in average KRAS trapping zone size after EGF stimulation may therefore reflect an increased frequency of KRAS-BRAF interactions due to enhanced KRAS activation.

### KRAS associates with SOS1 and BRAF in the trapping zone

**Figure 6:**
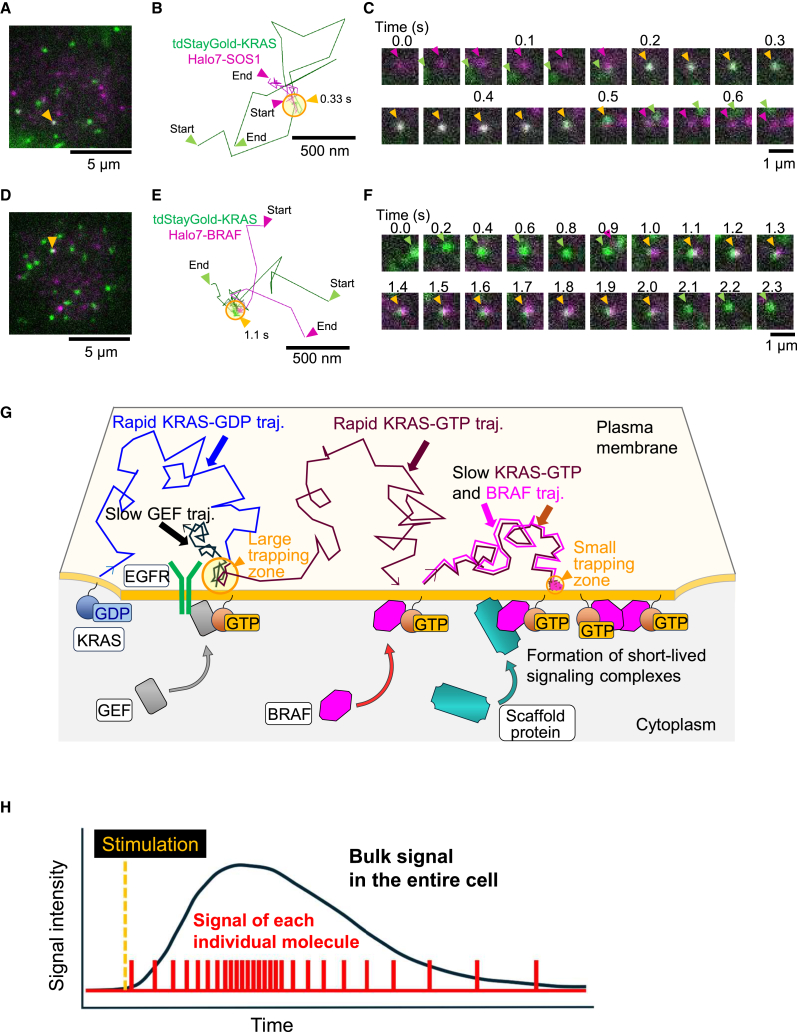
Dual-color single-molecule imaging reveals the association of KRAS with SOS1 and BRAF within trapping zones (A–F) Simultaneous observation of single tdStayGold-KRAS molecules (green) and TMR-Halo7-SOS1 (A–C) or BRAF (D–F) molecules (magenta) after EGF stimulation in SW48 cells. Representative wide-field dual-color images (A and D), trajectories (B and E), and enlarged image sequences (C and F) highlight transient trapping events occurring exclusively during colocalization, as indicated by yellow circles and arrowheads, along with trapping durations (see also Videos S5 and S6). (G) Schematic representation illustrating KRAS, which undergoes transient trapping due to association with GEF and BRAF. (H) Schematic diagram showing how prolonged bulk signaling may arise from the integration of short, pulse-like activation events of individual KRAS molecules.

To test this hypothesis, we performed simultaneous dual-color single-molecule observations of KRAS together with either SOS1 or BRAF in SW48 cells. When single molecules of distinct proteins are located within 240 nm of each other, it is called “colocalization” as previously described.^28^^,^^40^^,^^41^ Our single-molecule analysis revealed that a rapidly diffusing KRAS WT molecule was colocalized with a slowly diffusing SOS1 molecule (Figures 6A–6C; Video S5). During the colocalization period (yellow arrowhead in Figure 6C), the KRAS WT molecule underwent transient trapping, as shown by the yellow circle in Figure 6B. In this instance, the transient trapping zone of KRAS WT colocalized with SOS1 was relatively large (637.1π nm^2^). Similarly, our observations revealed that KRAS WT and BRAF molecules were transiently colocalized, during which both exhibited brief trapping in a small membrane zone (166.2π nm^2^; Figures 6D–6F; Video S6). These results together with those in Figures 5F and 5G, suggest that the transient trapping of KRAS in a larger zone may be driven by its association with SOS1, whereas trapping in a smaller zone arises from interactions with BRAF, thereby supporting the proposed hypothesis.

Video S5. Simultaneous observation of a single KRAS and SOS1 molecule, related to Figure 6

Video S6. Simultaneous observation of a single KRAS and BRAF molecule, related to Figure 6

Based on the single-molecule imaging results, we propose the following model for KRAS signal transduction (Figure 6G): (1) an inactive KRAS-GDP molecule undergoes rapid, simple Brownian diffusion in the PM. (2) Upon EGF stimulation, a SOS1 molecule is recruited to the PM, transiently interacts with a KRAS-GDP molecule, and activates it by facilitating the exchange of GDP for GTP. This interaction reduces KRAS diffusion and causes trapping in larger membrane zones. (3) After dissociation from the SOS1 molecule, the activated KRAS-GTP molecule either resumes rapid diffusion or associates with a BRAF molecule. (4) KRAS-GTP bound to a BRAF molecule undergoes transient trapping in smaller membrane zones, likely due to the temporary formation of signaling complexes involving RAF family dimers or scaffolding proteins such as KSR.^30^^,^^35^^,^^36^^,^^37^^,^^38^ These results explicitly show that the transient trapping of KRAS molecules is a critical process in KRAS activation and signal transduction. The bulk signal intensity of KRAS throughout the entire cell increased within a few minutes after EGF stimulation and gradually declined over approximately 30 min (Figures 1B and 2A). Meanwhile, our results reveal that interactions between individual KRAS molecules and upstream or downstream signaling partners are transient, typically lasting less than 1 s. These observations strongly support our hypothesis of “digital-like signal transduction,” wherein cellular signaling arises from the integration of short-term pulse signals, with their intensity finely tuned by the frequency of transient trapping events.^29^ A comprehensive analysis of both the temporal fraction and zone size of KRAS trapping is crucial for the quantitative assessment of KRAS signaling.

### Cetuximab treatment inhibits the transient trapping of KRAS molecules in a mutation-dependent manner

**Figure 7:**
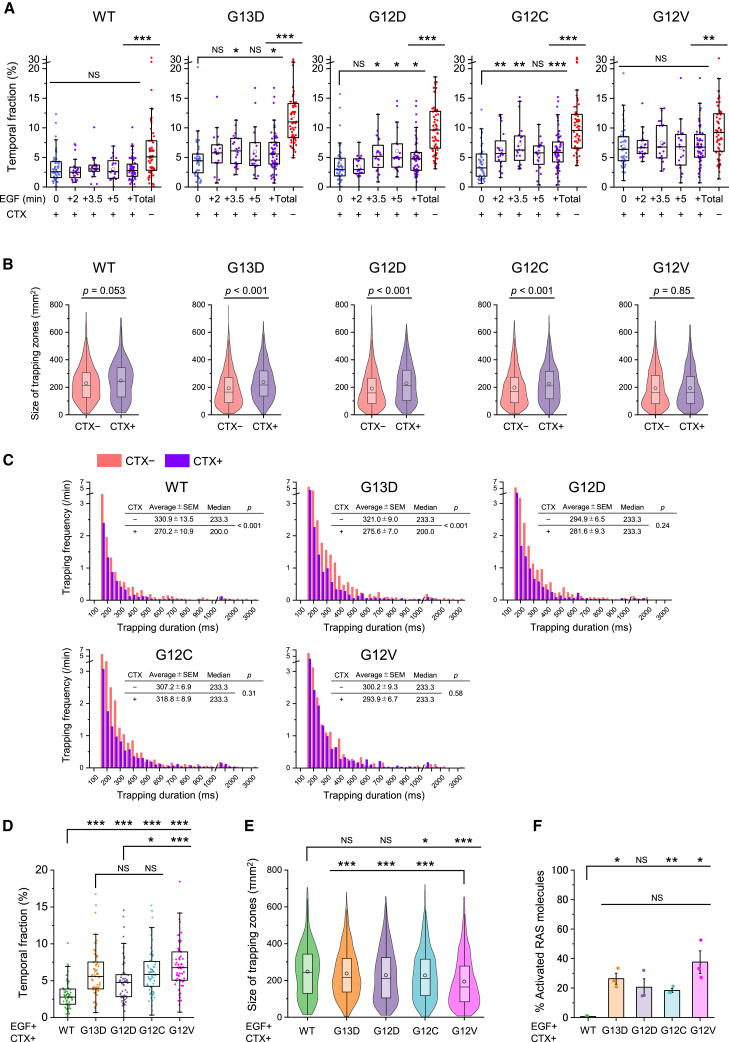
Cetuximab treatment suppresses the transient trapping of KRAS molecules in a mutant-dependent manner (A) Time course of temporal trapping fractions for KRAS WT and oncogenic mutants 2, 3.5, and 5 min after EGF stimulation under cetuximab treatment in SW48 cells (+Total includes all data 2, 3.5, and 5 min after stimulation). (B and C) Distributions of trapping zone sizes (B) and trapping durations (C) for KRAS WT and oncogenic mutants, with and without cetuximab treatment, 2–5 min after EGF stimulation in SW48 cells. (D–F) The temporal trapping fractions (D), trapping zone sizes (E), and their activation levels (quantified from western blot analysis; see Figures S5B and S5C) (F) of KRAS WT and oncogenic mutants in the presence of cetuximab 2–5 min (D and E) and 3.5 min (F) after EGF stimulation. The temporal fraction of trapped molecules is presented using box-and-whisker plots, showing the minimum, maximum, sample median, sample mean (circle), first and third quartiles, and whiskers extending to a maximum of 1.5× interquartile range beyond the box. The size distribution of individual trapping zones is presented using both violin plots and box-and-whisker plots, illustrating the sample median, sample mean (circle), first and third quartiles, and whiskers extending to a maximum of 1.5× interquartile range beyond the box. The percentage of activated KRAS molecules is shown as bar graphs representing the mean ± SEM. Statistical analyses were performed using Welch’s t test. ∗*p* < 0.05, ∗∗*p* < 0.01, and ∗∗∗*p* < 0.001.

Subsequently, we examined the effects of molecular-targeted therapies on the transient trapping of KRAS molecules. Treatment with the EGFR inhibitor cetuximab significantly reduced the temporal fraction of trapping for KRAS WT after stimulation, resulting in no detectable increase in trapping after stimulation (Figure 7A). Additionally, cetuximab treatment slightly decreased the temporal fractions of KRAS G13D, G12D, and G12C trapping before EGF stimulation and led to substantial reductions after stimulation (Figure 7A). Among the oncogene KRAS mutants, KRAS G12V exhibited the highest temporal fraction of trapping (6.9%) in the presence of cetuximab before EGF stimulation; however, this fraction did not increase upon stimulation (Figure 7A). Upon stimulation (+Total, including +2, +3.5, and +5 min after stimulation as shown in the second to the right bars in Figure 7A) in the presence of CTX, KRAS mutants showed higher temporal fractions of trapping than KRAS WT, and KRAS G12V exhibited the highest temporal fraction among KRAS mutants (Figure 7D). The average zone size of KRAS trapping after cetuximab treatment was significantly larger than those in the absence of the drug, except for KRAS WT and G12V (Figure 7B). The average zone size of KRAS G12C and G12V trapping was significantly smaller than that of KRAS WT, and KRAS G12V showed the smallest size among the KRAS mutants (Figure 7E). The results in Figures 7D and 7E are consistent with those of the pull-down assay to estimate the ratio of activated KRAS molecules (Figures 7F, S5B, and S5C). Trapping durations for KRAS WT and G13D were significantly reduced after cetuximab treatment (Figure 7C). However, Spearman’s rank correlation coefficients for the plots of trapping duration versus trapping zone size for oncogenic KRAS mutants remained nearly unchanged after cetuximab treatment, indicating that long-duration, small-sized trapping events persisted among the oncogenic mutants under cetuximab exposure (Figure S7A). Furthermore, cetuximab increased D_200 ms_ values for all mutants except G12V (Figure S8A). In summary, these results indicate that the efficacy of cetuximab treatment was the highest for KRAS WT and the lowest for KRAS G12V, with only minor differences observed among the other three mutants, depending slightly on the evaluation method used.

### Transient trapping of oncogenic KRAS mutants is abrogated by KRAS inhibitors and combined therapies with agents targeting upstream of KRAS, exhibiting additive effects

**Figure 8:**
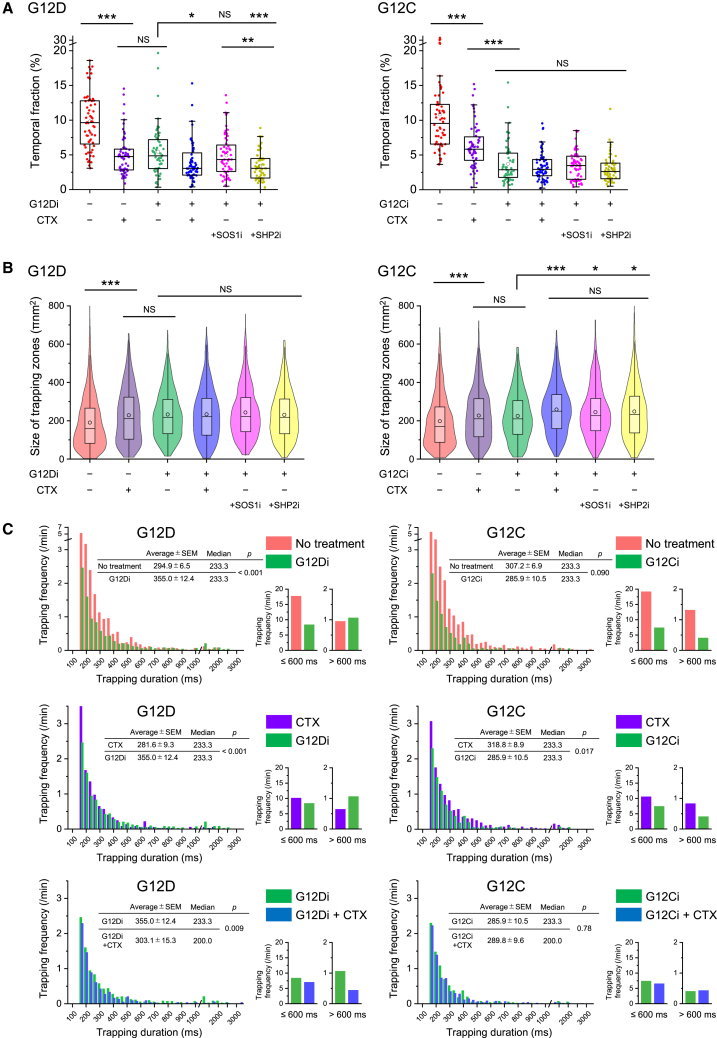
Transient trapping of oncogenic KRAS mutants is disrupted by KRAS inhibitors, and combination therapies targeting upstream regulators of KRAS exhibit additive effects Comparison of the effect of cetuximab, KRAS inhibitor, or combination therapies involving a KRAS inhibitor and cetuximab, SOS1 inhibitor, or SHP2 inhibitor on the transient trapping of KRAS G12D and G12C after EGF stimulation in SW48 cells. (A) The temporal trapping fractions of KRAS 2–5 min after stimulation. Data are presented as box-and-whisker plots, displaying the minimum, maximum, sample median, sample mean (circle), first and third quartiles, and whiskers extending to a maximum of 1.5× interquartile range beyond the box. (B) The trapping zone sizes of KRAS 2–5 min after stimulation. Data are presented as both violin plots and box-and-whisker plots, illustrating the sample median, sample mean (circle), first and third quartiles, and whiskers extending to a maximum of 1.5× interquartile range beyond the box. (C) The trapping durations of KRAS 2–5 min after stimulation. Statistical analyses were performed using Welch’s t test. Significance levels of *p* values comparing KRAS inhibitor monotherapy with each combination therapy, or between combination therapies, were adjusted using Bonferroni correction as follows: ∗*p* < 0.017, ∗∗*p* < 0.003, and ∗∗∗*p* < 0.0003.

Furthermore, we examined the impact of KRAS inhibitors, both as monotherapies and in combination with upstream-targeting agents such as the EGFR inhibitor cetuximab, the SOS1 inhibitor BAY-293, and the SHP2 inhibitor RMC-4550, on the transient trapping of KRAS molecules. The temporal fraction of KRAS G12D trapping was only moderately reduced by the KRAS inhibitor MRTX1133 alone, achieving comparable efficacy to cetuximab, and exhibited a slight increase upon EGF stimulation (Figures 8A and S7B). Combined therapy with KRAS inhibitor plus either cetuximab or SHP2 inhibitor further reduced the temporal fraction of KRAS G12D trapping, while no additional effect was evident when combined with SOS1 inhibitor (Figure 8A). In contrast, the temporal fraction of KRAS G12C trapping was significantly reduced by the KRAS inhibitor adagrasib alone, exceeding the efficacy of cetuximab, although combined therapies did not provide further benefit over KRAS inhibitor monotherapy (Figures 8A and S7B). The pull-down assays revealed that after cetuximab treatment, 20%–40% of activated oncogenic KRAS mutants persisted, whereas KRAS inhibitors, even as monotherapy, nearly eradicated these mutants (Figures S5B–S5E). These results underscore a strong correlation between the temporal fraction of KRAS trapping and changes in activation levels after treatment.

The size of KRAS G12D trapping zones was significantly increased after inhibitor treatment (Figure 8B); however, it remained consistent across all inhibitor combination treatments. In contrast, the size of KRAS G12C trapping zones after KRAS inhibitor treatment was comparable to that observed with cetuximab, but combined therapy with the KRAS inhibitor and cetuximab resulted in an enlarged average trapping size (Figure 8B). Cetuximab treatment reduced both long and short trapping frequencies of KRAS G12D (orange bars in Figure 8C, top-left vs. purple bars in Figure 8C, middle-left), whereas KRAS inhibitor monotherapy primarily suppressed short trapping events (orange bars vs. green bars in Figure 8C, top-left) and extended the average trapping duration—an effect reversed by the combined therapy (green bars vs. blue bars in Figure 8C, bottom left). For KRAS G12C, both short and long trapping events were effectively suppressed by the KRAS inhibitor alone (orange bars vs. green bars in Figure 8C, top-right), with no significant differences observed in outcomes from combined therapy (green bars and blue bars in Figure 8C, bottom-right). These results reveal that long trapping events in small zones are diminished by combined therapy for KRAS G12D, whereas this can be achieved by KRAS inhibitor monotherapy for KRAS G12C, although some residual trapping persisted in both cases (Figure S7A).

The D_200 ms_ values for both KRAS WT and oncogenic mutants after stimulation were increased by treatment with the EGFR inhibitor cetuximab, reaching levels comparable to those observed before EGF stimulation (Figure S8A). Additionally, the KRAS inhibitors for G12D and G12C increased the median D_200 ms_ to 0.58 μm^2^/s and 0.57 μm^2^/s, respectively, exhibiting significantly greater efficacy than cetuximab (Figures S8B top and S8C top). However, the combination of KRAS inhibitors with cetuximab did not further alter the median D_200 ms_ (Figures S8B bottom and S8C bottom), indicating a lack of synergistic effects between these treatments. While cetuximab treatment nearly eradicated activated KRAS WT molecules, 20%–40% of activated oncogenic mutants persisted; these were almost eliminated by KRAS inhibitors, even as monotherapy (Figures S5B–S5E). These results show that the diffusion coefficients of KRAS molecules strongly correlate with the observed changes in activation levels after treatment.

Since the D_200 ms_ values of SOS1 and BRAF were significantly lower than those of KRAS WT and oncogenic mutants (Figures S2A−S2C), their association with SOS1 and BRAF molecules during the activating process reduces the D_200 ms_ of both KRAS WT and oncogenic mutants. Meanwhile, KRAS inhibition with molecular-targeted drugs increased the D_200 ms_ of KRAS G12D and G12C mutants to 0.57–0.58 μm^2^/s, which is significantly higher than the values observed after cetuximab treatment (top panels in Figures S8B and S8C). These results indicate that molecular-targeted drugs substantially diminished KRAS interactions with BRAF. Furthermore, KRAS G12D and G12C mutants, after treatment with either KRAS inhibitors alone or in combination with cetuximab, were largely inactive (Figure S5E), and the median D_200 ms_ values of these molecules (0.57–0.59 μm^2^/s, as shown in Figures S8B and S8C) were comparable (*p* > 0.05) to that of KRAS WT before EGF stimulation (0.53 μm^2^/s, as shown in Figure S2A). Evidently, these drugs not only suppress KRAS activity but also induce conformational changes in KRAS molecules, effectively disrupting interactions with other molecules, including endogenous transient interactors that act independently of EGF stimulation.^11^^,^^12^^,^^13^

In summary, the transient trapping of KRAS G12D and G12C was substantially attenuated by the KRAS inhibitor, with combined therapies demonstrating additive effects. These results demonstrate that single-molecule imaging provides a highly sensitive approach for evaluating the therapeutic efficacy of targeting oncogenic KRAS signaling in living cells.

## Discussion

In this study, we performed a quantitative analysis of the diffusional behavior of oncogenic KRAS mutants, along with upstream and downstream signaling molecules, using single-molecule imaging to elucidate their regulatory mechanisms of activation and signal transduction. Our findings demonstrate that mutation-specific dynamics of individual KRAS molecules and the therapeutic efficacy of molecular-targeted drugs can be quantitatively assessed in living cells with high sensitivity by measuring the temporal fraction, duration, frequency, and zone size of trapping events, as well as the diffusion coefficients. Conventional methods, such as western blotting and fluorescence recovery after photo bleaching (FRAP), capture only ensemble-averaged behaviors of large populations of molecules, and average these behaviors over time and space, thereby limiting their capacity to detect rare and/or transient events. For example, single-molecule imaging in living cells revealed that approximately 6.2% of KRAS WT undergoes transient trapping (Figure 2D), with most trapping events lasting less than 0.5 s upon EGF stimulation (Figure 3A). Such detailed insights cannot be obtained using ensemble-averaging methods. Furthermore, single-molecule imaging demonstrated that the majority of constitutively activated oncogenic KRAS mutants undergo transient trapping due to interactions with upstream and downstream signaling molecules (Figures 1, 2, 3, 4, 5, and 6). Such individual, short-term, digital-like signaling events likely modulate the overall signaling intensity across entire cells by altering the frequency of these discrete signals (Figure 6H). Moreover, EGFR and KRAS molecules are localized in the PM, while signaling molecules such as GEF, GAP, and BRAF are recruited from the cytoplasm to the inner leaflet of the PM upon stimulation, transiently forming complexes during which their mobility becomes restricted.^4^ As shown in the present study, these molecular dynamics are independent of cell lines that we examined. Therefore, single-molecule imaging emerges as a powerful technique for elucidating the transient and intricate nature of intracellular signaling, including processes involving oncogenic KRAS molecules.

Previous studies show that the reduction in the diffusion coefficients of membrane molecules is predominantly attributed to cytoskeletal actin filaments. Transmembrane proteins protrude into the cytoplasm, where their cytoplasmic domains collide with the cytoskeletal actin meshwork.^54^^,^^55^^,^^56^ This interaction induces short-term (1–10 ms) confined diffusion within small domains, approximately 40–230 nm in diameter, in the PMs.^54^^,^^55^^,^^56^ Occasionally, these proteins hop to adjacent domains, where phospholipids undergo confinement again. This behavior, termed “hop diffusion,” can be observed exclusively by ultrafast single-molecule imaging.^54^^,^^55^^,^^56^ This model is called the “membrane-skeleton (MSK) fence model.” Furthermore, ultrafast single-molecule imaging has shown that transmembrane proteins anchored to the actin-based membrane skeleton, termed “pickets,” impede the diffusion of both proteins and lipids in the PM, a mechanism known as the “picket” model.^57^ Ultrafast single-molecule observation revealed that EGFR undergoes hop diffusion between compartments of 106 nm both before and after EGF stimulation in T24 cell PMs, with the residency time in the compartments extending from 18 to 27 ms upon stimulation.^57^ SOS1 molecules are recruited to and co-diffuse with activated EGFR via the adaptor protein Grb2, which is regulated by negative feedback through ERK activation. Upon phosphorylation by ERK, SOS1 molecules dissociate from Grb2.^24^^,^^25^^,^^58^ Upon EGF stimulation, EGFR forms stable dimers and larger oligomers necessary for self-phosphorylation and activation, which leads to an extended residency time within each compartment due to a reduced hop frequency.^29^^,^^57^^,^^59^^,^^60^ As a result, liganded EGFR molecules exhibit slower diffusion. These results align well with our observation that SOS1 molecules display reduced diffusion coefficients (Figure S2B).

Raft association also retards the lateral diffusion of membrane molecules. However, this effect is observed only when large phase-separated liquid-ordered (L_o_) and liquid-disordered (L_d_) domains are present in the membrane. For example, the diffusion coefficients of lipids within micron-scale raft-like L_o_ domains, measured in L_o_-L_d_-phase-separated giant unilamellar vesicles and giant PM vesicles, are reduced by factors of five to ten^41^ and two,^61^ respectively, compared to those in L_d_ domains. In contrast, raft association in living cell plasma membranes hardly changes the diffusion coefficients,^41^^,^^62^ likely due to the nanoscale size of rafts. Therefore, raft association is unlikely to affect the diffusion coefficients of KRAS WT and oncogenic mutants.

Our findings indicate that KRAS molecules interact with SOS1 (Figures 6A–6C) and BRAF molecules (Figures 6D–6F), resulting in reduced diffusion and increased transient trapping, which may serve as a critical indicator for characterizing signal transduction (Figure 6G). The zone size distribution of KRAS trapping (Figures 3B, 3D, 7B, 8B, and S4B) and the diffusion coefficients (Figures S2A, S3A, and S8A–S8C) were altered upon treatment with inhibitors, irrespective of cell type. KRAS is anchored to the inner leaflet of the PM via a prenyl chain, and its diffusion is likely impeded by a row of pickets aligned with the cortical actin meshwork. Notably, scaffold proteins involved in KRAS signaling, such as kinase suppressor of Ras1 (KSR1)^63^ and Ras GTPase-activating-like protein (IQGAP1),^64^ are associated with cytoskeletal actin filaments. Upon activation, the interaction of KRAS with these scaffold proteins would substantially reduce the diffusion coefficient and induce trapping in small membrane domains.

BRAF molecules from the cytosol transiently associate with activated KRAS via the RAS-binding domain, showing slow diffusion and transient trapping in discrete membrane zones (Figures 5C and 6D–6F). KRAS-BRAF complexes form larger signaling assemblies with RAF family members, scaffold proteins (KSR1, IQGAP1), and MEK, reducing diffusion coefficients. The CRD of BRAF and KSR1 interact with the PM via PS and KRAS, facilitating KRAS immobilization in small membrane zones.^35^^,^^36^^,^^37^^,^^38^^,^^49^ This aligns with our observations of small BRAF trapping zones (∼200π nm^2^), comparable to activated KRAS (Figure 5F).

SOS1 binds to inactive KRAS, enabling GDP release and GTP loading, which triggers SOS1 dissociation and KRAS conformational changes for BRAF interaction.^24^^,^^25^^,^^65^ While activated SOS1 showed slow diffusion rather than immobilization (Figures 5C, 6B, and S2B), its interaction with KRAS reduced KRAS diffusion and induced larger transient trapping zones (277π nm^2^) compared to activated KRAS and BRAF (Figure 5F).

PS depletion induced consistent alterations in KRAS trapping behavior, including reduced temporal fractions, shortened durations, and enlarged trapping zone sizes in both KRAS WT and oncogenic mutants (Figures 4D–4F). These coordinated changes suggest that the membrane environment, particularly PS, plays a critical role in the spatiotemporal regulation of KRAS dynamics (Figure 4A). Although KRAS nanoclusters were not directly assessed in this study, it remains plausible that they contribute to the observed diffusion characteristics and transient trapping of KRAS. In particular, PS depletion may impair nanocluster integrity, thereby altering KRAS membrane dynamics. In addition, PS depletion may impede BRAF recruitment to the PM by disrupting its interaction with PS via the CRD. This may reduce the frequency of KRAS-BRAF encounters at the PM. Moreover, even when such interactions occur, the diminished membrane anchoring of BRAF under PS-depleted conditions may destabilize the KRAS-BRAF complex, thereby limiting the formation of prolonged or spatially confined trapping events. These findings are consistent with the hypothesis that KRAS-BRAF interactions, and possibly the assembly of downstream signaling complexes, underlie the formation of spatially confined and/or long-duration trapping events.

The present study demonstrated that the biochemical properties specific to KRAS mutations can be directly correlated with the diffusional behaviors of KRAS. Before EGF stimulation, the temporal fraction of KRAS WT trapping was approximately 3.2%, which increased to a peak value of 8.1% at 2 min after EGF stimulation and subsequently decreased to 3.4% at 5 min (Figure 2F). KRAS trapping dynamics closely paralleled SOS1 recruitment to the PM (Figures 2F and 5B). The transient nature of KRAS trapping likely arises from the rapid nucleotide exchange and hydrolysis characteristic of KRAS WT. As KRAS is activated by SOS1, the temporal fraction of KRAS trapping—reflecting the cumulative duration of individual trapping events—should closely correlate with the number of SOS1 molecules recruited to the PM. Notably, while oncogenic KRAS mutants are generally regarded as predominantly locked in the active GTP-loaded state, their nucleotide-binding dynamics are more variable than previously anticipated. KRAS G13D exhibits distinct properties that enhance nucleotide exchange due to the destabilization of the nucleotide-binding pocket while retaining sensitivity to NF1-mediated GTP hydrolysis.^7^^,^^26^^,^^66^ Before EGF stimulation, the temporal fraction of KRAS G13D trapping was comparable to that of G12D and G12C (5.6%–6.1%) as a result of auto-activation via intrinsic nucleotide exchange, despite its NF1-mediated GTP hydrolytic activity (Figure 2F). Upon stimulation, the temporal fraction of KRAS G13D trapping sharply increased to 13.2% at 2 min and then decreased slightly to 11.7% at 5 min, paralleling SOS1 activity. In contrast, the temporal fractions of G12D and G12C trapping were initially similar (6.1% and 5.6%, respectively) before EGF stimulation but increased more gradually than G13D upon stimulation. This suggests that KRAS G12D and G12C mutants are less responsive to activation by SOS1 molecules than the G13D mutant. In comparison, even before stimulation, KRAS G12V showed a high temporal trapping fraction (8.0%; Figure 2F) and a small trapping size (Figure 3B), with no significant changes upon stimulation. These results are consistent with previous studies showing that the intrinsic and GAP-mediated GTP hydrolytic activities of KRAS G12V are significantly lower than those of KRAS WT and other mutants, resulting in higher retention of KRAS-GTP after serum starvation and less likely to be further activated by SOS1 molecules.^7^^,^^30^ Moreover, these results suggest that intrinsic GTP hydrolytic activity and sensitivity to GAPs, especially noncanonical GAPs such as RGS3, may differ among KRAS G12 mutants.^7^^,^^27^^,^^67^ This is further supported by the fact that the KRAS G12C inhibitor effectively traps nearly all KRAS G12C molecules in the inactive GDP-bound state (Figure S5E). In summary, the activity of oncogenic KRAS mutants varies and is dependent on upstream signaling. Importantly, accumulating clinical evidence indicates that KRAS mutations are neither biologically nor prognostically equivalent. Specific alleles, such as G12C, have been associated with poorer outcomes in patients with metastatic CRC.^20^^,^^21^^,^^22^^,^^23^ These clinical distinctions likely reflect intrinsic differences in the biochemical behavior of each mutant, including GTPase activity, responsiveness to GAPs, and upstream signaling inputs. Our single-molecule imaging results, which reveal distinct mutation-specific differences in KRAS diffusional dynamics, support the notion that each KRAS variant represents a unique biological entity. These findings underscore the importance of treating KRAS-mutant CRC not as a uniform group but as a heterogeneous set of subtypes with differential signaling properties and therapeutic vulnerabilities.

Single-molecule imaging enabled a highly sensitive evaluation of molecular-targeted drug efficacy by capturing changes in KRAS diffusional dynamics—temporal fraction, trapping duration, trapping zone size, and diffusion coefficient—all of which strongly correlated with KRAS activation status in a mutation-specific manner. Cetuximab treatment moderately reduced the temporal trapping fractions of oncogenic KRAS mutants except G12V (Figure 7A), increased the sizes of trapping zones (Figure 7B), and elevated diffusion coefficients (Figure S8A). These moderate changes in the trapping behavior of oncogenic KRAS mutants may correlate with a slight attenuation of their activity after cetuximab treatment, which fully deactivates KRAS WT. In particular, cetuximab hardly altered KRAS G12V trapping, as anticipated, given that KRAS G12V exhibits low GTP hydrolytic activity and does not rely on EGF stimulation for sustained activation. Notably, it has been suggested that KRAS G13D mutation in patients with metastatic CRC may respond more favorably to cetuximab treatment than other oncogenic KRAS mutations.^68^^,^^69^ Our single-molecule imaging showed that cetuximab’s effect on KRAS G13D was similar to its effect on G12D and G12C (Figure 7D), aligning with their intrinsic activities. This result is also consistent with a previous study showing that the sensitivity of EGFR inhibitors in suppressing KRAS mutant activity is not directly dependent on the GTP hydrolysis of these mutants by NF1 (GAP) but rather on their binding affinity to NF1.^70^ These results revealed that single-molecule imaging is an excellent approach to precisely evaluate the efficacy of cetuximab.

It has been reported that modulating upstream signaling through receptor tyrosine kinases can be effective, as the nucleotide-bound state of KRAS G12C can be shifted toward the GDP-bound state, thereby enhancing the efficacy of KRAS G12C inhibitors.^11^ The primary effect of combining upstream blockade with KRAS inhibitor monotherapy comes from preventing feedback reactivation through other wild-type RAS proteins, including HRAS or NRAS.^19^^,^^71^ Indeed, pull-down assays showed that GTP-bound KRAS G12D and G12C were nearly eliminated by KRAS inhibitor treatment alone (Figure S5E). However, single-molecule imaging revealed that transient trapping events of KRAS G12D and G12C persisted under KRAS inhibitor monotherapy, occurring with an average temporal fraction similar to that observed with cetuximab treatment in KRAS G12D (Figure 8A) and with sizes of trapping zones similar to those observed with cetuximab in KRAS G12D and G12C (Figure 8B). In other words, single-molecule imaging can sensitively detect low KRAS activities. Notably, combined therapies exhibited additive effects on oncogenic KRAS signaling, as evidenced by a reduced temporal fraction of trapped KRAS G12D (Figure 8A), and a shift of KRAS G12C trapping zone size toward larger zones (Figure 8B). These results indicate that single-molecule imaging offers a highly sensitive approach for detecting subtle changes in KRAS signaling induced by various molecular-targeted therapies and combination therapies.

In summary, single-molecule imaging revealed that membrane zones of transient KRAS trapping act as critical signaling platforms. These zones enable precise quantification of the activation states of both wild-type and mutant KRAS by analyzing their diffusion behaviors and temporal trapping events. The methodologies employed in this study hold significant promise for evaluating mutation-specific signaling dynamics of oncogenic KRAS molecules and for assessing the therapeutic efficacy of molecular-targeted drugs from a spatiotemporal perspective in living cells. This approach is expected to advance our understanding of the mechanisms underlying oncogenic KRAS signaling and contribute to the development of more effective therapeutic strategies.

### Limitations of the study

Our results demonstrate that temporal trapping of single KRAS molecules—both WT and oncogenic mutants—detected by TIRFM serves as a sensitive indicator of activity levels and therapeutic efficacy, often surpassing conventional western blot analysis. However, several limitations should be noted. First, expression of fluorophore-labeled KRAS must be kept sufficiently low (<1 molecule/μm^2^) to allow accurate single-molecule tracking, necessitating use of tightly controlled expression systems such as the pOsTet15T3 vector employed in this study. Second, while single-molecule imaging is a powerful tool for unraveling stochastic and rare events, we require a TIRF microscope to do so. Third, our conclusions are primarily based on experiments conducted in SW48 cells. Given the context-dependent nature of KRAS signaling, validation in additional CRC cell lines with distinct EGFR/KRAS pathway activity will be critical for broader generalization. Fourth, although our study includes data on the effects of KRAS-targeted inhibitors such as adagrasib, comparative analyses using other clinically approved KRAS G12C inhibitors (e.g., sotorasib) may offer further insight into the generalizability of our approach. Lastly, while our imaging approach successfully links KRAS diffusional behavior to its activation state, it does not directly address downstream phenotypic consequences, such as MAPK signaling or cellular proliferation. These processes are governed by complex signaling networks over extended timescales and require additional biological models and experimental paradigms beyond the scope of this study. Future work integrating single-molecule imaging with functional assays will be essential to elucidate the pathological implications of KRAS diffusional dynamics in living cells.

## Resource availability

### Lead contact

Request for further information and resources and reagents should be directed to and will be fulfilled by the lead contact, Kenichi G. N. Suzuki (suzuki.kenichi.b7@f.gifu-u.ac.jpand kesuzuk@ncc.go.jp).

### Materials availability

Plasmids generated in this study are available from the lead contact upon request.

### Data and code availability

• This paper analyzes existing, publicly available data, which are listed in the key resources table.
• No original code was reported in this paper.
• Any additional information required to reanalyze the data reported in this paper is available from the lead contact upon request.

## Acknowledgments

We thank Prof. Yoshihiro Miwa for the gift of a cDNA plasmid encoding a pOsTet15T3 vector. We thank Shinobu Kawaguchi for constructing the cDNA plasmids. We also thank Rinshi S. Kasai (National Cancer Center) for setting up the single-molecule tracking station. This work was supported in part by Grants-in-Aid for Scientific Research from the 10.13039/501100001691Japan Society for the Promotion of Science Kiban B to K.G.N.S. (24K01974, 21H02424), a Grant-in-Aid for Challenging Exploratory Research from JSPS to K.G.N.S. (24K21944), JSPS Core-to-core Program to K.G.N.S. (JPJSCCA202000007), Grant-in-Aid for Innovative Areas from the 10.13039/501100001700Ministry of Education, Culture, Sports, Science and Technology of the Japanese government (MEXT) to K.G.N.S. (18H04671), Japan Science and Technology Agency (JST) grant in the program of the 10.13039/501100003382Core Research for Evolutional Science and Technology in the field of “Extracellular Fine Particles” to K.G.N.S. (JPMJCR18H2) and “Cell Control” to K.G.N.S. (JPMJCR24B3), the National Cancer Center Research and Development Fund to K.G.N.S. (2023-A-03), the 10.13039/100009619Japan Agency for Medical Research and Development to K.G.N.S. (JP21km0908001, JP24ym0126134), the 10.13039/100007449Takeda Science Foundation to K.G.N.S., the 10.13039/100008732Uehara Memorial Foundation to K.G.N.S., the 10.13039/100009578Mizutani Foundation for Glycoscience to K.G.N.S., Grants-in-Aid for Scientific Research from JSPS Kiban C to R.Y. (25K10533), and COMIT Collaborative Research 2023 to R.Y.

## Author contributions

Conceptualization, R.Y., N.M., and K.G.N.S.; methodology, R.Y., T.T., and K.G.N.S.; formal analysis, R.Y. and K.G.N.S.; investigation, R.Y., T.M., K.M.H., and R.K.; writing—original draft, R.Y.; writing—review & editing, R.Y. and K.G.N.S.; supervision, N.M. and K.G.N.S.; funding acquisition, R.Y. and K.G.N.S.

## Declaration of interests

The authors declare no competing interests.

## Declaration of generative AI and AI-assisted technologies in the writing process

During the preparation of this work, the authors used ChatGPT4o in order to improve readability and language. After using this tool, the authors reviewed and edited the content as needed and take full responsibility for the content of the published article.

## STAR★Methods

### Key resources table

**Table:** 

REAGENT or RESOURCE	SOURCE	IDENTIFIER
**Antibodies**		

Ras Mouse mAb	Cell Signaling	Cat# 8832
Goat Anti-Mouse IgG Antibody, HRP conjugate	Sigma-Aldrich	Cat# 12-349; RRID: AB_390192

**Chemicals, peptides, and recombinant proteins**		

Cetuximab	Merck	CAS: 205923–56-4
Adagrasib (MRTX849)	Selleck	Cat# S8884
MRTX1133	Selleck	Cat# E1051
BAY-293	Selleck	Cat# S8826
RMC-4550	Selleck	Cat# S8718
Human EGF	PEPROTECH	Cat# AF-100-15-100ug
Glutathione Resin	Cell Signaling	Cat# 11523
GST-Raf1-RBD	Cell Signaling	Cat# 8784
HaloTag TMR Ligand	Promega	Cat# G8251
HaloTag SaraFluor™ 650B (SF650B) Ligand	GORYO CHEMICAL	Cat# A201-01

**Critical commercial assays**		

Q5 site-directed mutagenesis kit	New England Biolabs	Cat# E0554S
Cell Line Optimization 4D-Nucleofector X Kit	Lonza	Cat# V4XC-9064
Active Ras Detection kit	Cell Signaling	Cat# 8821

**Experimental models: Cell lines**		

SW48	ATCC	Cat# CCL-231
Caco-2	ATCC	Cat# HTB-37

**Recombinant DNA**		

KRAS	Gift from Tomohiko Taguchi	N/A
pEGFPC1-tdStayGold-KRAS WT	This work	N/A
pEGFPC1-tdStayGold-KRAS G13D	This work	N/A
pEGFPC1-tdStayGold-KRAS G12D	This work	N/A
pEGFPC1-tdStayGold-KRAS G12C	This work	N/A
pEGFPC1-tdStayGold-KRAS G12V	This work	N/A
pEGFPC1-tdStayGold-KRAS S17N	This work	N/A
pOsTet15T3-tdStayGold-KRAS WT	This work	N/A
pOsTet15T3-tdStayGold-KRAS G13D	This work	N/A
pOsTet15T3-tdStayGold-KRAS G12D	This work	N/A
pOsTet15T3-tdStayGold-KRAS G12C	This work	N/A
pOsTet15T3-tdStayGold-KRAS G12V	This work	N/A
pOsTet15T3-tdStayGold-KRAS S17N	This work	N/A
pDonor-255-SOS1	Addgene	Cat#70601; RRID:Addgene_70601
pEGFPC1-tdStayGold-SOS1	This work	N/A
pEGFPC1-HaloTag7-SOS1	This work	N/A
pcDNA3.1-Hygro-BRAF	Addgene	Cat#40775; RRID:Addgene_40775
pEGFPC1-tdStayGold-BRAF	This work	N/A
pEGFPC1-HaloTag7-BRAF	This work	N/A
pcDNA3-mCherry-yPSD1	This work	N/A
pEGFPC2-HaloTag7-evectin2 (2xPH)	This work	N/A
pEGFPN1-mStayGold-LDLR-TM	This work	N/A
pEGFPC1 vector	Clontech	Cat# 6084-1
pOsTet15T3 vector	Gift from Yoshihiro Miwa, University of Tsukuba	N/A
tdStayGold (c4)/pBS Coupler	RIKEN BRC	Cat# RDB19610
mStayGold (c4)/pUC-GW-Kan	Synthesized by Azenta Life Sciences according to the sequence in Ando et al.^72^	N/A
pFN21A-HaloTag7	Promega	Cat# G2821

**Software and algorithms**		

4-D Nucleofector	Lonza	Cat# AAF-1001B and Cat# AAF-1001X
Chemiluminescence Imaging System	Vilber Lourmat	Cat# FUSION-SOLO.7S
OriginPro 2020	OriginLab	RRID:SCR_014212
ImageJ Fiji	NIH	RRID:SCR_002285

**Other**		

SE Cell Line Solution	Lonza	Cat# PBC1-02250
Lysis/Binding/Wash Buffer	Cell Signaling	Cat# 11524
Protease Inhibitor Cocktail Set III	Millipore	Cat# 539134
Bolt 4 to 12%, Bis-Tris Plus WedgeWell Gel	Invitrogen	Cat# NW04125
PVDF membrane	Millipore	Cat# IPVH00010
Blocking One	nacalai tesque	Cat# 03953-95
Glass-base dish	Iwaki	Cat# 3911-035

### Experimental model and study participant details

#### Cell lines and cell culture

The SW48 cell line (CCL-231) and the Caco-2 cell line (HTB-37) were obtained from the American Type Culture Collection (ATCC). Both cell lines are colorectal adenocarcinoma and harbor the wild-type *RAS* gene. SW48 cells were cultured in RPMI-1640 medium supplemented with 10 % fetal bovine serum (FBS), 100 U/ml penicillin, 100 μg/ml streptomycin, and 2 mM L-glutamine, under a humidified atmosphere containing 5% CO_2_ at 37°C. Caco-2 cells were cultured in Minimum Essential Medium (EMEM) supplemented with 10% FBS, 100 U/mL penicillin, 100 μg/ml streptomycin, and 2 mM L-glutamine, maintained under identical conditions.

### Method details

#### cDNA construction, mutagenesis, and transfection

The cDNAs encoding human KRAS (GenBank: NM_004985.5), SOS1 (GenBank: NM_005633.4), and BRAF (GenBank: NM_004333.6) were cloned into the pEGFPC1 vector (Clontech) and fused to tdStayGold (c4)^73^ (RIKEN BRC) with a GGGGS ×3 linker or fused to HaloTag7 (Promega) with an SG ×3 linker at the N-terminus. The cDNA encoding the transmembrane domain of the LDL receptor (LDLR-TM)^28^ (a negative control molecule) was cloned into the pEGFPN1 vector (Clontech) and fused to mStayGold (c4)^72^ (synthesized by Azenta Life Science according to the sequence in Ando et al.^72^) at the N-terminus. Mutagenesis of the KRAS DNA sequences for the oncogenic mutations G13D, G12D, G12C, and G12V−frequent in colorectal cancer−along with the dominant-negative mutant KRAS S17N, was performed using pEGFPC1-tdStayGold-KRAS WT as a template. This was performed with the Q5 Site-Directed Mutagenesis Kit (New England Biolabs), following the manufacturer’s protocol. Additionally, each cDNA plasmid encoding tdStayGold-KRAS WT and its mutants was subcloned into the EBV-based episomal vector pOsTet15T3, which incorporates tetracycline-regulated expression units, the transactivator (rtTA2-M2), and the TetO sequence (a Tet-on vector).^28^ To prevent overexpression of tdStayGold-KRAS for single-molecule observations, doxycycline induction was omitted, instead relying on basal leaky expression. All newly constructed plasmids were verified by DNA sequencing. SW48 and Caco-2 cells were transfected with these cDNA plasmids using the 4-D Nucleofector (Lonza), following the manufacturer’s instructions. As no optimized protocol for the SW48 cell line was provided by Lonza, the transfection program CY-100, using SE Cell Line Solution, using an optimization kit (Lonza), was determined to be the most effective.

#### RAS-GTP pull-down and western blotting

SW48 cells were transfected with pEGFPC1-tdStayGold-KRAS cDNA plasmids and cultured for 36 h before biochemical assays. When applicable, molecular-targeted drugs were administered at final concentrations of 100 nM for cetuximab (an inhibitor of EGFR monoclonal humanized antibody interacting with the extracellular binding site of EGFR to block ligand stimulation), 100 nM for adagrasib (KRAS G12C inhibitor), 30 nM for MRTX1133 (KRAS G12D inhibitor), 12 h before conducting the assays. After 1 h of serum starvation in serum-free medium, cells were stimulated with 10 nM EGF (PEPROTECH). Drug concentrations were maintained consistently throughout the procedures. RAS-GTP pull-down and Western blot were performed using the Active Ras Detection Kit (Cell Signaling), according to the manufacturer's protocol. Briefly, at the designated time points before and after EGF stimulation, cells were placed on ice, washed with ice-cold PBS, and lysed using ice-cold Lysis/Binding/Wash Buffer supplemented with a Protease Inhibitor Cocktail Set III (Millipore). The lysates were clarified by centrifugation and partially aliquoted for quantification of total RAS proteins (including both active and inactive forms), while the remaining was used for RAS-GTP pull-down, employing glutathione resin and GST-Raf1-RBD. The samples were resolved by SDS-PAGE on Bolt 4% to 12% Bis-Tris Plus WedgeWell Gel (Invitrogen) and subsequently transferred to PVDF membranes (Millipore) via electrophoresis at 20V for 1 h. After transfer, the membranes were blocked for 30 min at room temperature and subsequently incubated overnight at 4°C with Ras Mouse mAb (Cell Signaling) in a blocking buffer (Nacalai Tesque). Following the incubation, the membranes were washed with TBS containing 0.1% Tween-20 and probed with goat anti-mouse IgG antibody conjugated to HRP (Sigma-Aldrich). Protein bands were visualized and quantified using the Chemiluminescence Imaging System (Vilber Lourmat). Since the lysate volume used for the pull-down assay was 8-fold greater than that containing the total RAS protein, the percentage of activated RAS molecules was calculated by dividing the band intensity of KRAS obtained through the pull-down by the band intensity of total KRAS, and subsequently dividing the result by 8. Data are presented as mean ± SEM, with the individual data points from more than 3 independent experiments overlaid.

#### Single fluorescent-molecule imaging

All live-cell microscopic observations were performed at 37°C. For single-molecule imaging, cells were transfected with pOsTet15T3-tdStayGold-KRAS, pEGFPC1-tdStayGold-SOS1, or pEGFPC1-tdStayGold-BRAF cDNA plasmids, sparsely seeded in a glass-base dish (4×10^3^ cells on a 12-mm diameter glass window, 0.15-mm-thick glass; Iwaki), and cultured for 36 h before each observation. Where applicable, molecular-targeted drugs were administered at final concentrations of 100 nM for cetuximab, 100 nM for adagrasib, 30 nM for MRTX1133, 5 μM for BAY-293 (SOS1 inhibitor), and 1 μM for RMC-4550 (SHP2 inhibitor), 12 h before imaging. After 1 h of serum starvation in phenol red-free, serum-free medium, single molecules on the basal side of the cell PM, facing the coverslip, were visualized at video rate (33-ms resolution) at designated time points before and after EGF stimulation. Single-molecule imaging was performed using a home-built, objective-lens-type total internal reflection fluorescence microscopy (TIRFM) based on a Nikon Ti inverted microscope (100 × 1.49 NA oil objective) equipped with a qCMOS camera (ORCA-Quest; Hamamatsu Photonics). The number densities of the fluorescent KRAS, SOS1, and BRAF molecules on the PM were approximately 0.3 copies/μm^2^. The precision of the position determinations for single stationary fluorescent probes was estimated from the standard deviations of probe coordinates fixed on coverslips. For tdStayGold and TMR probes recorded at video rate, the localization precisions of single molecules were 14.0±1.8 nm and 18.7±1.6 nm, respectively. The photobleaching lifetimes of tdStayGold and TMR probes immobilized on glass were estimated to be 10.4±0.6 s and 8.1±0.7 s, respectively, by monitoring the durations until single-step photobleaching and fitting the data to a single-exponential decay curve.^30^^,^^55^^,^^62^ All measurements of signal intensities for individual fluorescent spots were restricted to the central region (20 μm in diameter) of the illuminated area, where non-uniformities in laser illumination were minimal.

Trajectories longer than 10 frames were subjected to quantitative analysis. Care was taken to use cells with comparable molecular expression levels across all experiments. For EGF stimulation, a cell culture medium containing 20 nM EGF was added to the cells to achieve a final concentration of 10 nM (0.5 mL of 20 nM EGF in the medium was added to a glass-base dish containing 0.5 mL medium). Molecular-targeted drug concentrations were maintained throughout all experimental procedures. For dual-color simultaneous observation of single KRAS and SOS1, BRAF, and TM molecules, SW48 cells were co-transfected with cDNA plasmids in the following combinations.

• pOsTet15T3-tdStayGold-KRAS and pEGFPC1-HaloTag7-SOS1
• pOsTet15T3-tdStayGold-KRAS and pEGFPC1-HaloTag7-BRAF
• pEGFPC1-tdStayGold-SOS1 and pEGFPC1-HaloTag7-BRAF
• pEGFPC1-HaloTag7-SOS1 and pEGFPN1-mStayGold-LDLR-TM

To achieve covalent binding of TMR to HaloTag7, cells were incubated in a medium containing 10 nM HaloTag TMR ligand (Promega) for 30 min at 37°C, followed by three washes with medium to remove unbound ligand. Any residual unbound ligand in the cytoplasm was eliminated by incubating the cells in the medium for an additional 30 min, followed by three washes with fresh medium. For simultaneous two-color single-molecule imaging, images were recorded at 37°C at video rate using an objective-lens-type TIRF microscope based on a Nikon Ti inverted microscope (100x 1.49 NA oil objective) equipped with two qCMOS cameras (ORCE-Quest, Hamamatsu Photonics). The superimposition of images in different colors obtained from the two separate cameras was performed as previously described.^40^^,^^41^^,^^62^ Briefly, to determine the individual characteristics of each imaging path, a predefined mask comprising a lattice of optical apertures with 1 μm diameter and 2.5 μm spacing was simultaneously imaged through both paths. The resulting images were then subjected to spatial correction. In two-color, simultaneous single fluorescent-molecule tracking experiments employing the tdStayGold/TMR pair, the spatial distance between the two molecules was directly measured from their coordinates (*x*, *y* positions), determined simultaneously and independently for each color channel.^28^^,^^40^^,^^41^^,^^59^^,^^74^^,^^75^ Colocalization of two fluorescent molecules in simultaneous two-color single-molecule observations was defined as an event where the fluorescent spots representing the two molecules were localized within 240 nm of each other, as previously described.^28^^,^^40^^,^^41^^,^^59^^,^^74^^,^^75^ Based on the accuracies determined in this and previous studies, we found that, for truly associated molecules, the probability of scoring the two molecules as associated increases to 99% using the criterion that the molecules lie within 240 nm of each other.^28^^,^^40^^,^^41^^,^^59^^,^^74^^,^^75^ Therefore, we used this criterion as the definition of colocalization in simultaneous two-color single-molecule observations.

#### Observation of single-molecule recruitment of SOS1 and BRAF

To assess the temporal dynamics in the recruitment of single molecules of SOS1 and BRAF to the PM, these molecules were monitored within the same cells by TIRFM at specific time intervals, both before and after EGF stimulation. The fluorescence intensities of all detected spots within trajectories over 10 frames—after subtracting the mean background fluorescence intensity—were summed per each cell. Molecules persisting on the PM for over 10 frames were categorized as recruited to the PM, in contrast to those transiently colliding with the PM during cytoplasmic diffusion. For each cell, the fluorescence intensity ratio at each time point was calculated relative to the intensity observed 1 min after EGF stimulation. The data represent the time course of the mean fluorescence intensity ratio ± SEM, based on observations from more than 12 cells across at least three independent experiments.

#### Diffusion coefficient analysis

The methods for generating the plots of mean square displacement (MSD) versus time and calculating diffusion coefficients from the slopes of these MSD plots have been previously described.^54^^,^^56^^,^^62^ Briefly, the single-molecule MSD for a given time interval, *Δt*, *i.e.*, *Δr(Δt)*^*2*^, is defined as follows. For a single-molecule trajectory consisting of *N-determined* coordinates (*x-*, *y-*positions) in a two-dimensional plane, all [*N*-*n*+1] partial trajectories of *n* consecutive positions (n ≤ N) were extracted. The MSD (*N*, *n*) was then calculated by averaging the square displacements over *n* steps for all [*N*-*n*+1] partial trajectories, and by varying *n*, the plot of MSD (*Δt*_*n*_ = *nδt*) versus *nδt* (*δt* = the duration of each image frame) was obtained. Thus, the MSD for each time interval was calculated according to the following formula:

MSD(*Δt*_*n*_) = MSD(*nΔt*)= MSD_x_(MSD_x_(*nΔt*) + MSD_y_(*nΔt*)

$$=\frac{1}{N-n+1}\;\sum_{j=1}^{N-n+1}\{{\left[x\left(j\delta t+n\delta t\right)-x\left(j\delta t\right)\right]}^{2}+{\left[y\left(jdt+ndt\right)-x\left(jdt\right)\right]}^{2}\}$$

where *δt* is the frame time, *x*(*jδt +nδt*) and *y*(*jδt +nδt*) describe the particle position following a time interval, *δt*_n_ = *nδt*, after starting at position (*x*(*jδt*), *y*(*jδt*)), *N* is the total number of frames in the recording sequence, and *n* and *j* are positive integers (*n* determines the time increment). The diffusion coefficient of a particle in the time scale of 200 ms (D_200ms_) was obtained by linearly fitting its single-molecule MSD-Δ*t* plot at the 167 ms, 200 ms, and 233 ms timepoints (the slope divided by 4 gives the diffusion coefficient). We evaluated D_200ms_ using 330-ms trajectories. Trajectories with D_200ms_ values below 0.009 μm^2^/s were classified as immobile mode, a threshold determined by the 95th percentile value of molecules leaking from dead cells and immobilized on glass. The histograms of diffusion coefficients consist of more than 1000 trajectories.

#### Detection and quantitative analysis of transient molecular trapping

Trajectories longer than 10 frames were analyzed to detect transient molecular trapping events and to estimate their duration, frequency, and zone size of trapping, using the method developed by Sahl et al.,^39^ with our previous modification.^28^^,^^41^^,^^59^ In this study, transient trapping was defined as events where molecules were confined for 5 or more frames within a region of 100 nm diameter. The size of the area occupied by a molecule during trapping was quantified as the standard deviation (SD) of the distances between the mean position during the trapping period and the individual positions determined at each frame. This measurement was corrected for localization precision (position determination of single molecules) via 2D Gaussian fitting of the coordinates during the trapping period. The temporal fraction of trapped molecules was calculated as the ratio of the total trapping time to the total trajectory duration for each cell. The trapping frequency was quantified as the number of trapping events per unit of time over the total trajectory. The temporal fraction and frequency of trapped molecules are presented in box-and-whisker plots, displaying the minimum, maximum, sample median, sample mean (circle), first and third quartiles, and whiskers extending to a maximum of 1.5× interquartile range beyond the box. Corresponding data points were overlaid for 40 cells without EGF stimulation and 20 cells for each time point (2, 3.5, 5 min) after EGF stimulation, with a total of 60 EGF-stimulated cells analyzed across more than 3 independent experiments. The distributions of the duration and zone size of individual trapping events are shown as a sum of all cells for each experimental condition, such as KRAS mutation or treatment with molecular-targeted drugs. The size distribution of individual trapping zones is presented as both violin plots and box-and-whisker plots, indicating the sample median, sample mean (circle), first and third quartiles, and whiskers extending to a maximum of 1.5× interquartile range beyond the box.

#### Quantitative analysis of KRAS transient trapping under PS depletion

To assess PS depletion in the PM of cells expressing PSD, a PS-specific probe was employed. This probe consisted of two tandem PH domains of evectin-2 fused to HaloTag7 (HaloTag7-evectin2 [2×PH]).^48^ SW48 cells were co-transfected with pcDNA3-mCherry-yPSD1 and pEGFPC2-HaloTag7-evectin2 (2×PH) plasmids. For covalent labeling of HaloTag7 with SF650B, cells were incubated with 50 nM SF650B-Halo ligand (GORYO CHEMICAL) in culture medium for 30 min at 37°C, followed by three washes with fresh medium to eliminate unbound ligand. Residual cytoplasmic ligand was removed by an additional 30-min incubation in fresh medium, followed again by three washes. Fluorescence images of SF650B-Halo7-evectin2 (2xPH) and mCherry-PSD were acquired under conditions with or without PSD expression. The binding of the PS probe to the inner leaflet of the PM was visualized at the single-molecule level using TIRFM. The number of fluorescent spots corresponding to recruited PS probe molecules was normalized to both the total expression level of the probe and the area of observation. Probe expression levels were estimated by measuring whole-cell fluorescence intensity via epi-fluorescence microscopy (achieved by switching from TIRF to vertical illumination). Cells expressing PSD exhibited a marked reduction in the normalized number of PS probe spots relative to control cells, indicating effective PS depletion. The normalized spot counts of recruited PS probe molecules are depicted as box-and-whisker plots, representing the minimum, maximum, sample median, sample mean (circle), first and third quartiles, and whiskers extending up to 1.5 times the interquartile range beyond the box.

To investigate KRAS dynamics under PS-depleted conditions, SW48 cells were co-transfected with pcDNA3-mCherry-yPSD1 and pOsTet15T3-tdStayGold-KRAS plasmids. The transient trapping behavior of KRAS was subsequently assessed in cells expressing PSD using single-molecule imaging.

#### Colocalization analysis of SOS1 with BRAF, KRAS S17N, or TM using simultaneous dual-color single-molecule imaging

To assess the colocalization of SOS1 with KRAS S17N or BRAF on the PM, simultaneous dual-color single-molecule imaging was conducted in SW48 cells after EGF stimulation. Cells were co-transfected with appropriate combinations of cDNA plasmids encoding tdStayGold- or HaloTag7-tagged SOS1, KRAS S17N, BRAF, and TM (negative control) using the 4-D Nucleofector (Lonza), following the manufacturer’s instructions, as described previously.^62^ After transfecting the cells with cDNA plasmids, cells were seeded to a glass base dish and cultured for 36 h before the microscopic observations. HaloTag7-fusion proteins were labeled with 10 nM TMR ligand. Dual-color single-molecule images were acquired simultaneously for both fluorophores at a video rate, as described above.

To quantitatively evaluate the colocalization of single molecules of SOS1 with BRAF, KRAS S17N, or TM in the cell PM, the following analysis was performed as described previously.^52^^,^^53^ A region of interest (ROI) larger than 10 μm in diameter was selected for each field of view. For each video frame, the x–y coordinates of all fluorescent spots in both channels were determined as previously reported,^28^^,^^40^^,^^41^ and detailed in the “single fluorescent-molecule imaging” section. All pairwise distances between molecules of different colors in the ROI were measured. This process was repeated across all frames, and the number of molecular pairs was plotted as a function of inter-molecular distance, normalized to the ROI area to yield a distance-based density distribution. The colocalization index was defined as the ratio of molecular pairs within 0–50 nm to those within 400–500 nm. Colocalization indices are presented as box-and-whisker plots, displaying the minimum, maximum, sample median, sample mean (circle), first and third quartiles, and whiskers extending up to 1.5 times the interquartile range beyond the box.

### Quantification and statistical analysis

Statistical analyses and graph generation were performed using OriginPro (OriginLab). The data were statistically evaluated using the Mann-Whitney U test or Welch’s t-test, as described in the respective figure legends. ∗, ∗∗ and ∗∗∗ indicate *p* < 0.05, *p* < 0.01 and *p* < 0.001, respectively. A p-value of less than 0.05 was considered statistically significant. For analyses involving multiple comparisons, significance thresholds were adjusted using the Bonferroni correction. The corrected significance levels are shown in the legend of Figure 8.

## Supplemental information

Document S1. Figures S1–S8

Data S1. Source data for western blotting

Table S1. Summary table containing all statistical data, related to Figures 1, 2, 3, 4, 5, 7, 8, and S1–S8
