# Supplementary material for: Single-molecule imaging quantifies oncogenic KRAS dynamics for enhanced accuracy of therapeutic efficacy assessment
Source: iScience. 2025 Aug 14;28(9):113374. doi: 10.1016/j.isci.2025.113374 (PMC12424429; doi:10.1016/j.isci.2025.113374)
Supplement: Document S1. Figures S1–S8 [file mmc1.pdf]

iScience, Volume *28*

Supplemental information

Single-molecule imaging quantifies oncogenic KRAS dynamics for enhanced accuracy of therapeutic efficacy assessment

Ryoma Yokoi, Toshiki Mori, Koichiro M. Hirosawa, Ryojiro Kondo, Tomohiko Taguchi, Nobuhisa Matsuhashi, and Kenichi G.N. Suzuki

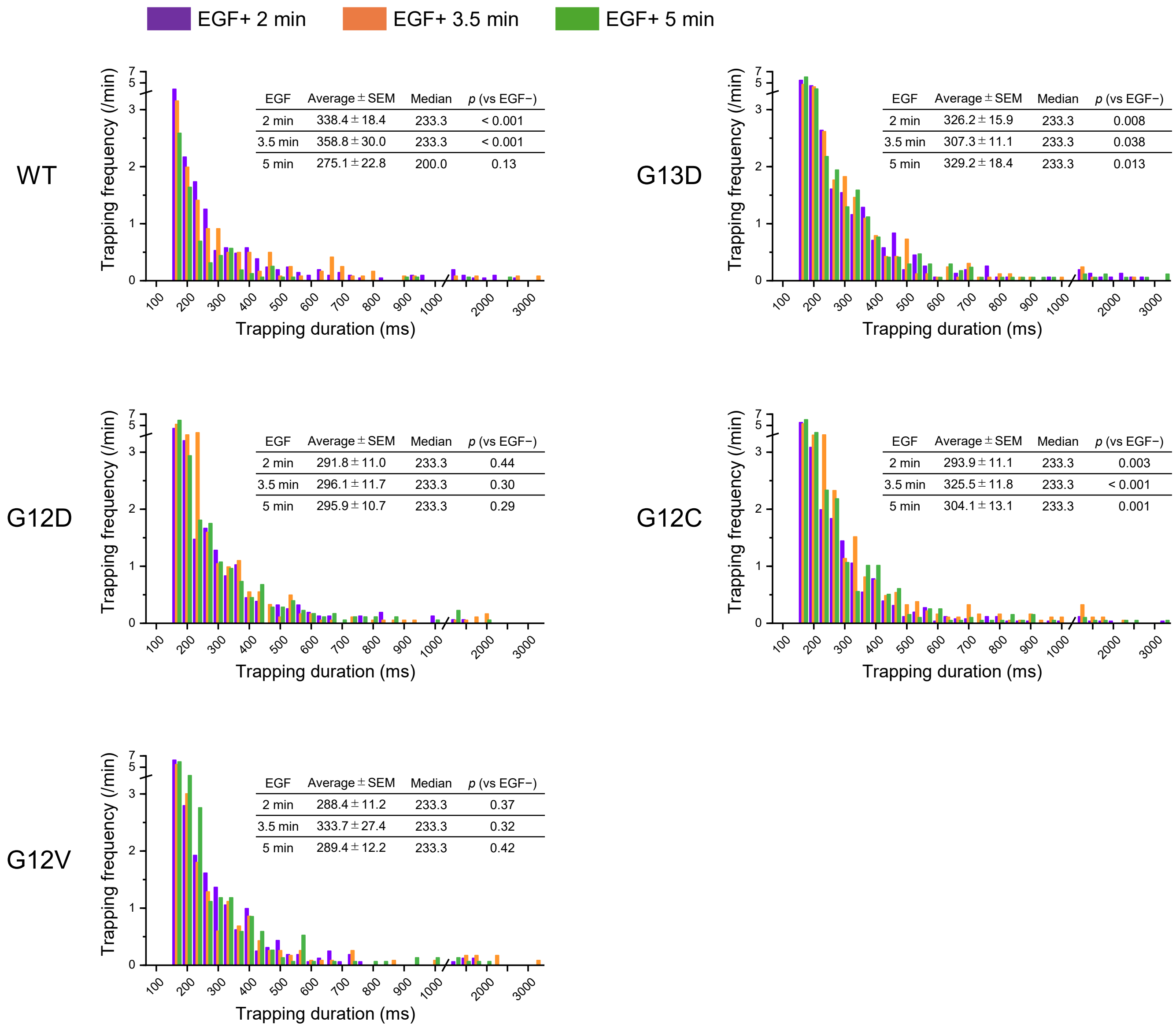

Figure S1. Time course of distributions of individual KRAS trapping durations 2, 3.5, and 5 min after EGF stimulation, related to Figure 3

The time course of trapping duration distributions for KRAS WT and oncogenic mutants was analyzed after EGF stimulation in SW48 cells. Data after EGF stimulation were statistically compared with data before EGF stimulation using Welch's t-test.

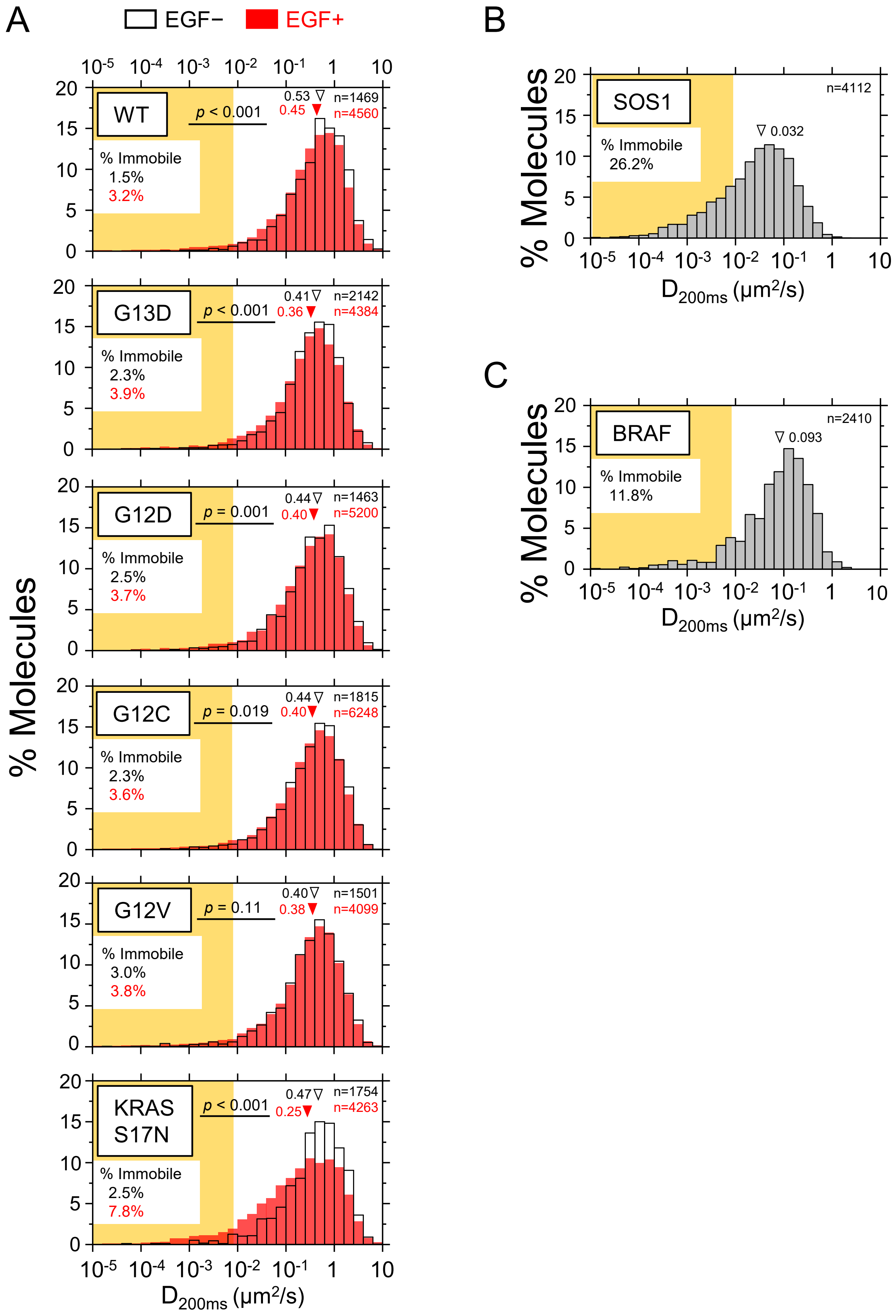

Figure S2. Distributions of diffusion coefficients in the time window of 200 ms (D_{200ms}) and the immobile fractions of individual KRAS, SOS1, and BRAF molecules

Histograms show the distributions of D_{200ms} for KRAS WT, oncogenic mutants, and the dominant-negative KRAS S17N both before and after EGF stimulation (A), as well as for SOS1 (B) and BRAF (C) after stimulation, derived from over 1000 trajectories in SW48 cells. The number of trajectories analyzed and immobile fractions are indicated at the top-right and left corners, respectively. Median D_{200ms} values are shown next to the arrowheads. Statistical analyses were performed using the Mann-Whitney U test.

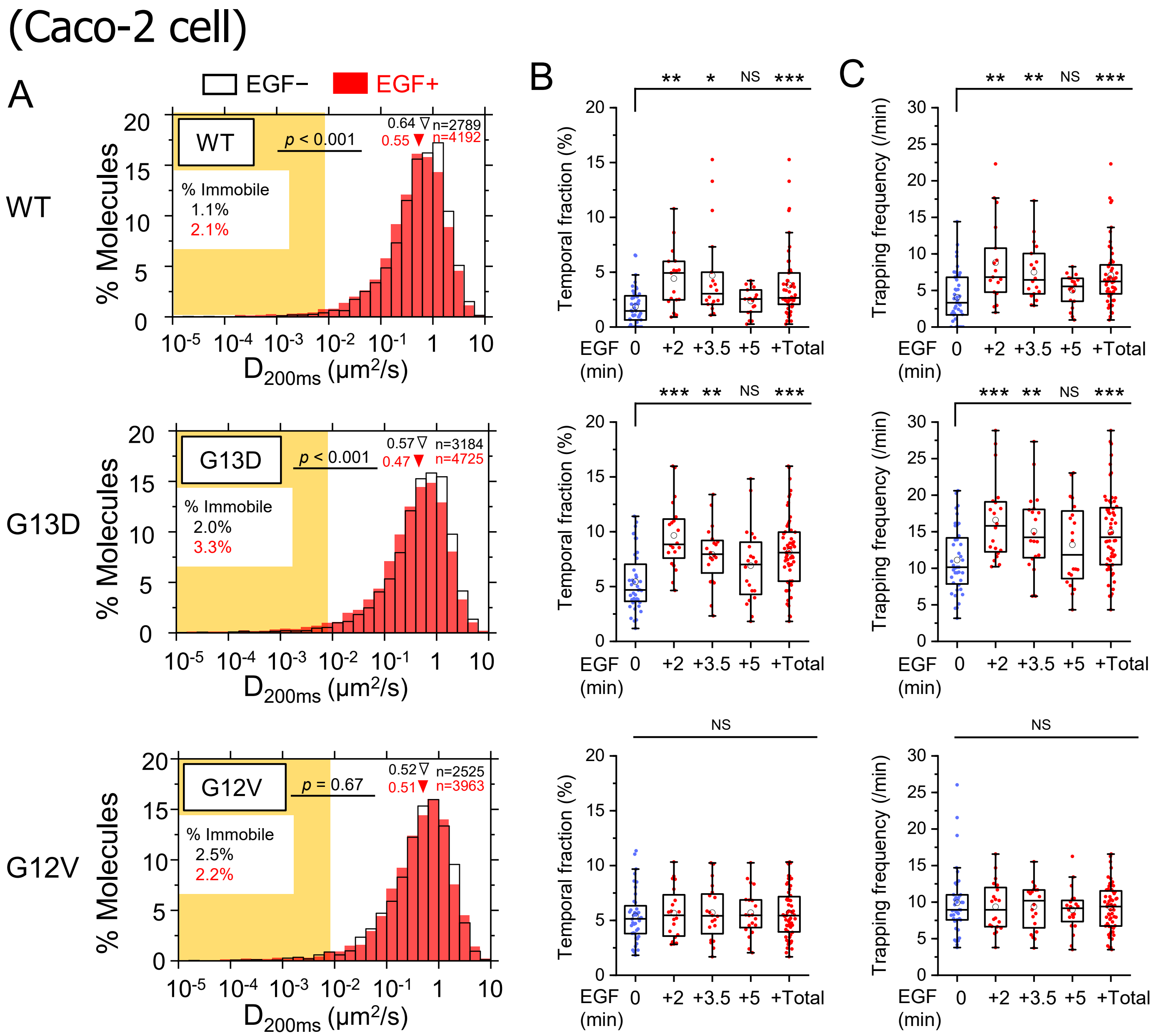

Figure S3. Distributions of diffusion coefficients (D_{200ms}), temporal trapping fractions, and trapping frequencies for KRAS WT and mutants in Caco-2 cells

(A) Histograms show the distributions of D_{200ms} for KRAS WT and oncogenic mutants before and after EGF stimulation, derived from over 1000 trajectories in Caco-2 cells. The number of trajectories analyzed and immobile fractions are indicated at the top-right and left corners, respectively. Median D_{200ms} values are shown next to the arrowheads. Data after EGF stimulation were statistically compared with data before EGF stimulation using the Mann-Whitney U test.

(B and C) Time course of trapping fractions (B) and trapping frequencies (C) for KRAS WT and oncogenic mutants 2, 3.5, and 5 min after EGF stimulation in Caco-2 cells (+Total includes all data 2, 3.5, and 5 min after stimulation). Data are presented in box-and-whisker plots, displaying the minimum, maximum, sample median, sample mean (circle), first and third quartiles, and whiskers extending to a maximum of 1.5× interquartile range beyond the box. Data after EGF stimulation were statistically compared with data before EGF stimulation using Welch's t-test. *$p < 0.05$, **$p < 0.01$ and ***$p < 0.001$.

(Caco-2 cell)

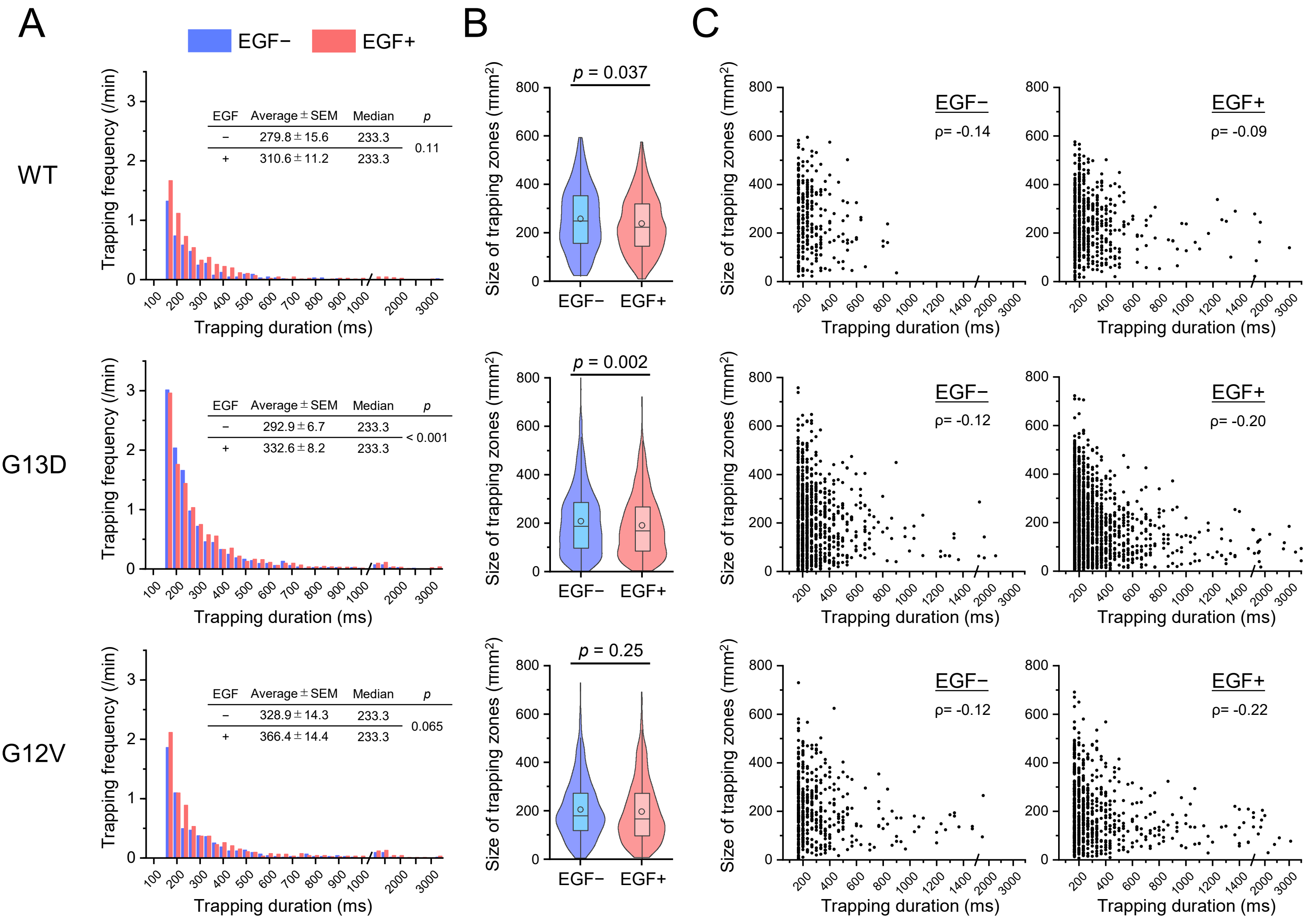

Figure S4. Distributions of trapping durations and zone sizes for KRAS in Caco-2 cells

Distributions of trapping durations (A) and trapping zone sizes (B) for KRAS WT and oncogenic mutants are shown before and 2-5 min after EGF stimulation in Caco-2 cells. The size distribution is presented as both violin plots and box-and-whisker plots, indicating the sample median, sample mean (circle), first and third quartiles, and whiskers extending to a maximum of 1.5× interquartile range beyond the box. The zone sizes of individual KRAS trapping events are plotted against trapping durations in (C), with Spearman's rank correlation coefficient (ρ). Statistical analyses were performed using Welch's t-test.

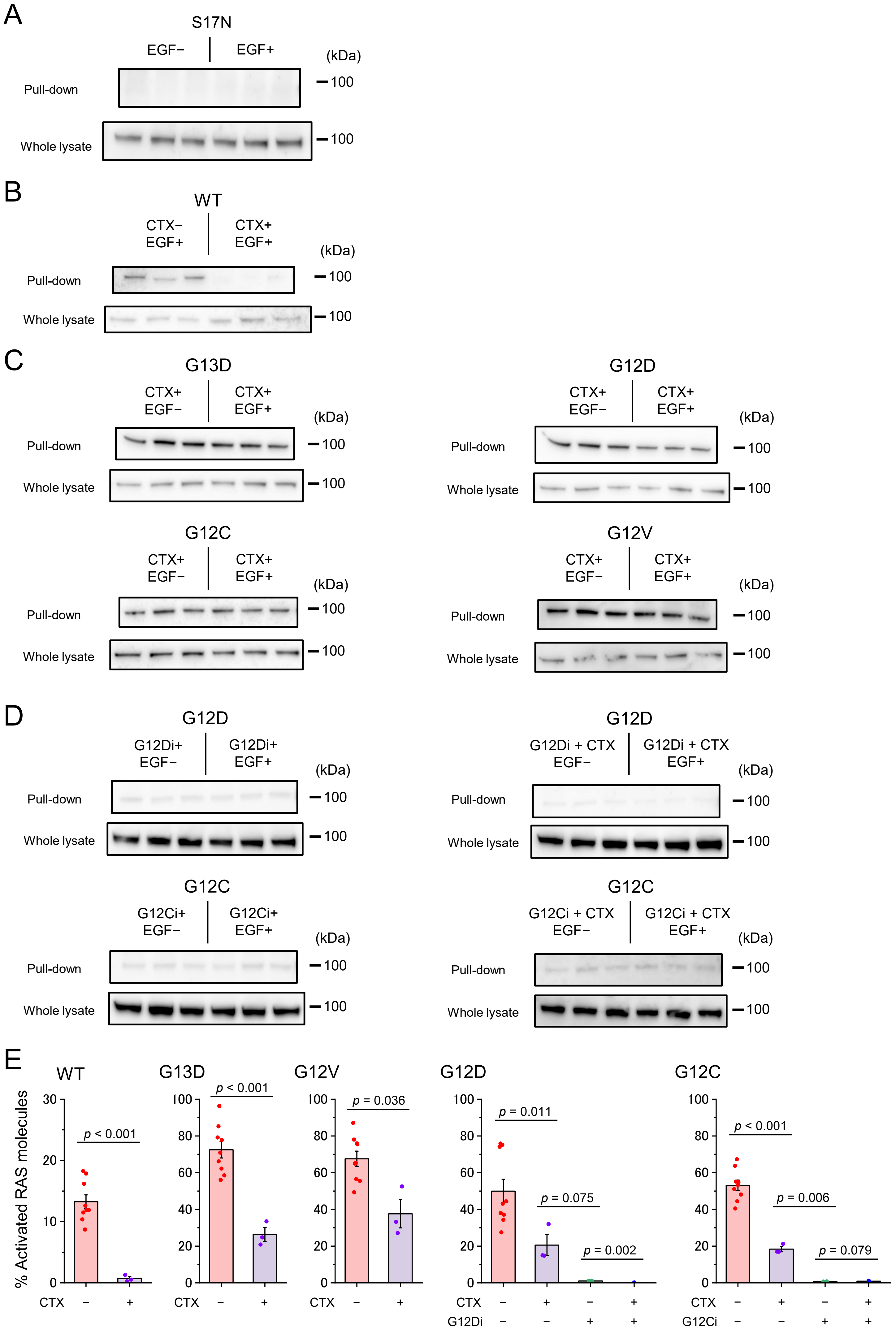
A
S17N
EGF−
EGF+
(kDa)
Pull-down
Whole lysate
100
100
B
WT
CTX−
EGF+
CTX+
EGF+
(kDa)
Pull-down
Whole lysate
100
100
C
G13D
CTX+
EGF−
CTX+
EGF+
(kDa)
Pull-down
Whole lysate
100
100
G12D
CTX+
EGF−
CTX+
EGF+
(kDa)
Pull-down
Whole lysate
100
100
G12C
CTX+
EGF−
CTX+
EGF+
(kDa)
Pull-down
Whole lysate
100
100
G12V
CTX+
EGF−
CTX+
EGF+
(kDa)
Pull-down
Whole lysate
100
100
D
G12D
G12Di+
EGF−
G12Di+
EGF+
(kDa)
Pull-down
Whole lysate
100
100
G12D
G12Di + CTX
EGF−
G12Di + CTX
EGF+
(kDa)
Pull-down
Whole lysate
100
100
G12C
G12Ci+
EGF−
G12Ci+
EGF+
(kDa)
Pull-down
Whole lysate
100
100
G12C
G12Ci + CTX
EGF−
G12Ci + CTX
EGF+
(kDa)
Pull-down
Whole lysate
100
100
E
% Activated RAS molecules
WT
p < 0.001
0
10
20
30
CTX
−
+
G13D
p < 0.001
0
20
40
60
80
100
CTX
−
+
G12V
p = 0.036
0
20
40
60
80
100
CTX
−
+
G12D
p = 0.011
p = 0.075
p = 0.002
0
20
40
60
80
100
CTX
− + − +
G12Di
− − + +
G12C
p < 0.001
p = 0.006
p = 0.079
0
20
40
60
80
100
CTX
− + − +
G12Ci
− − + +

Figure S5. Evaluation of KRAS activation levels under molecular-targeted drug treatments via Western blotting, related to Figure 7

Activated tdStayGold-KRAS proteins, isolated using a RAS-GTP pull-down assay, along with total tdStayGold-KRAS proteins (including both active and inactive forms) from whole-cell lysates, were analyzed by Western blot in SW48 cells.

(A) Dominant-negative KRAS S17N before and after EGF stimulation.

(B) KRAS WT with or without cetuximab treatment 3.5 min after EGF stimulation, related to Figure 7F.

(C) Oncogenic KRAS mutants before and 3.5 min after EGF stimulation under cetuximab treatment, related to Figure 7F.

(D) KRAS G12D and G12C before and 3.5 min after EGF stimulation under KRAS inhibitor monotherapy or combination therapy with KRAS inhibitor and cetuximab.

(E) Percentage of activated KRAS molecules with or without molecular-targeted drug treatment 3.5 min after EGF stimulation, quantified from Western blot analyses (see also Figures 1B and 2A). Data from independent experiments are shown, with bars representing the mean ± SEM. Statistical analyses were performed using Welch's t-test.

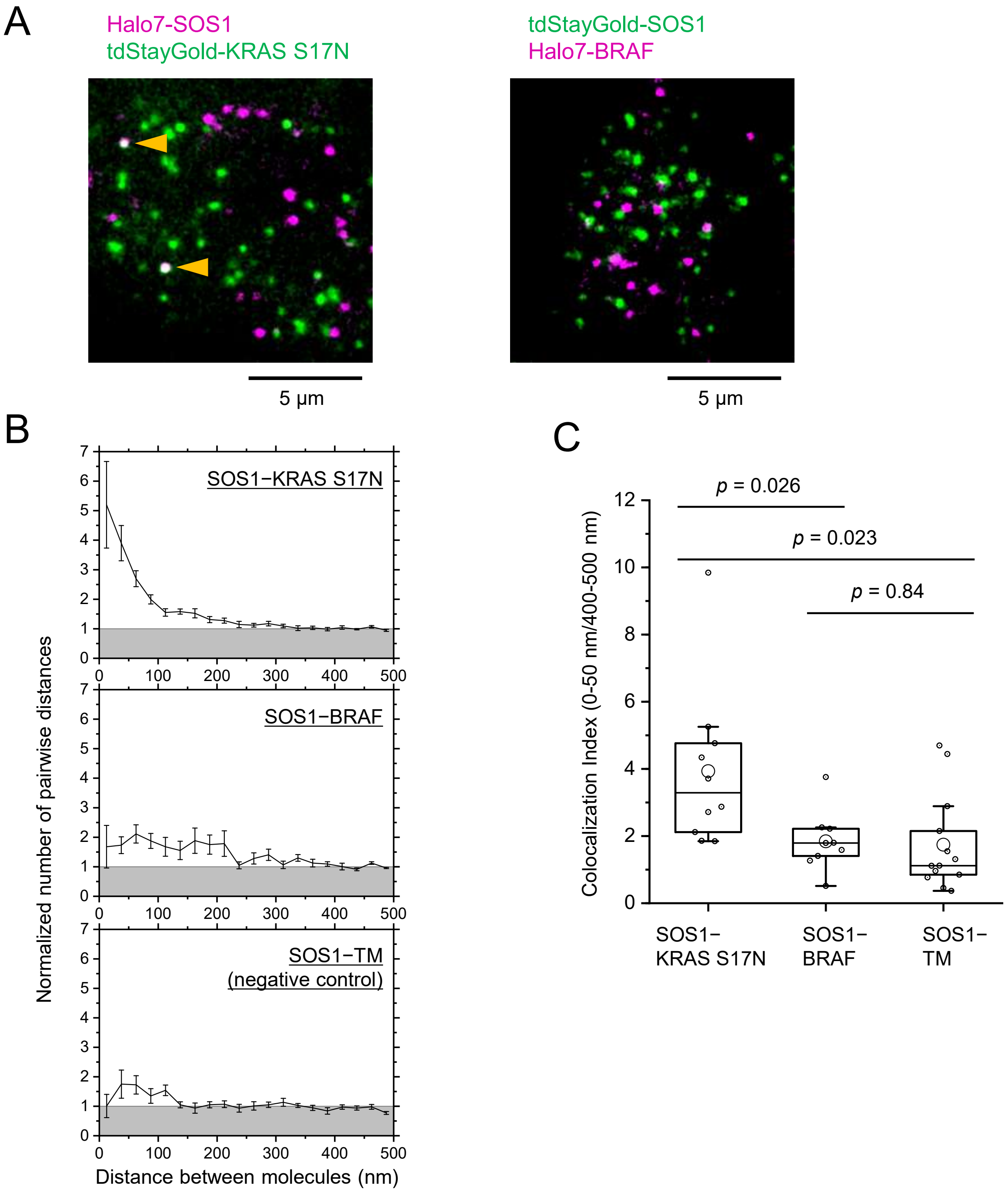

Figure S6. Colocalization analysis of SOS1, BRAF, and KRAS S17N on the PM using simultaneous dual-color single-molecule imaging, related to Figure 5

(A) Representative snapshots of simultaneous dual-color single-molecule imaging of TMR-Halo7-SOS1 (magenta) with tdStayGold-KRAS S17N (green) (left), or tdStayGold-SOS1 (green) with TMR-Halo7-BRAF (magenta) (right) after EGF stimulation in SW48 cells. Colocalized molecules are indicated by yellow arrowheads.

(B) Probability density analysis for SOS1 and KRAS S17N (top), SOS1 and BRAF (middle), and SOS1 and TM (negative control) (bottom). Normalized distributions of pairwise distances between all detected molecular pairs per unit area are shown. Data at each distance are presented as means ± SE.

(C) Colocalization index, defined as the ratio of molecular pairs within 0–50 nm to those within 400–500 nm in (B), was calculated for each cell and plotted in the graphs. The colocalization index is presented in box-and-whisker plots, displaying the minimum, maximum, sample median, sample mean (circle), first and third quartiles, and whiskers extending to a maximum of 1.5× interquartile range beyond the box. Statistical analysis was performed using Welch's t-test (two-sided).

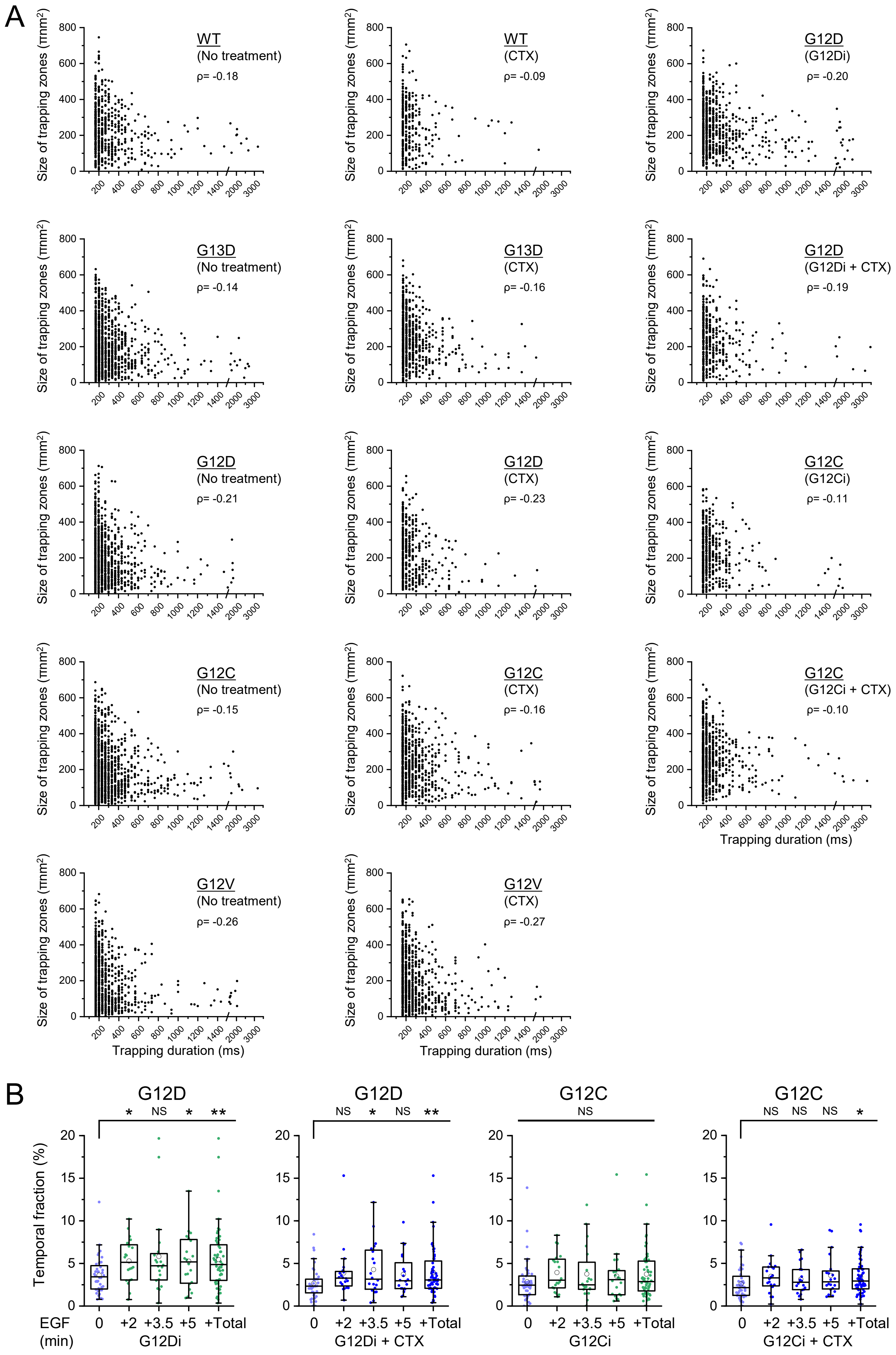
A
WT
(No treatment)
ρ= -0.18
WT
(CTX)
ρ= -0.09
G12D
(G12Di)
ρ= -0.20
G13D
(No treatment)
ρ= -0.14
G13D
(CTX)
ρ= -0.16
G12D
(G12Di + CTX)
ρ= -0.19
G12D
(No treatment)
ρ= -0.21
G12D
(CTX)
ρ= -0.23
G12C
(G12Ci)
ρ= -0.11
G12C
(No treatment)
ρ= -0.15
G12C
(CTX)
ρ= -0.16
G12C
(G12Ci + CTX)
ρ= -0.10
G12V
(No treatment)
ρ= -0.26
G12V
(CTX)
ρ= -0.27
Size of trapping zones (πnm^2)
Trapping duration (ms)
B
G12D
G12D
G12C
G12C
Temporal fraction (%)
EGF (min)
0 +2 +3.5 +5 +Total
G12Di
G12Di + CTX
G12Ci
G12Ci + CTX
NS
*
**

Figure S7. Scatter plots of individual KRAS trapping durations, trapping zone sizes, and temporal trapping fractions under molecular-targeted drug treatments, related to Figures 7 and 8

(A) The zone sizes of individual trapping events for KRAS WT and oncogenic mutants, without molecular-targeted drugs (left, reproduced from Figure 2C, right), with cetuximab treatment (middle, related to Figure 7), or under KRAS inhibitor monotherapy and combination therapy with a KRAS inhibitor and cetuximab (right, related to Figure 8), 2-5 min after EGF stimulation in SW48 cells, are plotted against trapping durations along with Spearman's rank correlation coefficient (ρ).

(B) Time course of temporal trapping fractions for KRAS G12D and G12C 2, 3.5, and 5 min after EGF stimulation under KRAS inhibitor monotherapy or combination therapy with a KRAS inhibitor and cetuximab, related to Figure 8 (+Total includes all data 2, 3.5 and 5 min after stimulation). Data are presented in box-and-whisker plots, displaying the minimum, maximum, sample median, sample mean (circle), first and third quartiles, and whiskers extending to a maximum of 1.5× interquartile range beyond the box. Data after EGF stimulation were compared with data before EGF stimulation using Welch's t-test. *$p < 0.05$ and **$p < 0.01$.

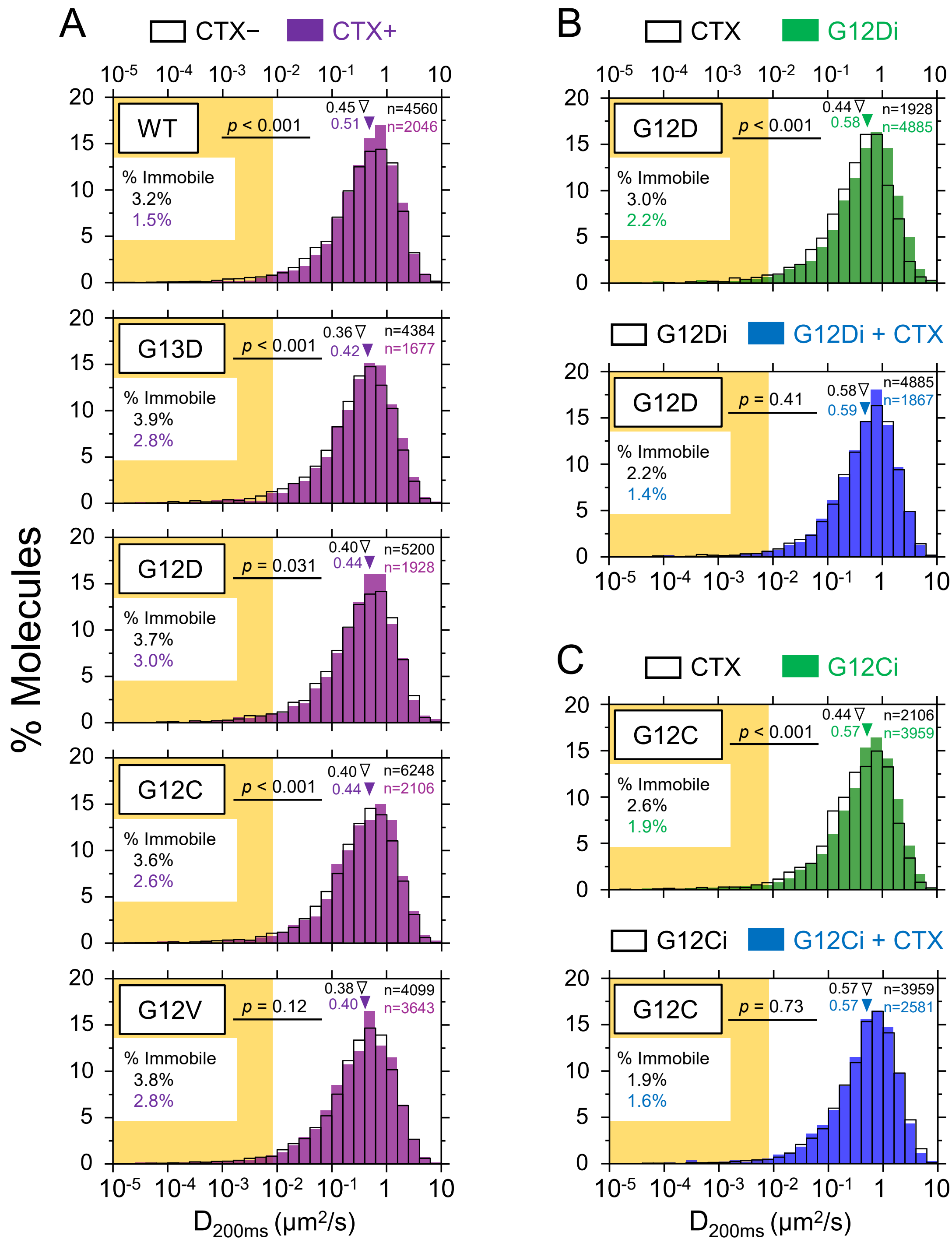

Figure S8. Diffusion coefficients (D_{200ms}) and immobile fractions of KRAS molecules under molecular-targeted drug treatments

Histograms show the distributions of D_{200ms} of KRAS WT and oncogenic mutants under molecular-targeted drug treatments 2-5 min after stimulation, derived from over 1000 trajectories in SW48 cells. The number of trajectories analyzed and immobile fractions are indicated at the top-right and left corners, respectively. Median D_{200ms} values are shown next to the arrowheads.

(A) D_{200ms} of KRAS WT and oncogenic mutants with and without cetuximab treatment 2-5 min after stimulation.

(B and C) D_{200ms} for KRAS G12D (B) and G12C (C) under cetuximab and KRAS inhibitor treatments (top), as well as under KRAS inhibitor monotherapy and combination therapy with a KRAS inhibitor and cetuximab (bottom) 2-5 min after stimulation. Statistical analyses were performed using the Mann-Whitney U test.
