# Supplementary material for: Single-molecule imaging quantifies oncogenic KRAS dynamics for enhanced accuracy of therapeutic efficacy assessment
Source: iScience. 2025 Aug 14;28(9):113374. doi: 10.1016/j.isci.2025.113374 (PMC12424429; doi:10.1016/j.isci.2025.113374)

Figure 1B

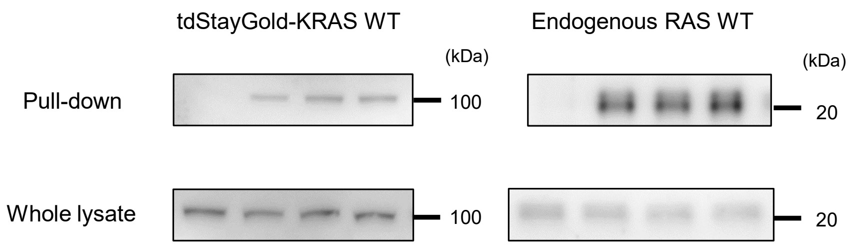
tdStayGold-KRAS WT
Endogenous RAS WT
(kDa)
(kDa)
Pull-down
100
20
Whole lysate
100
20

Figure 1B. tdStayGold-KRAS WT

Luminescence

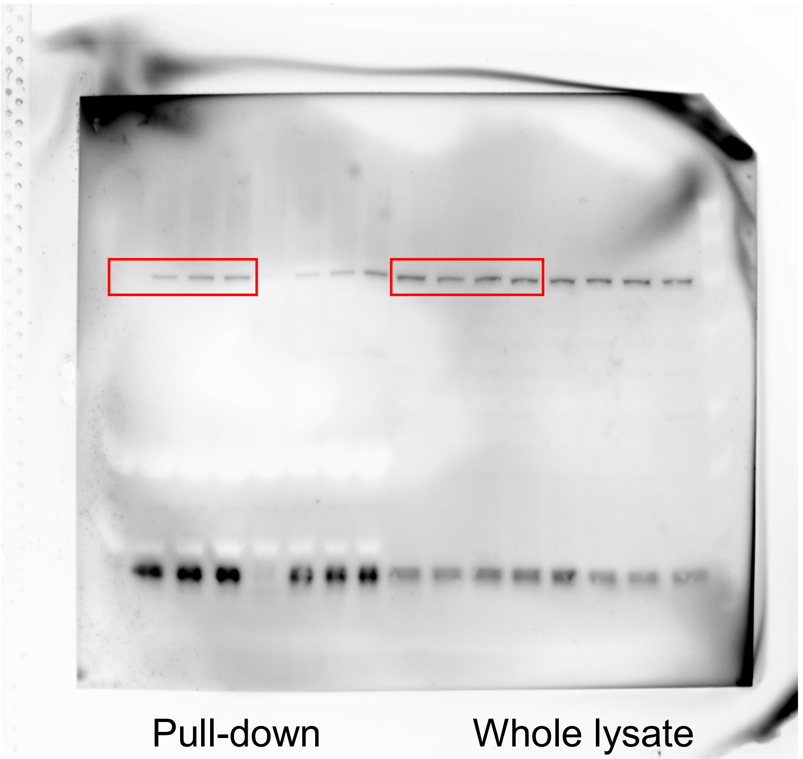

Visible light

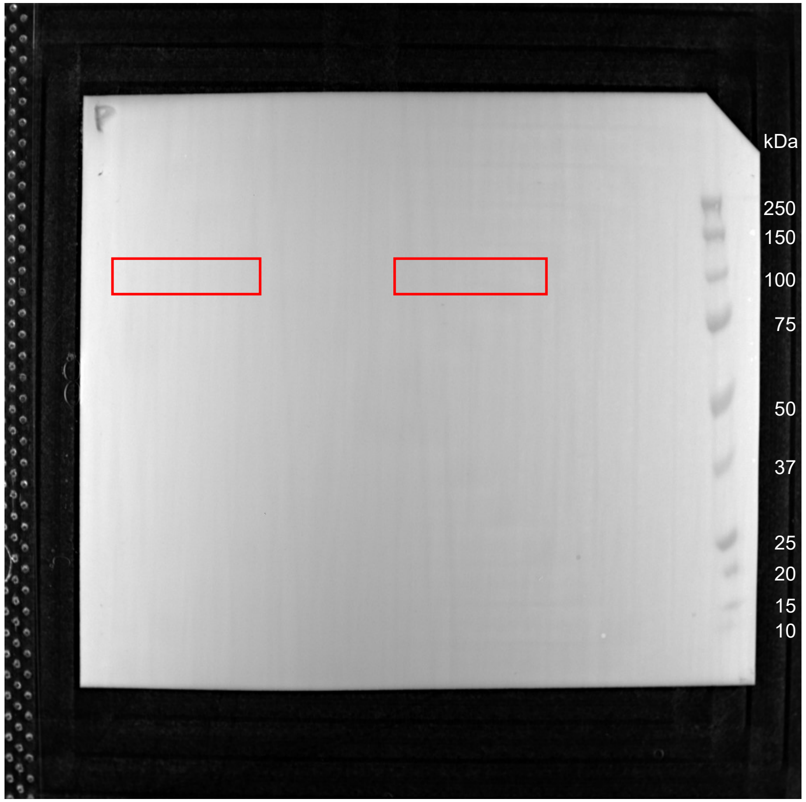

Figure 1B. Endogenous RAS WT

Luminescence

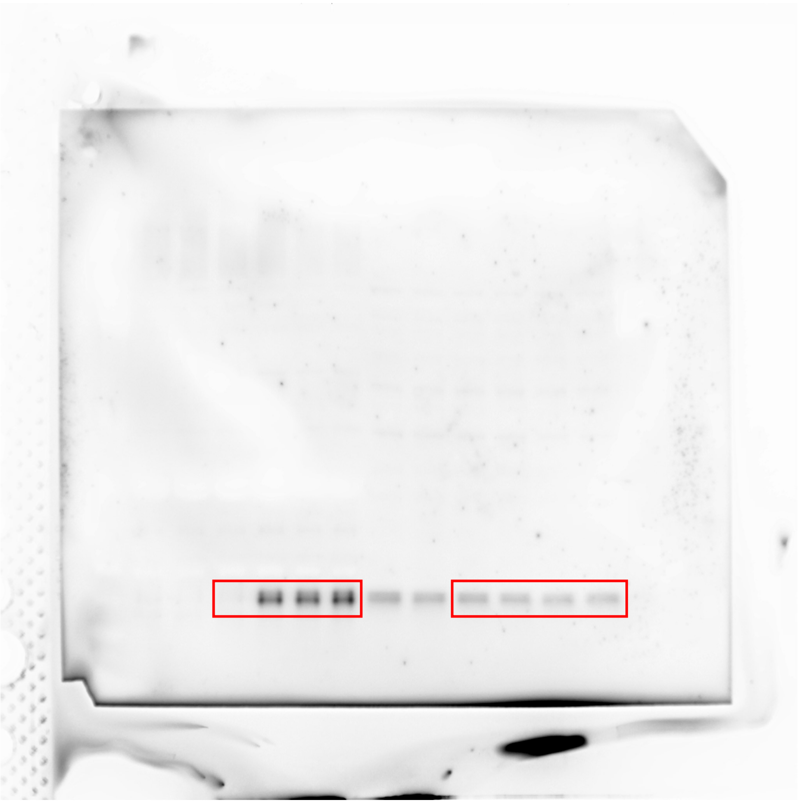

Pull-down Whole lysate

Visible light

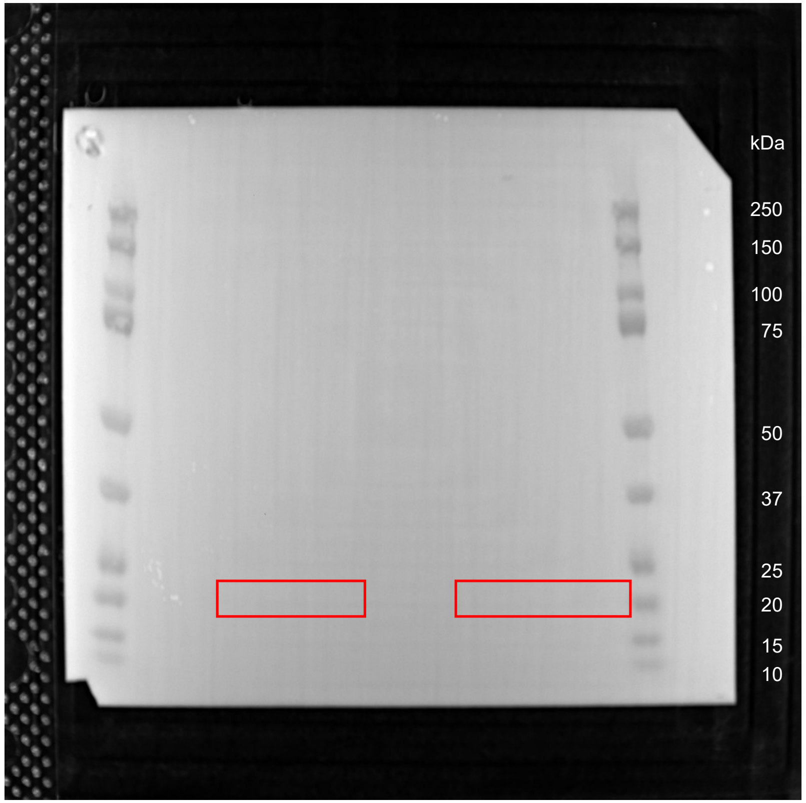

Figure 2A

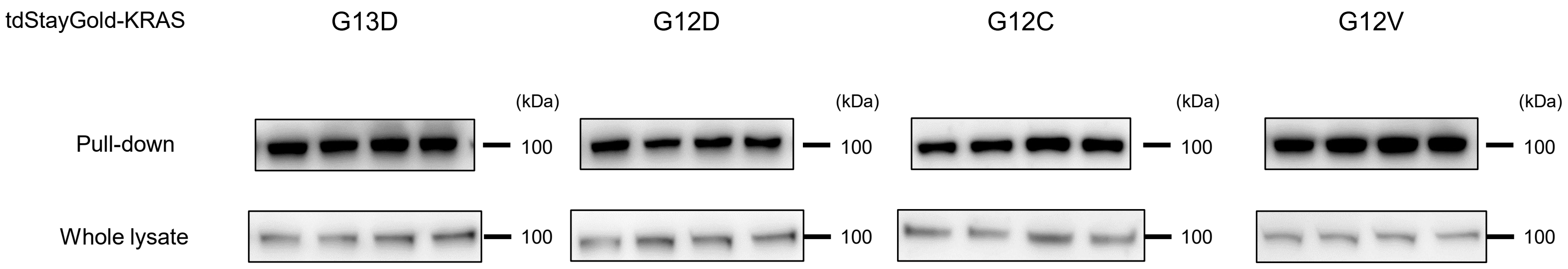
tdStayGold-KRAS
G13D
G12D
G12C
G12V
(kDa)
(kDa)
(kDa)
(kDa)
Pull-down
100
100
100
100
Whole lysate
100
100
100
100

Figure 2A. tdStayGold-KRAS G13D

Luminescence

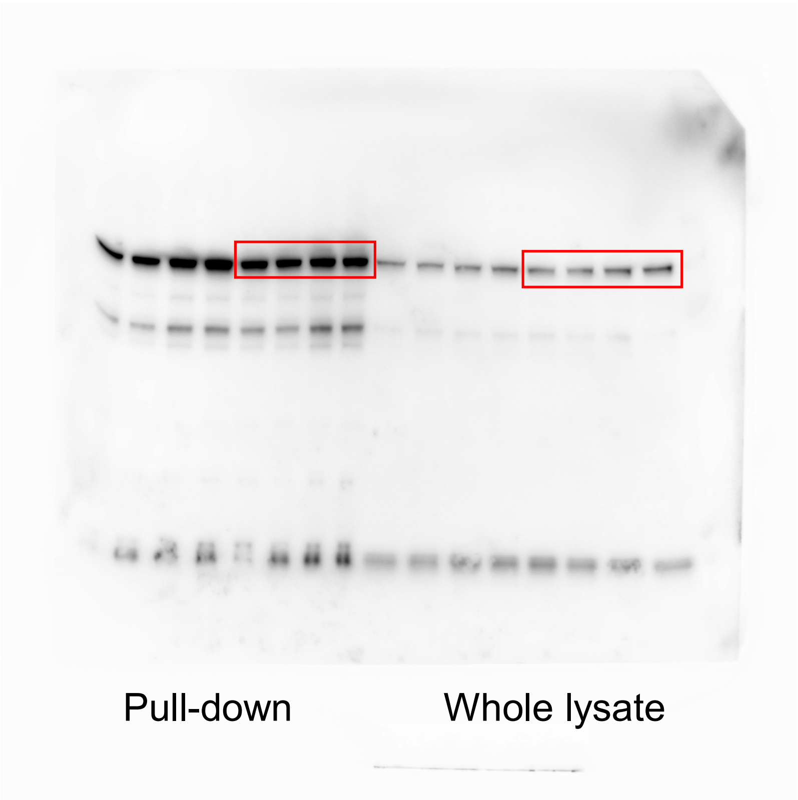

Visible light

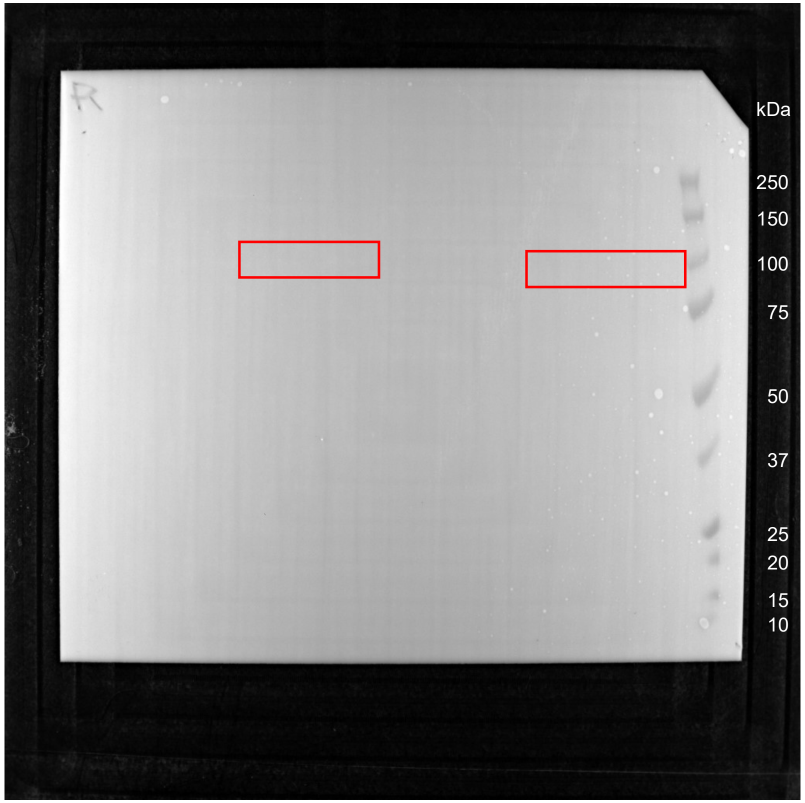

Figure 2A. tdStayGold-KRAS G12D

Luminescence

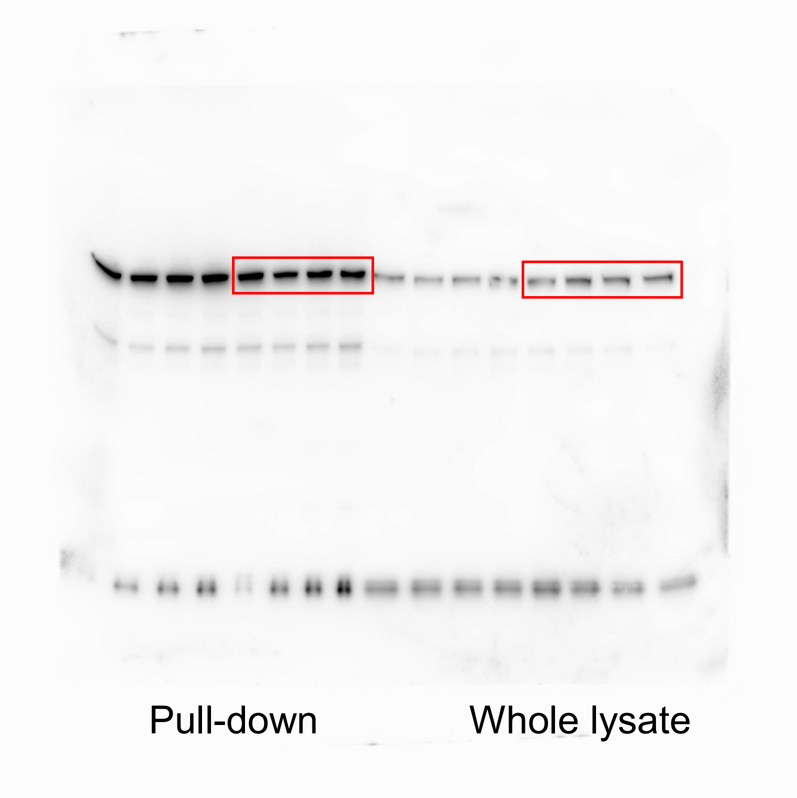

Visible light

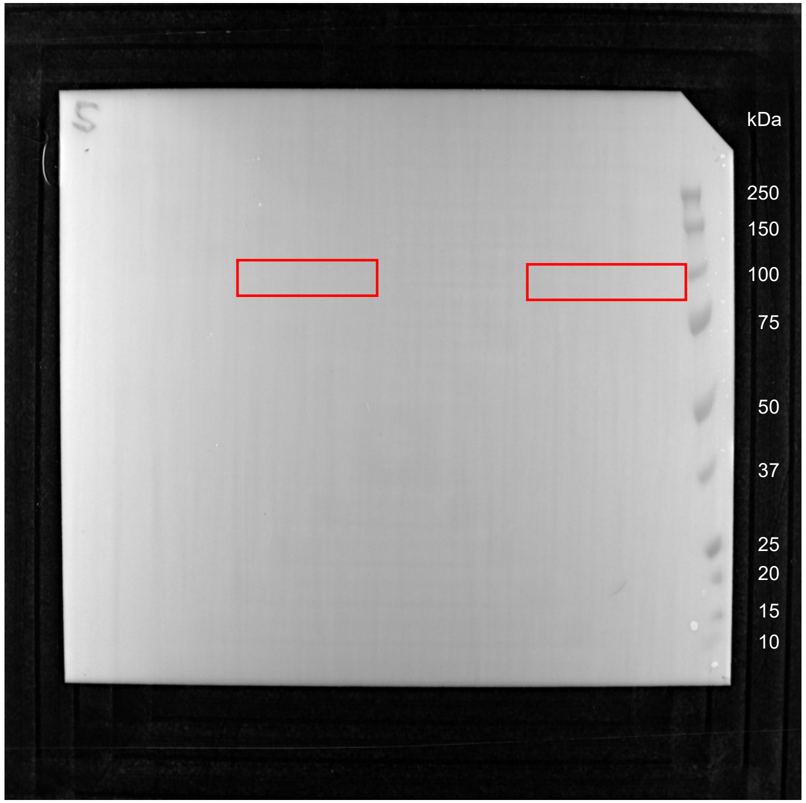

Figure 2A. tdStayGold-KRAS G12C

Luminescence

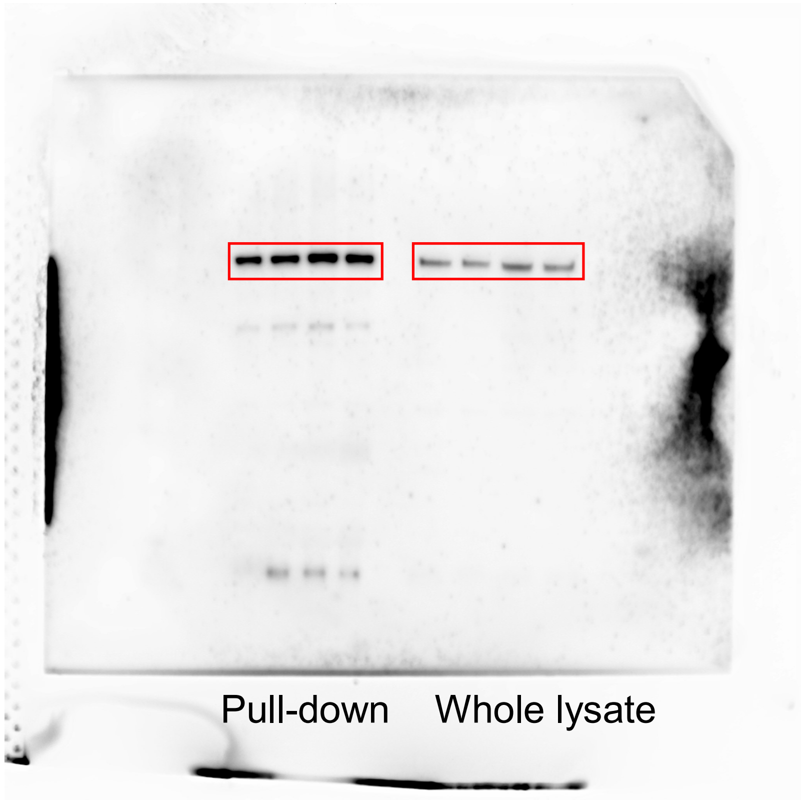

Visible light

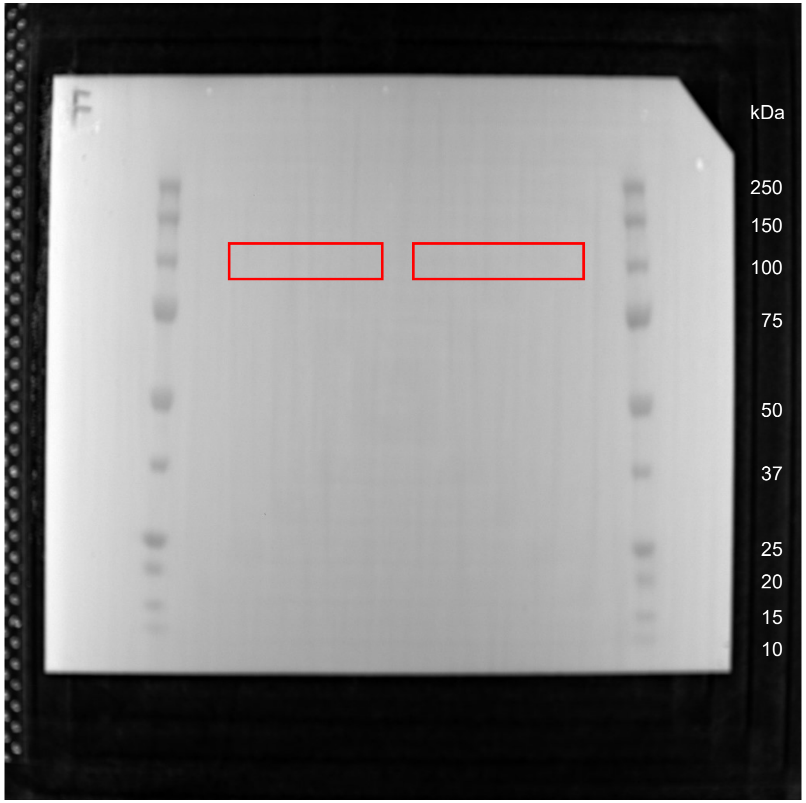

Figure 2A. tdStayGold-KRAS G12V

Luminescence

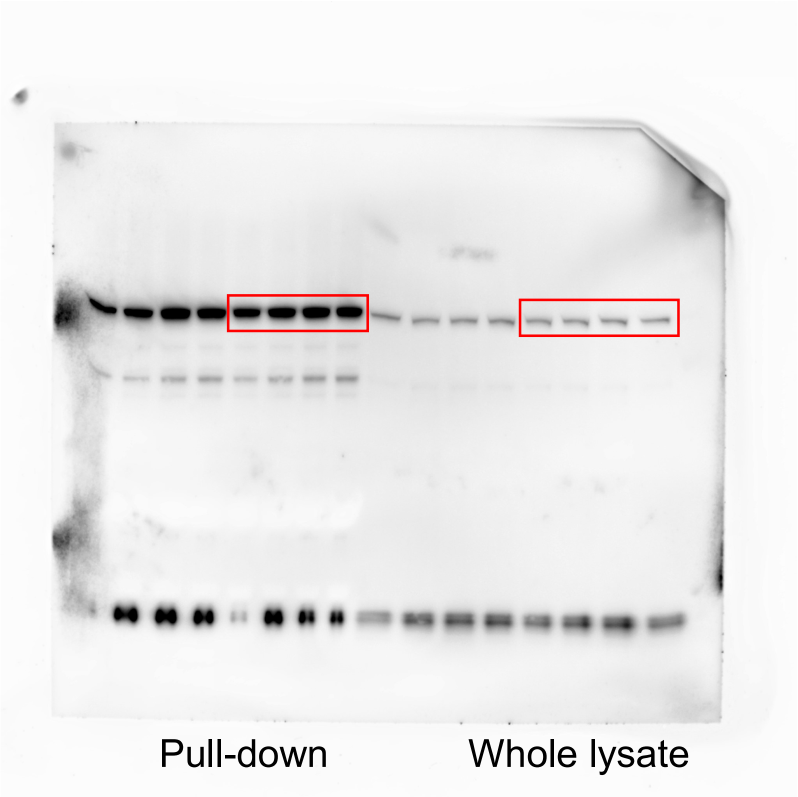

Visible light

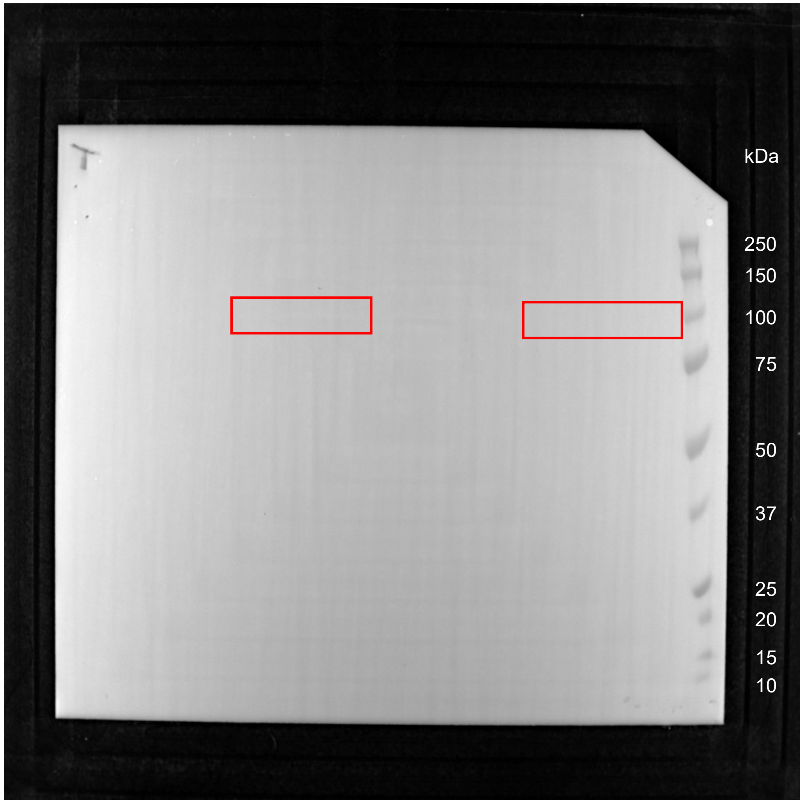

Figure S5

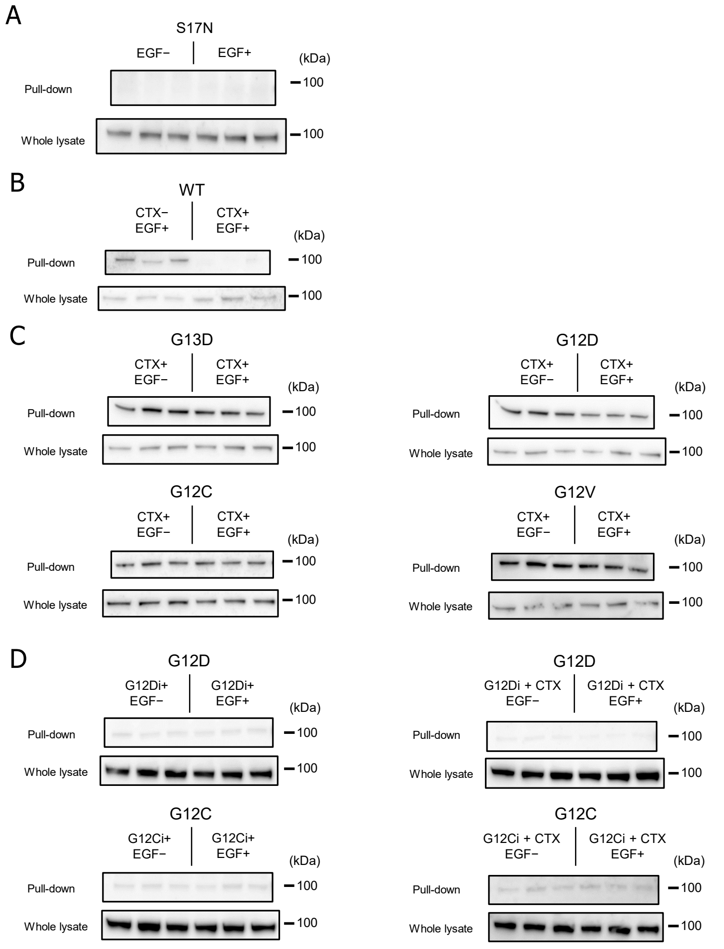
A
S17N
EGF−
EGF+
(kDa)
Pull-down
100
Whole lysate
100
B
WT
CTX−
EGF+
CTX+
EGF+
(kDa)
Pull-down
100
Whole lysate
100
C
G13D
CTX+
EGF−
CTX+
EGF+
(kDa)
Pull-down
100
Whole lysate
100
G12D
CTX+
EGF−
CTX+
EGF+
(kDa)
Pull-down
100
Whole lysate
100
G12C
CTX+
EGF−
CTX+
EGF+
(kDa)
Pull-down
100
Whole lysate
100
G12V
CTX+
EGF−
CTX+
EGF+
(kDa)
Pull-down
100
Whole lysate
100
D
G12D
G12Di+
EGF−
G12Di+
EGF+
(kDa)
Pull-down
100
Whole lysate
100
G12D
G12Di + CTX
EGF−
G12Di + CTX
EGF+
(kDa)
Pull-down
100
Whole lysate
100
G12C
G12Ci+
EGF−
G12Ci+
EGF+
(kDa)
Pull-down
100
Whole lysate
100
G12C
G12Ci + CTX
EGF−
G12Ci + CTX
EGF+
(kDa)
Pull-down
100
Whole lysate
100

Figure S5A. tdStayGold-KRAS S17N

Luminescence

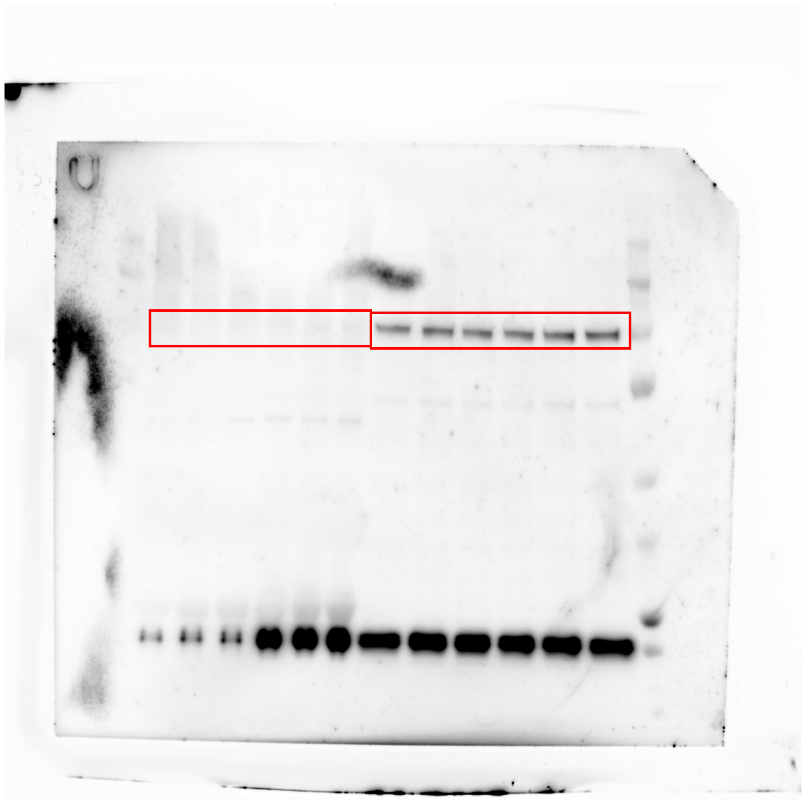

Pull-down Whole lysate

Visible light

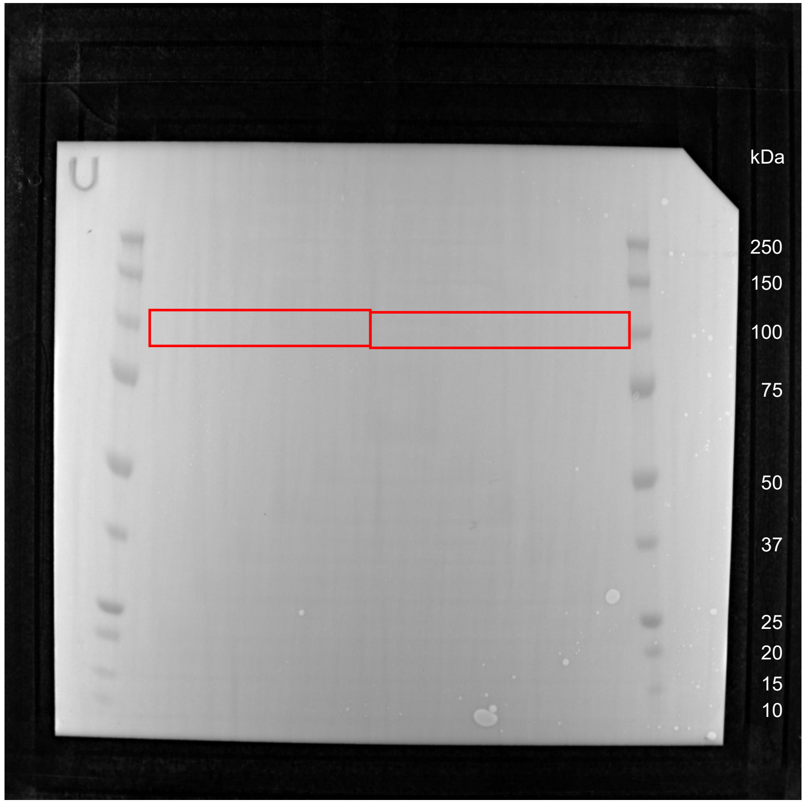

Figure S5B. tdStayGold-KRAS WT with or without cetuximab

Luminescence

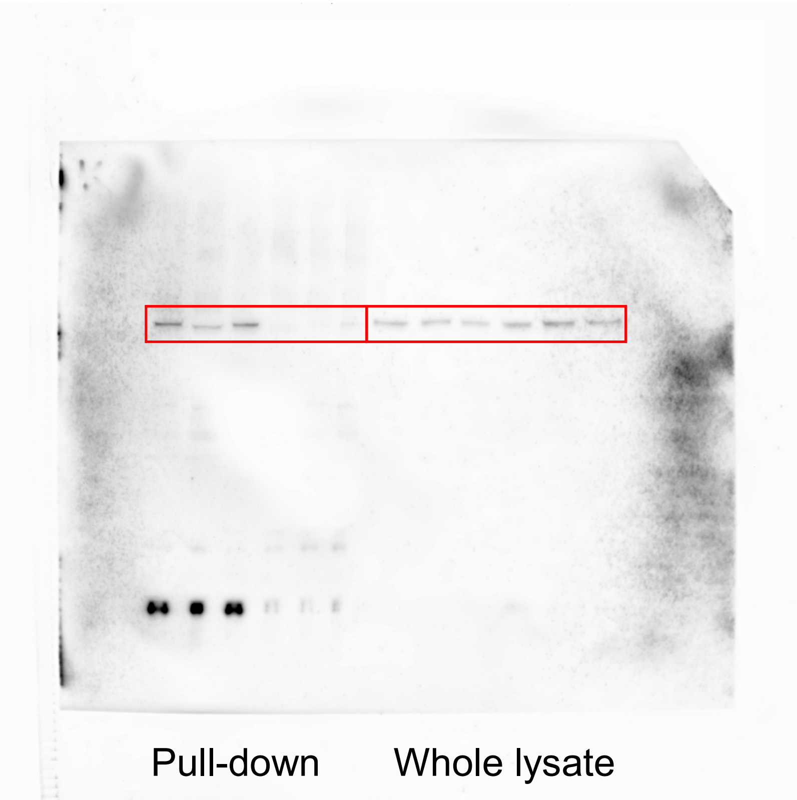

Visible light

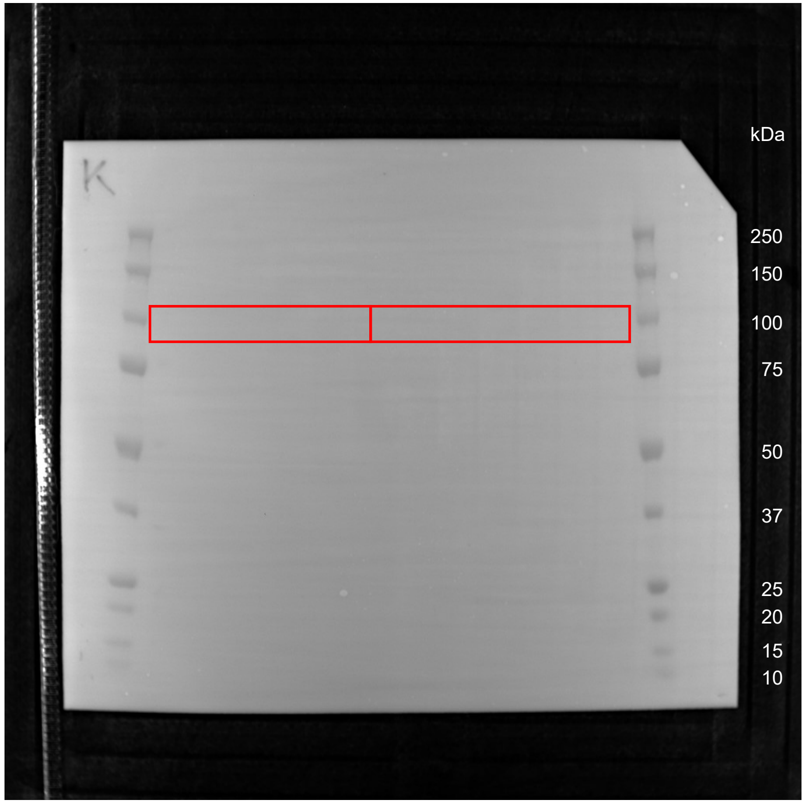

Figure S5C. tdStayGold-KRAS G13D with cetuximab

Luminescence

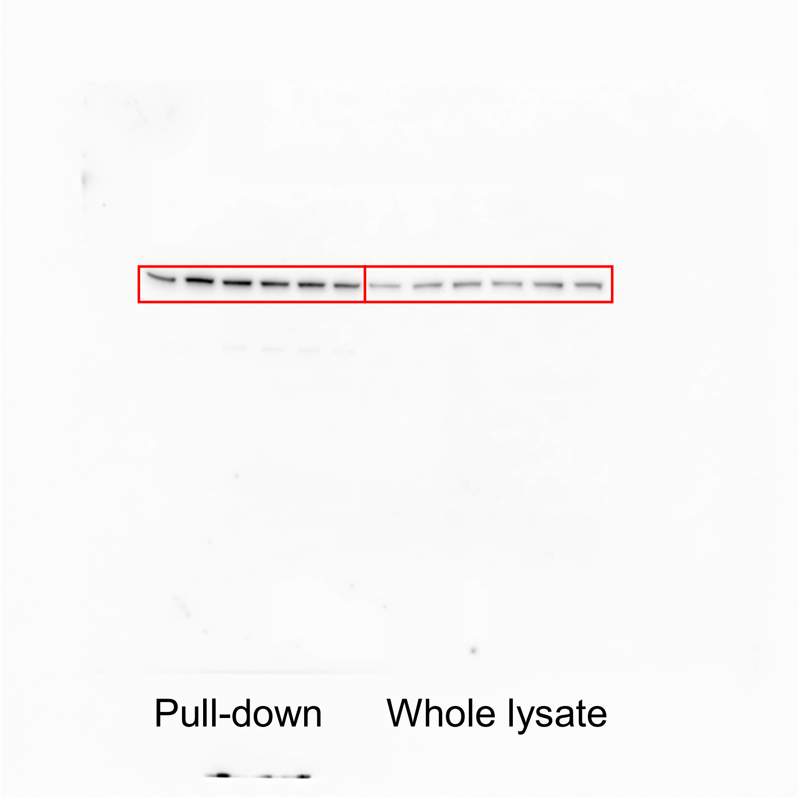

Visible light

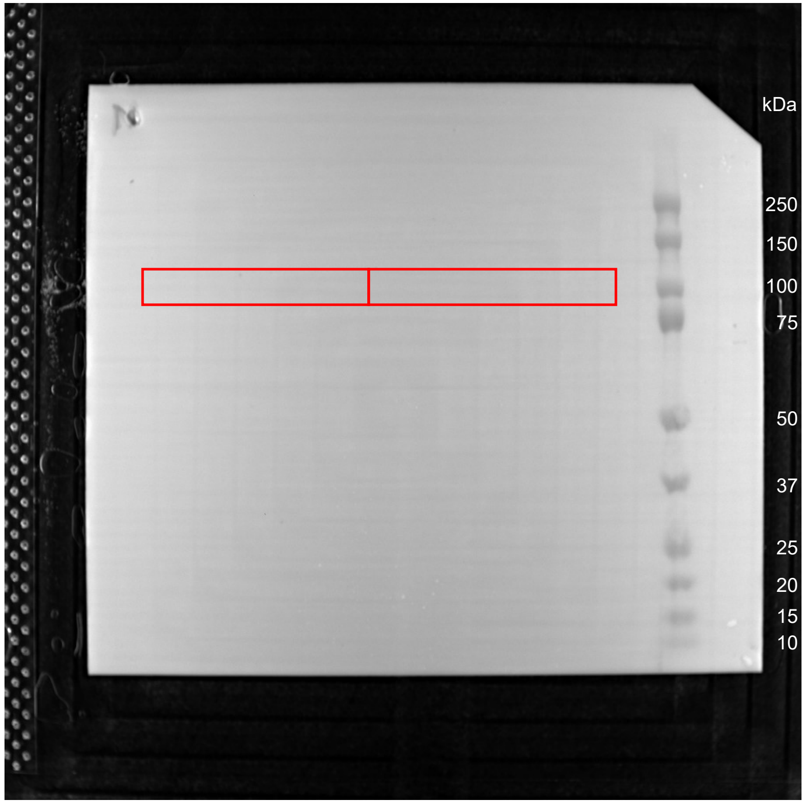

Figure S5C. tdStayGold-KRAS G12D with cetuximab

Luminescence

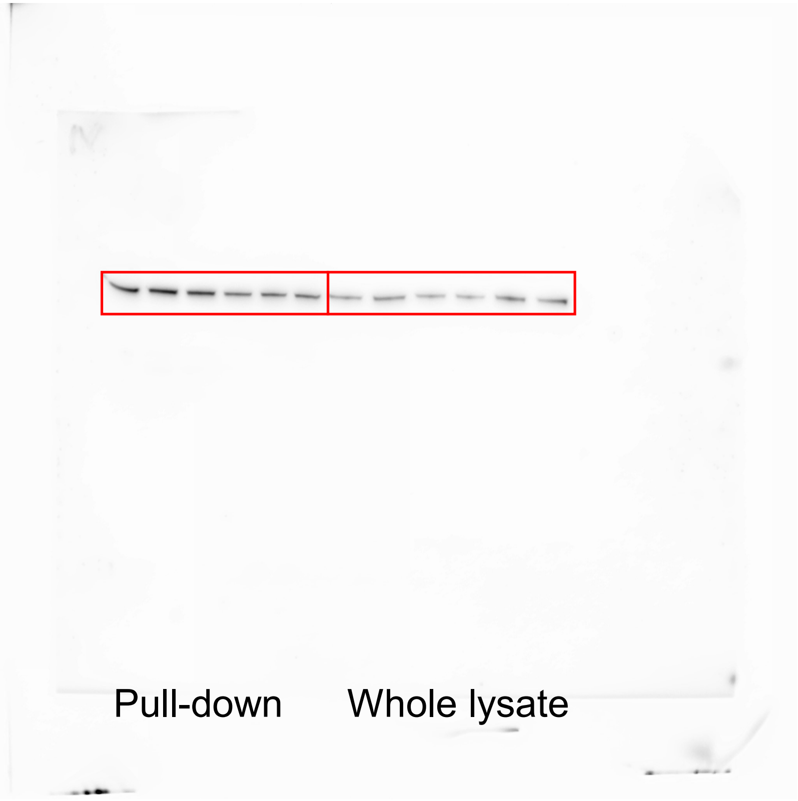

Visible light

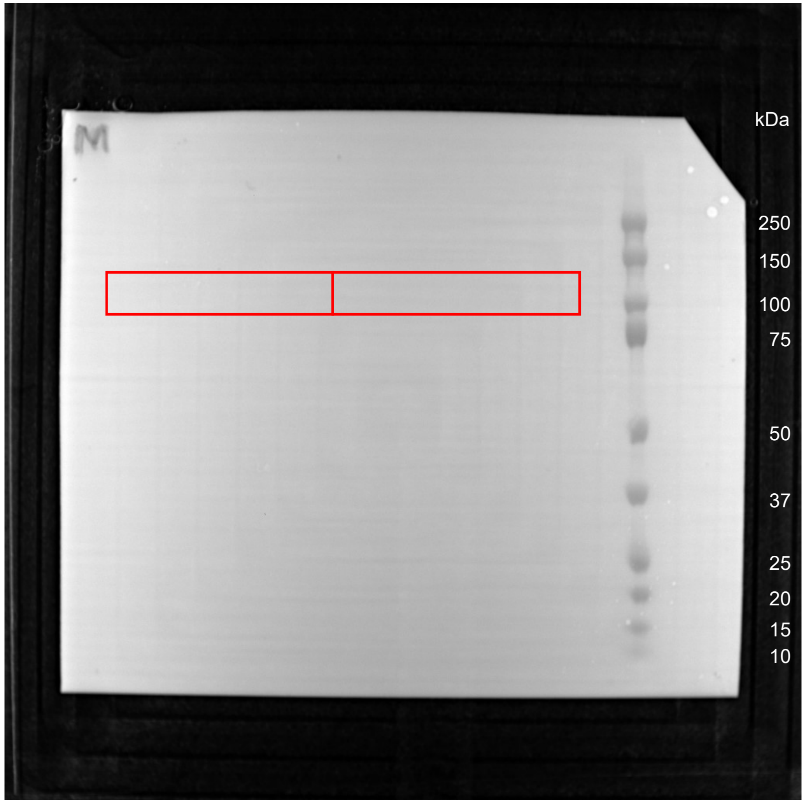

Figure S5C. tdStayGold-KRAS G12C with cetuximab

Luminescence

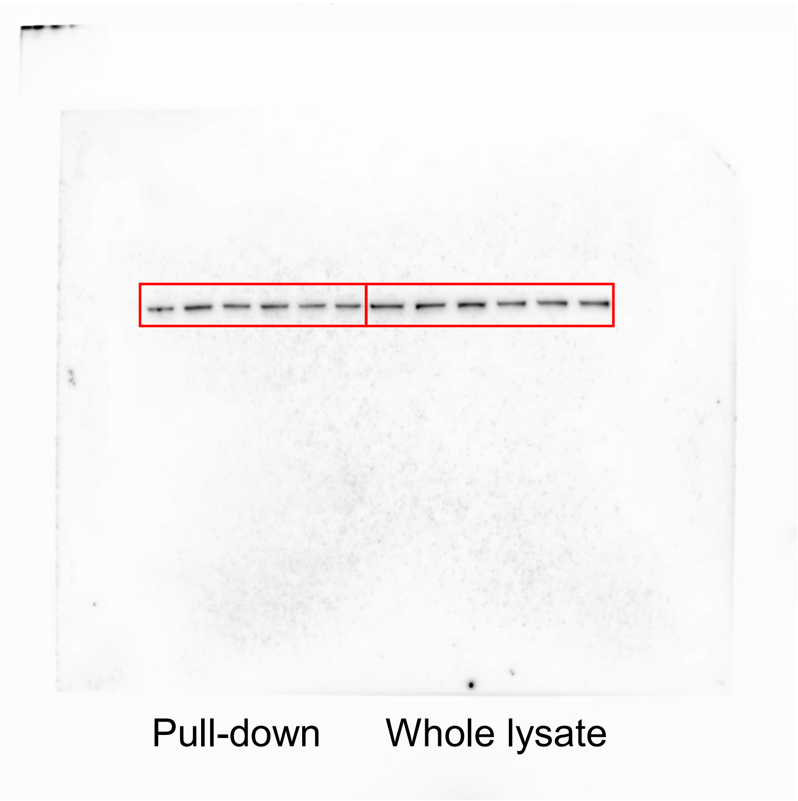

Visible light

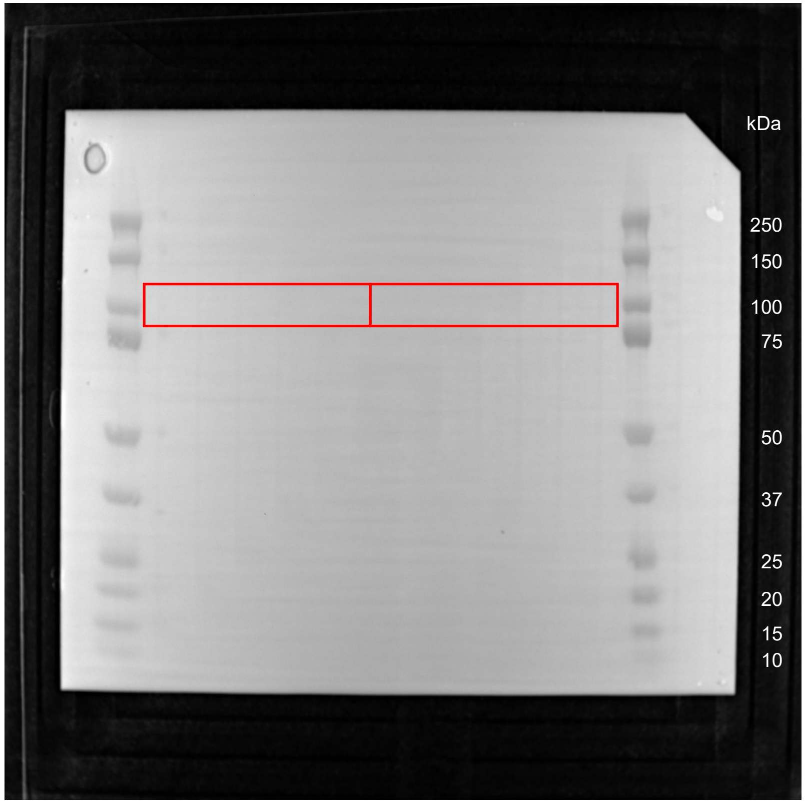

Figure S5C. tdStayGold-KRAS G12V with cetuximab

Luminescence

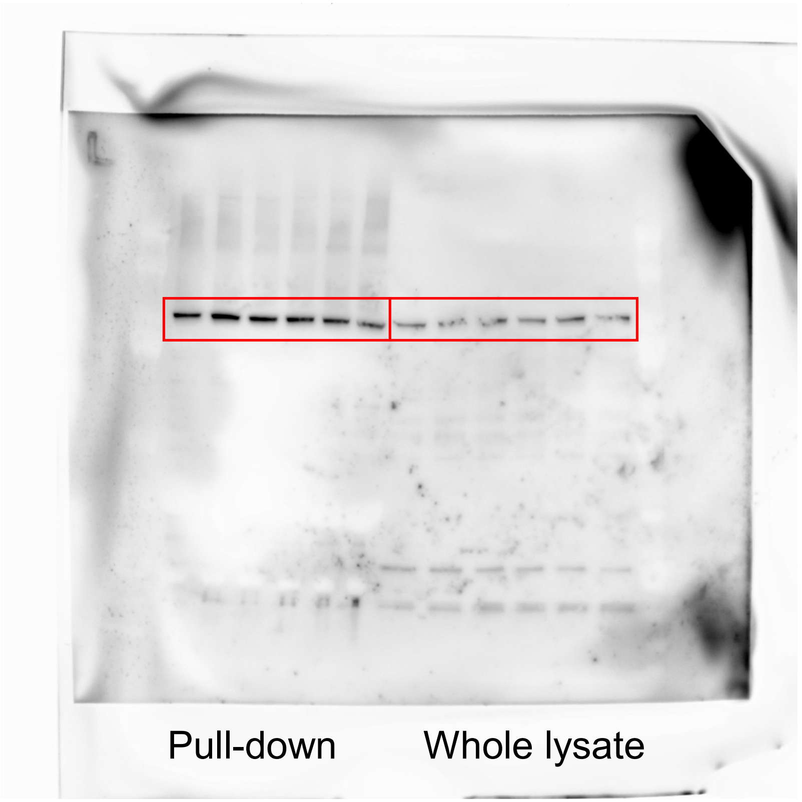

Visible light

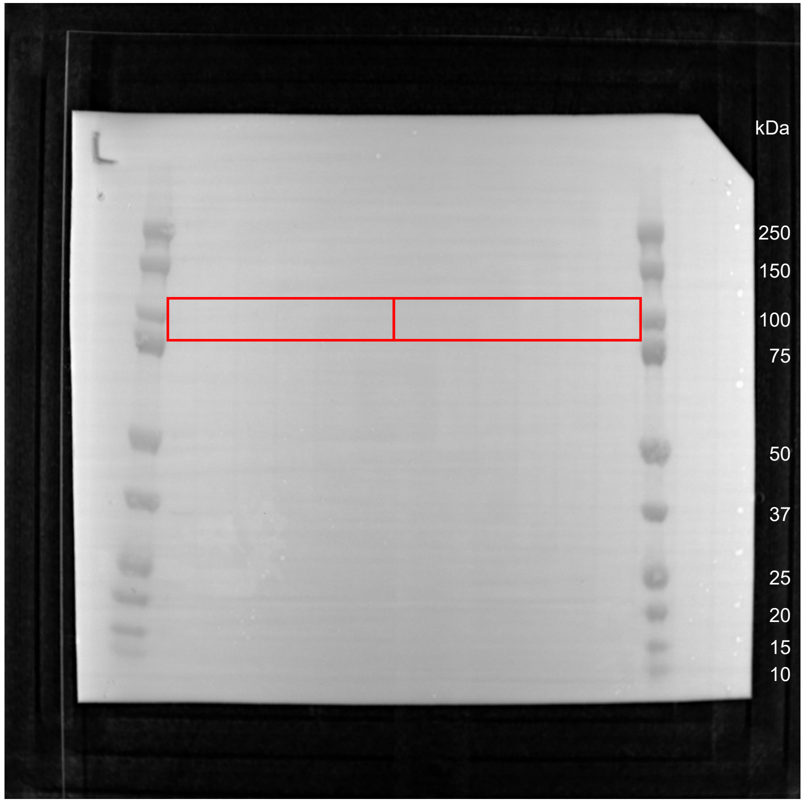

Figure S5D. tdStayGold-KRAS G12D with KRAS G12D inhibitor

Luminescence

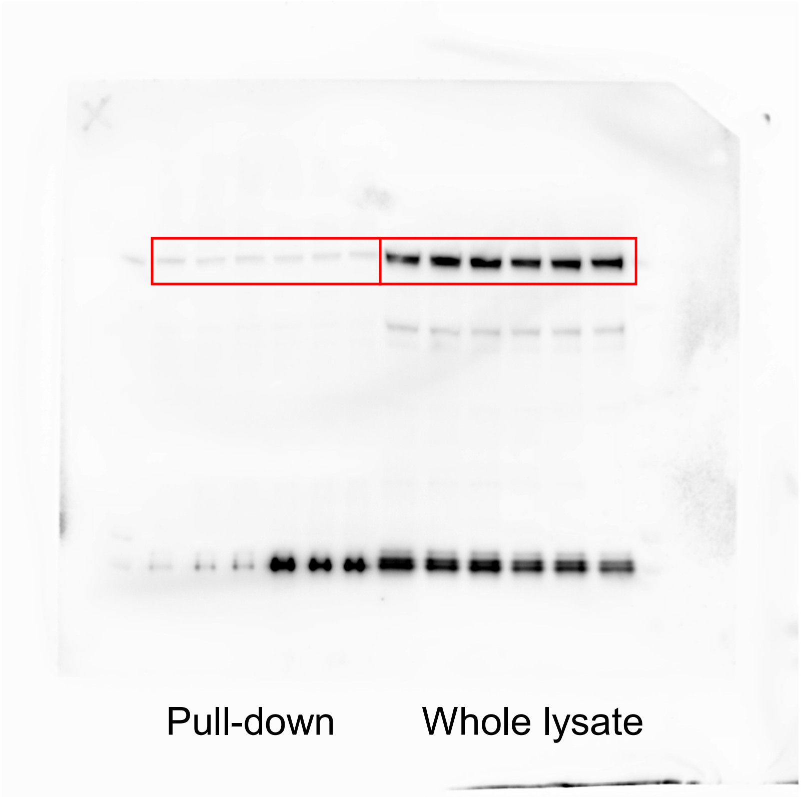

Visible light

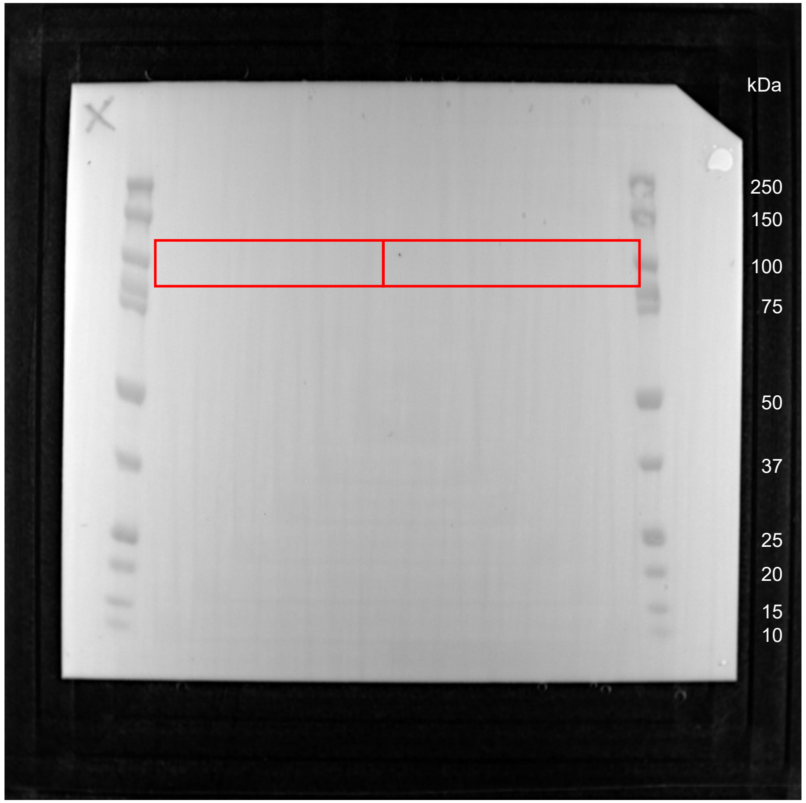

Figure S5D. tdStayGold-KRAS G12D with KRAS G12D inhibitor and cetuximab

Luminescence

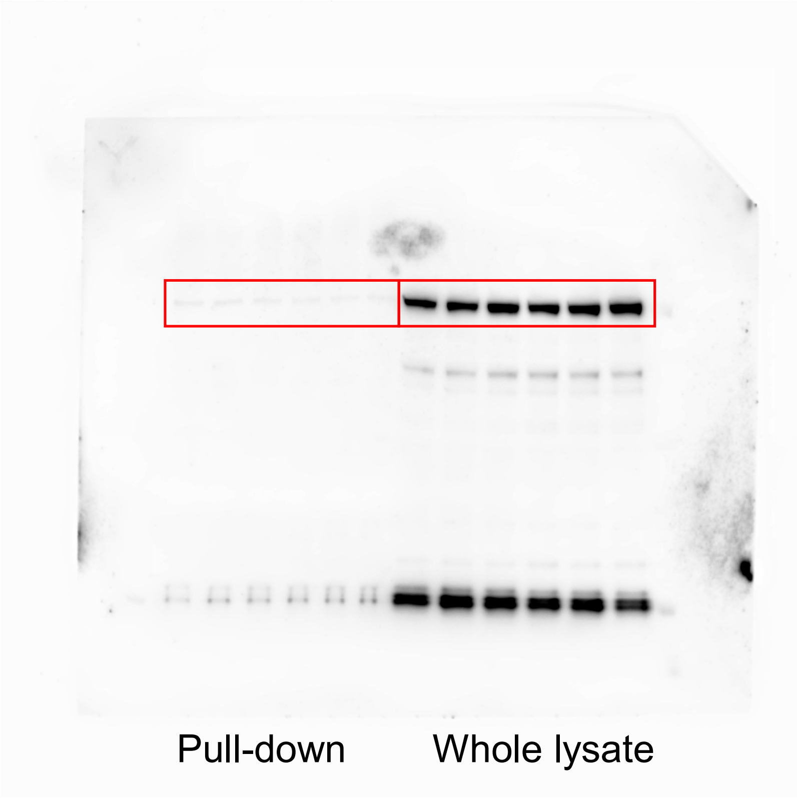

Visible light

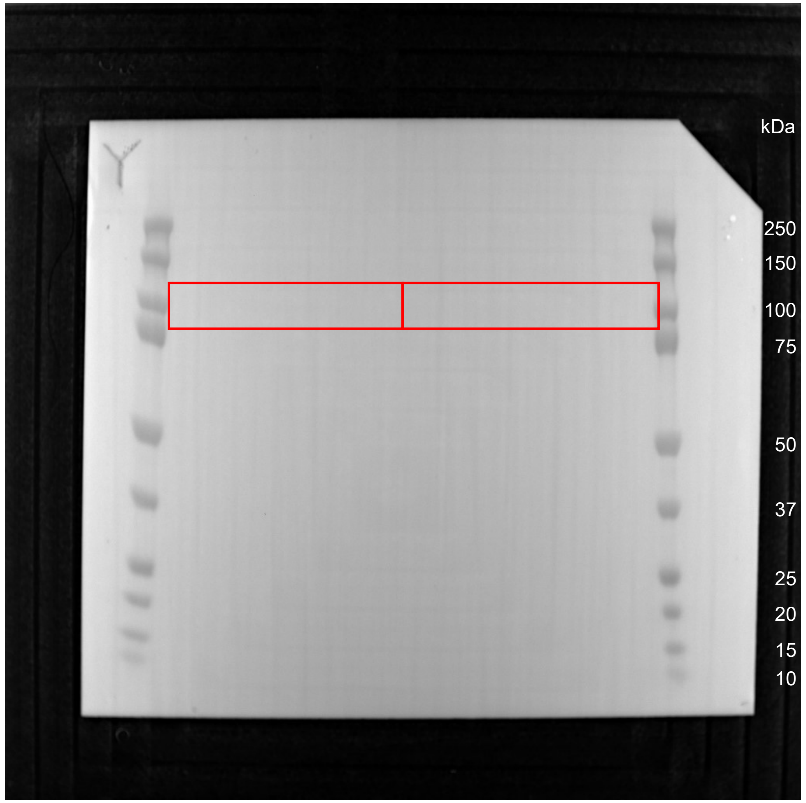

Figure S5D. tdStayGold-KRAS G12C with KRAS G12C inhibitor

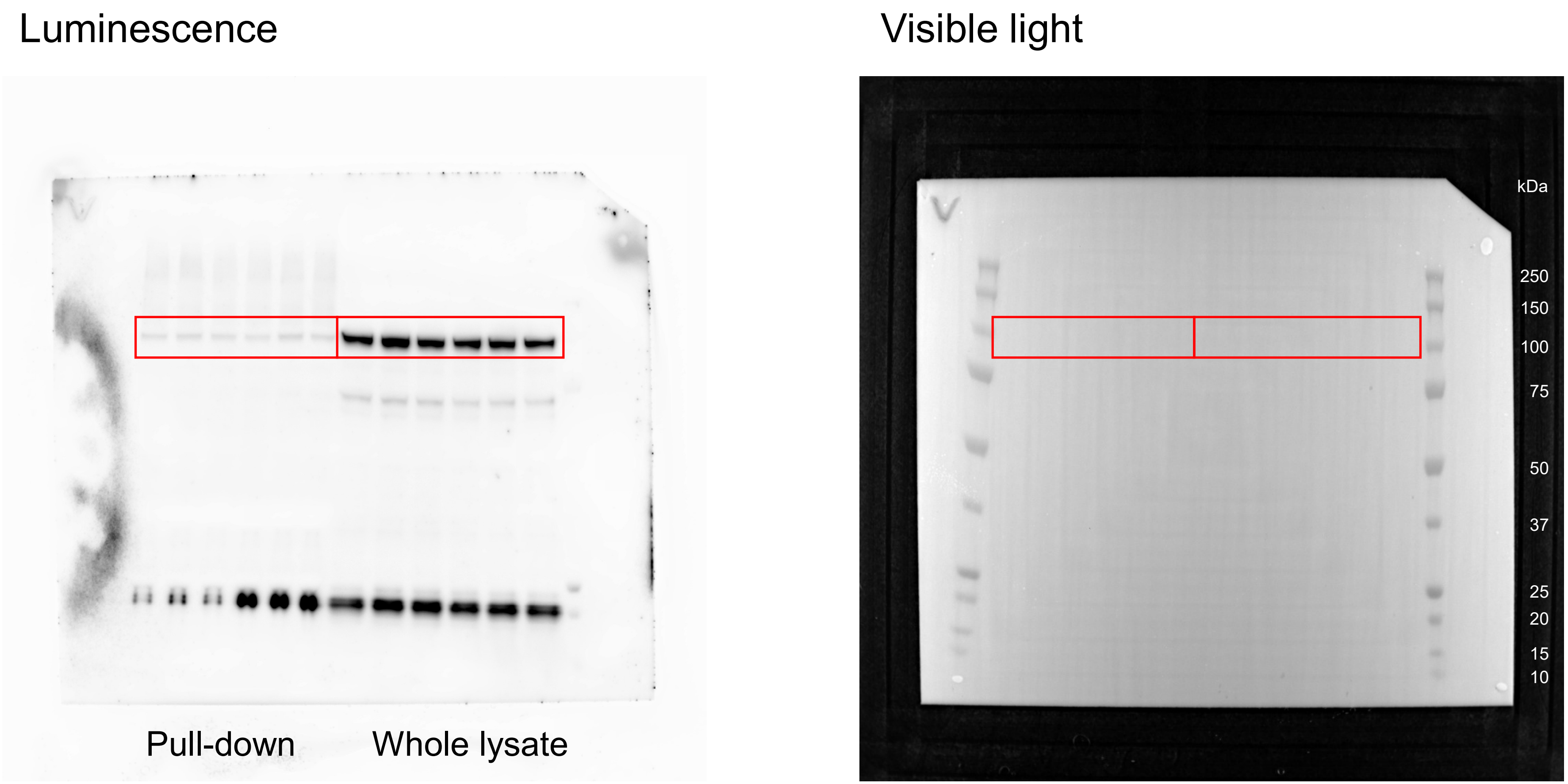

Figure S5D. tdStayGold-KRAS G12C with KRAS G12C inhibitor and cetuximab

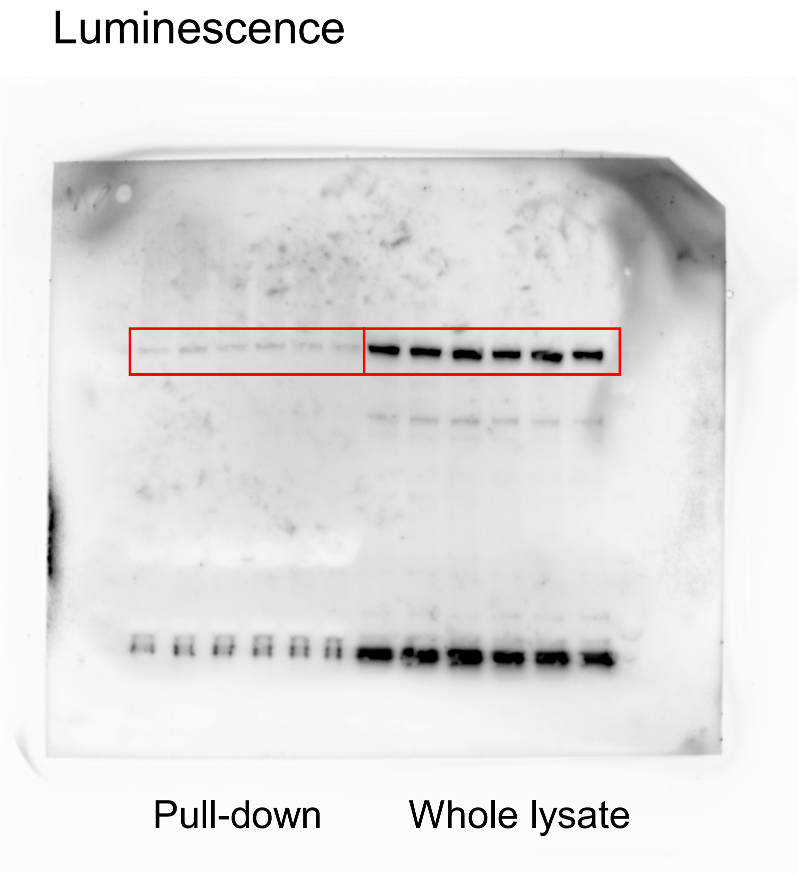

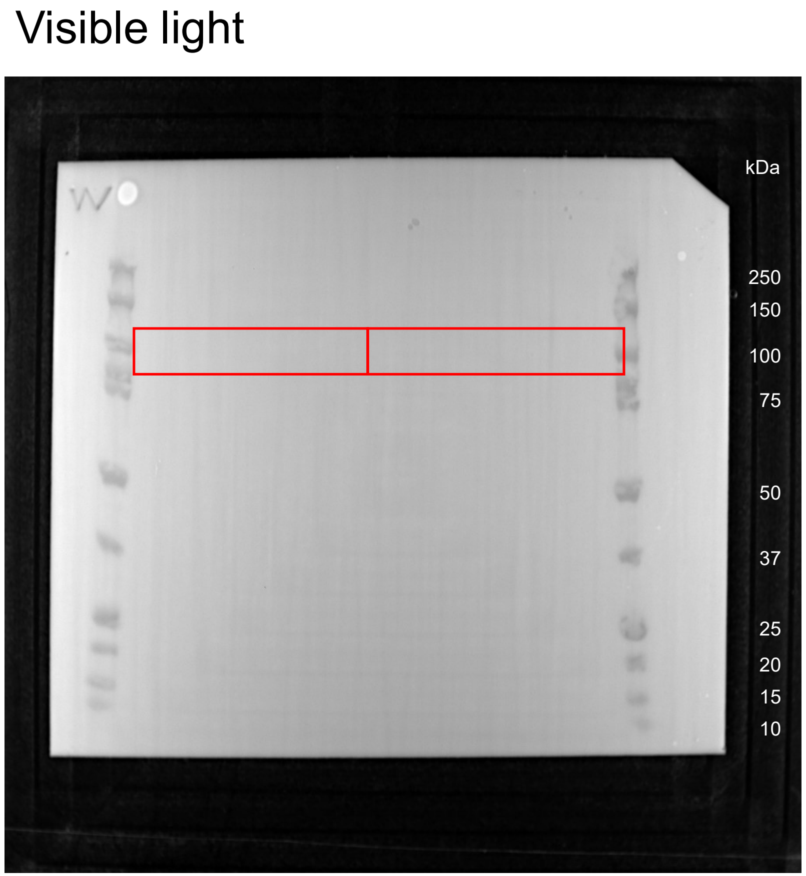

Supplement: Data S1. Source data for western blotting [file mmc2.pdf]
